# The *Staphylococcus aureus* α-Acetolactate Synthase ALS Confers Resistance to Nitrosative Stress

**DOI:** 10.3389/fmicb.2017.01273

**Published:** 2017-07-11

**Authors:** Sandra M. Carvalho, Anne de Jong, Tomas G. Kloosterman, Oscar P. Kuipers, Lígia M. Saraiva

**Affiliations:** ^1^Instituto de Tecnologia Química e Biológica NOVA, Universidade Nova de Lisboa Oeiras, Portugal; ^2^Department of Molecular Genetics, Groningen Biomolecular Sciences and Biotechnology Institute, University of Groningen Groningen, Netherlands

**Keywords:** *Staphylococcus aureus*, nitrosative stress, bacterial metabolism, α-acetolactate synthase (ALS), nuclear magnetic resonance (NMR)

## Abstract

*Staphylococcus aureus* is a worldwide pathogen that colonizes the human nasal cavity and is a major cause of respiratory and cutaneous infections. In the nasal cavity, *S. aureus* thrives with high concentrations of nitric oxide (NO) produced by the innate immune effectors and has available for growth slow-metabolizing free hexoses, such as galactose. Here, we have used deep sequencing transcriptomic analysis (RNA-Seq) and ^1^H-NMR to uncover how *S. aureus* grown on galactose, a major carbon source present in the nasopharynx, survives the deleterious action of NO. We observed that, like on glucose, *S. aureus* withstands high concentrations of NO when using galactose. Data indicate that this resistance is, most likely, achieved through a distinct metabolism that relies on the increased production of amino acids, such as glutamate, threonine, and branched-chain amino acids (BCAAs). Moreover, we found that under NO stress the *S. aureus* α-acetolactate synthase (ALS) enzyme, which converts pyruvate into α-acetolactate, plays an important role. ALS is proposed to prevent intracellular acidification, to promote the production of BCAAs and the activation of the TCA cycle. Additionally, ALS is shown to contribute to the successful infection of murine macrophages. Furthermore, ALS contributes to the resistance of *S. aureus* to beta-lactam antibiotics such as methicillin and oxacillin.

## Introduction

*Staphylococcus aureus* is an opportunistic human pathogen that is persistently found in the epithelial cells of the nasal cavity of patients suffering recurrent sinusitis (Weidenmaier et al., 2012). Even in the absence of symptoms, the staphylococcal nasal carriage is a known risk factor as *S. aureus* exhibits high capacity to evade and thwart the innate and adaptive immune responses. Moreover, repeated staphylococcal infections can evolve to life-threatening diseases, such as endocarditis, bloodstream infection, and sepsis (Foster, 2005; Thammavongsa et al., 2015). Unfortunately, antibiotic resistant *S. aureus* strains are commonly found not only in hospital settings but also in the community, and many strains show resistance to the last generation of antibiotics.

**FIGURE 1:**
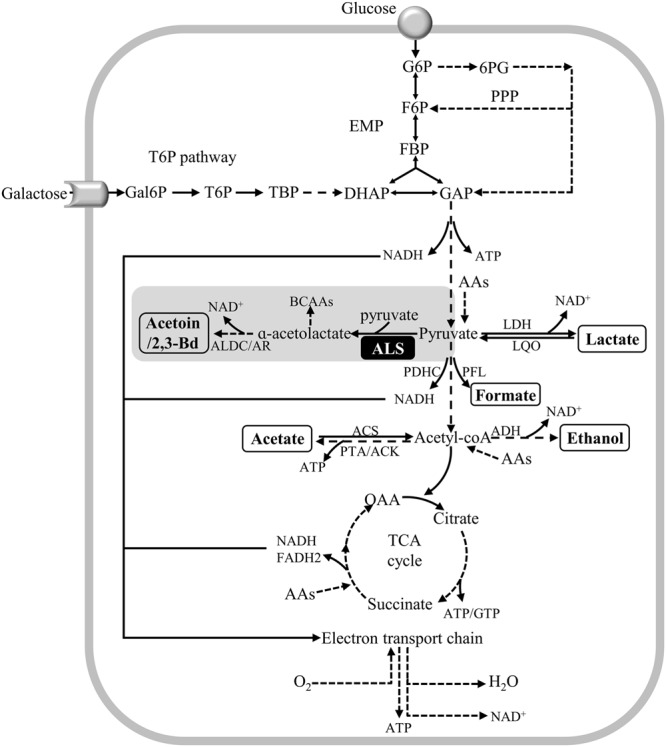
Schematic representation of metabolic pathways of *Staphylococcus aureus* glucose and galactose catabolism. Galactose is catabolized in the tagatose 6-phosphate pathway into the glycolytic intermediates DHAP and GAP. DHAP and GAP originated from galactose and glucose are oxidized to pyruvate in the EMP (glycolysis) pathway, which can be reduced to lactate, α-acetolactate and/or acetyl-CoA. Acetyl-CoA can be converted to acetate, to generate ATP, and ethanol. Alternatively, acetyl-CoA can be used to produce citrate for the citric acid cycle (TCA cycle). Under aerobic conditions, the NADH molecules formed in the EMP and TCA cycle are oxidized in the respiratory chain, thereby restoring the redox balance, and producing ATP. Proposed pathways were reconstructed based on genome information (http://www.ncbi.nlm.nih.gov/genomes/lproks.cgi), literature and database surveys (KEGG, MetaCyc). Inside boxes are the end products of pyruvate metabolism. Inside the black box is the ALS enzyme. AAs, amino acids; ACS, acetyl-CoA synthetase; ADH, bifunctional acetaldehyde-CoA/alcohol dehydrogenase; ALDC/AR, α-acetolactate decarboxylase/acetoin reductase; ALS, α-acetolactate synthase; BCAAs, branched-chain amino acids; DHAP, dihydroxyacetone phosphate; FBP, fructose 1,6-bisphosphate; F6P, fructose 6-phosphate; Gal6P, galactose 6-phosphate; GAP, glyceraldehyde 3-phosphate; G6P, glucose 6-phosphate; LDH, lactate dehydrogenase; LQO, lactate-quinone oxidoreductase; PDHC, pyruvate dehydrogenase complex; PFL, pyruvate formate-lyase; 6-PG, 6-phosphogluconate; PPP, pentose phosphate pathway; PTA/ACK, phosphotransacetylase/acetate kinase; OAA, oxaloacetate; TBP, tagatose 1,6-bisphosphate; T6P, tagatose 6-phosphate.

*Staphylococcus aureus* is a facultative anaerobe that besides the respiratory chain has active Embden-Meyerhof-Parnas (EMP), pentose phosphate pathway (PPP), and citric acid cycle (TCA) pathways (**Figure 1**). As for other pathogens, the synthesis and acquisition of certain nutrients are critical to support the infection capacity of *S. aureus*, glucose being one of the preferred carbon sources of this bacterium. However, in the nasal cavity the glucose levels have been proposed to be substantially lower than those determined in human plasma, and in this niche, disaccharides and other free hexoses are accessible (Krismer et al., 2014). In particular, galactose is one of the major carbohydrates present in the mucus mucins that coat the nasopharyngeal epithelium of the nasal cavity, and studies done with the nasal colonizer *S. pneumoniae* showed that: (i) galactose is released by hydrolysis of mucins promoted by bacterial exoglycosidases; (ii) free galactose is abundantly present in the nasal lavage fluid, whether healthy individuals carry or not the pneumococcus; and (iii) glucose was not detected in *S. pneumoniae* carriers and non-carriers (King, 2010; Blanchette et al., 2016).

In the nose and paranasal sinuses epithelium, the inducible NO synthase, which is expressed in cells of the innate immune system, generates NO. This molecule has antibacterial and cilia-stimulating properties playing a central role in the host immune defenses (Lanz et al., 2008). The infection of human phagocytes by pathogens like *S. aureus* stimulates the NO production. The antimicrobial properties of NO and its derivatives, known as the reactive nitrogen species (RNS), rely on several damaging effects (Richardson et al., 2006; Robinson et al., 2014). Yet, in general, pathogens resist NO through the activation of detoxifying enzymes, repairing proteins and complex metabolic adaptations, and *S. aureus* has an unique ability to thrive in NO rich environments (Gonçalves et al., 2006; Richardson et al., 2008; Tavares et al., 2009; Fuller et al., 2011; Nobre and Saraiva, 2014).

The preferential glycolytic carbon sources of *S. aureus* in the presence of high NO concentrations are hexoses (e.g., glucose and mannose) (Vitko et al., 2015), and NO-exposed glucose-grown cells of *S. aureus* use glycolysis (EMP) as the primary central metabolic pathway (Hochgräfe et al., 2008; Richardson et al., 2008; Fuller et al., 2011; Crooke et al., 2013; Vitko et al., 2015; Spahich et al., 2016). In contrast, gluconeogenic or non-glycolytic carbon sources (e.g., glycerol, fatty acids, amino acids) fail to provide resistance to NO, as this compound impairs the respiratory chain and partially inhibits the PDHC and the PFL activities (Richardson et al., 2008; Vitko et al., 2015) (**Figure 1**). The fluctuating glucose levels occurring in the nasal cavity implies that *S. aureus* has to resort to other carbon sources, which led us to examine how *S. aureus* sustains NO stress when utilizing other hexoses available in the nasal niche (King, 2010; Krismer et al., 2014; Blanchette et al., 2016). In particular, galactose is important for the colonization of *S. aureus*, as studies have shown that: (i) *S. aureus* binds human nasal galactose rich mucins during colonization (Shuter et al., 1996); (ii) *S. aureus* binds galactose molecules in host glycoproteins (Sakarya et al., 2010); (iii) the *S. aureus* tagatose 6-phosphate pathway is essential for the catabolism of lactose/galactose, which are sugars present in milk and derivative products (Miallau et al., 2007); and (iv) the malfunctioning of the *S. aureus* tagatose 6-phosphate pathway leads to accumulation of the dietary D-galactose causing bacterial cell death (Miallau et al., 2007). While in many bacteria, galactose is predominantly metabolized through the Leloir pathway, in *S. aureus* the galactose breakdown occurs through the tagatose 6-phosphate pathway. In this bacterium, galactose is transported and phosphorylated via a galactose phosphotransferase system and converted into the glycolytic intermediates DHAP and GAP (**Figure 1**). In general, the tagatose 6-phosphate pathway is less efficient than the Leloir pathway for the catabolism of galactose, mainly due to the low-affinity for galactose of the phosphotransferase systems and thus, to the slow transport/consumption of the sugar (Neves et al., 2010). In this work, we have analyzed for the first time the effect of NO on cells grown on galactose. We report that although considered a slow-metabolizing sugar, galactose ensures growth of *S. aureus* in the presence of NO by diverting its metabolism to the production of specific amino acids via activation of several enzymes, namely the ALS. In this work, this enzyme is shown to be important for the protection of *S. aureus* against NO and antibiotics.

## Materials and Methods

### Bacterial Strains and Growth Conditions

*Staphylococcus aureus* JE2, a plasmid-cured derivative of the community-associated methicillin-resistant USA300 LAC strain, and the JE2 *alsS* transposon mutant (NE1397, JE2 *alsS*::ΦNΣ; Em^R^) of the Nebraska Transposon Mutant Library (UNMC) were obtained from the Network on Antimicrobial Resistance in *S. aureus* (NARSA, Chantilly, VA, United States). The *alsS* mutation was confirmed by PCR according to the Nebraska-UNMC instructions^1^, and using the following primers: 5′-GCTTTTTCTAAATGTTTTTTAAGTAAATCAAGTAC-3′ and 5′-CATCAAAGTATTGATAATGCTGCG-3′ (*alsS*-specific).

Routinely, *S. aureus* was grown aerobically at 37°C and 150 rpm in TSB broth (Difco), and when required, erythromycin was added at 10 μg mL^-1^. For the transcriptomic and metabolic studies, *S. aureus* was cultured in chemically defined medium (CDM) containing glucose or galactose as carbon source (20 mM), phosphate buffer, trace minerals, vitamins, nucleobases, nitrogen and sulfur sources and amino acids (Ferreira et al., 2013). The nasal synthetic medium (SNM3), described by Krismer et al. (2014), was also used for the analysis of extracellular metabolites by ^1^H-NMR. However, to ensure a metabolite concentration detectable by ^1^H-NMR, glucose was supplemented at higher concentrations (10 mM). All media used for the NMR studies contained no paramagnetic ions to avoid interference with the spectral quality and allow detection of metabolites.

The spermine NONOate (30–40 mM), that was freshly prepared in 0.01 M NaOH and kept protected from light, was used as a NO donor since its half-life (∼39 min at 37°C) is similar to the *S. aureus* cellular doubling time.

Cultures at an initial OD_600_ of 0.05–0.1 were prepared by addition to fresh medium of pre-cultured cells harvested at the exponential phase (OD_600_ of 1). Cells at an OD_600_ of 0.4, were left untreated or exposed to spermine NONOate (250 μM, Sigma), and growth was monitored hourly.

### RNA-Sequencing Analysis

*Staphylococcus aureus* JE2 cells grown on galactose-CDM to an OD_600_ of 0.4 were treated with 250 μM spermine NONOate (or left untreated, the control sample), and harvested after 3 h. Total RNA was isolated from two biologically independent samples following the previously described procedures (Carvalho et al., 2011). RNA samples were analyzed in an Ion Proton^TM^ Sequencer by the PrimBio company (Primbio Research Institute, Innovation Center, Eagleview, Philadelphia, PA, United States). The transcriptome was analyzed by high-resolution RNA-Seq, and the statistical and functional data analysis was done with the Webserver Pipeline T-Rex (de Jong et al., 2015) and Gene Set Enrichment Analysis for prokaryotes (GSEA-Pro) web tool^2^. RNA-Seq data was deposited at Gene Expression Omnibus (GEO) under the accession number GSE99563. Genes were considered with a significantly modified expression for adjusted *P*-values ≤ 0.05 and fold changes of ≥ 2 or ≤ 0.5.

The RNA-Seq data was confirmed by real-time quantitative reverse transcription–polymerase chain reaction (qRT-PCR). For these experiments, *S. aureus* was grown as described above for the RNA-Seq studies, and the total RNA was extracted from cells with the High Pure RNA Isolation Kit (Roche), and further purified by DNase treatment with the Turbo DNA-free^TM^ Kit (Ambion^®^). The absence of chromosomal DNA in RNA samples was confirmed by PCR using oligonucleotides for the *S. aureus* 16S gene (Supplementary Table S1). RNA concentration and purity was determined by gel electrophoresis and in a NanoDrop 2000c UV-Vis Spectrophotometer (ThermoScientific). Total RNA (∼3 μg) served to synthesize cDNA by means of the Transcriptor High Fidelity cDNA synthesis Kit (Roche). Real-time qRT-PCR reactions containing ∼300 ng cDNA template, 0.5 μM gene-specific oligonucleotides (Supplementary Table S1), H_2_O PCR-grade and LightCycler^®^ 480 CYBR Green Master Mix in 20 μL were carried out in a Roche LightCycler LC480 Instrument following the LightCycler^®^480 SYBR Green I Master Kit manufacturer’s instructions (Roche). The expression ratios of the selected genes were normalized relative to *S. aureus* 16S rRNA reference cDNA, whose expression does not vary under the tested conditions, and calculated by the 2^-ΔΔC^_T_ method (Livak and Schmittgen, 2001). The data are an average of two independent biological samples.

### Quantification of Extracellular Metabolites by ^1^H-NMR

Cells were grown on galactose-CDM, glucose-CDM and in the nasal synthetic medium to an OD_600_ = 0.4 and treated with 250 μM spermine NONOate (or left untreated, the control sample), and harvested 1 and 3 h later. After centrifugation (11800 ×*g* for 2 min) and filtering (0.22 μm Pall filters), the supernatants were stored at -20°C and later used for the ^1^H-NMR studies. Extracellular metabolites were quantified on a Bruker AVANCE II500 MHz spectrometer (Bruker BioSpin GmbH) operated by TOPSPIN software using a 5 mm BBIXYZ high resolution probe head, at 16°C, and standard Bruker pulse programs. Spectra were referenced to the resonance of externally added trimethylsilyl propionate (TSP), designated at 0 ppm.

Resonances were assigned by addition to the supernatants of pure compounds and comparison with data available in the literature (Carvalho et al., 2011). Concentrations were calculated from the areas of the resonances of the ^1^H-NMR spectra considering the TSP resonance area, and using an appropriate resonance saturation correction factor (Carvalho et al., 2011). The metabolites concentration was normalized to the OD_600_, measured for each condition.

### Determination of NADP^+^/NADPH Ratios

#### Extraction Method

Cell cultures (5–10 mL) harvested 3 h after the NO addition were pelleted (11800 ×*g*, 2 min), immediately flash frozen into liquid nitrogen and stored at -20°C for the next-day analyses. For extraction of NADP^+^ and NADPH, the pellets were resuspended in 0.4 N HClO_4_ and 0.4 N KOH, respectively, incubated on ice for 15 min, and heated at 60°C for 7 min. The resulting extracts were cooled on ice for 10 min, and the cells debris were pelleted at 3000 ×*g* for 10 min for the NADP^+^ extraction, and at 10,000 ×*g* for 15 min for NADPH. The extracts were filtered and neutralized by slow addition of NaOH or HCl (2–5 M), and kept on ice (based on Lilius et al., 1979).

#### Cycling Reaction

NADPH was enzymatically converted to NADP^+^ following the procedure described by Vaseghi et al. (1999), and the NADP^+^ concentration was assayed by the enzymatic cycling method (Vaseghi et al., 1999) with minor modifications. Briefly, a reaction mixture containing 0.1 M bicine, 0.1 M nicotinamide, 1 mM PES (phenazine ethosulfate), 4.2 mM MgSO_4_, 12.7 mM G6P (glucose 6-phosphate) and dinucleotide extracts in a final volume of 250 μL was prepared. The reaction was initiated by addition of glucose 6-phosphate dehydrogenase (G6PDH) and thiazoylyl blue tetrazolium bromide to final concentrations of 2.8 U and 0.25 mM, respectively. Water was used as blank and standard curves were obtained with β-NADP^+^ (Sigma). Reactions were monitored at 570 nm, for 30 min, using a Multiskan GO Microplate Spectrophotometer (Thermo Fisher Scientific).

### Macrophage Assays

Murine macrophages J774A.1 (LGC Promochem) were cultured on 24-well plates, containing Dulbecco’s modified Eagle medium (31966 – DMEM, Gibco) supplemented with 4.5 g L^-1^ glucose, 110 mg L^-1^ sodium pyruvate, 862 mg L^-1^ glutamine, 15 mg L^-1^ phenol red, 10% fetal bovine serum, and 50 μg mL^-1^ streptomycin, and incubated in a 5% CO_2_ containing atmosphere (BINDER CO_2_ incubator, United States) at 37°C, for 24 h. Prior to infection, macrophages were activated by incubation (5 h) with IFN-γ (1 μg mL^-1^) and LPS (5 μg mL^-1^), both from Sigma.

*Staphylococcus aureus* wild type and *alsS* mutant were grown aerobically in LB to an OD_600_ of 0.4–0.5, washed three times with PBS, and resuspended in antibiotic-free DMEM medium (Gibco) to an initial bacterial concentration of about 1 × 10^7^ CFU mL^-1^. Bacteria were used to infect macrophages at a MOI of ∼5 and, after 30 min the extracellular bacteria were eliminated by a 10 min treatment with DMEM supplemented with 50 μg mL^-1^ gentamicin. Macrophages were washed with PBS and fresh DMEM (1 mL) was added, followed by a 5 h incubation. When required, 800 μM L-NMMA (Sigma) was added to DMEM to promote inhibition of the murine macrophage inducible NO synthase (iNOS). Macrophages were lysed with 2% saponin, and the number of intracellular bacteria determined by CFU counting.

The NO accumulation in the supernatants of the macrophages cell cultures, which reflects the iNOS activity, was assessed by measuring nitrite by the Griess method, which uses sodium nitrite (Merck) as standard.

### Determination of Minimal Inhibitory Concentration

Minimal inhibitory concentrations (MICs) were carried out by the broth tube dilution test. Briefly, 96-well plates containing TSB were inoculated to an initial OD_600_ of 0.01 with exponentially TSB-grown cells of *S. aureus* wild type and *alsS* mutant. Beta-lactam antibiotics methicillin and oxacillin were added to bacterial cell suspensions in a concentration range of 2–32 μg mL^-1^. Plates were incubated at 37°C and 90 rpm, for 18 h. The antibiotic concentration of the first well of the series with no sign of visible growth was considered the MIC value.

### Statistics

Statistical analyses were performed using GraphPad Prism (GraphPad software version 5.01, San Diego, CA, United States). Results were compared using two tailed unpaired Student’s *t*-tests with a confidence interval of 95%. Growths, metabolite quantification, macrophage assays, and MICs were performed at least in triplicate (at least three biological independent experiments). All data is presented as mean ± standard error of the mean (SEM).

## Results

### Analysis of the End Products Profile of Galactose-Grown *S. aureus* NO-Stressed Cells

The hexoses, glucose and galactose are two main glycolytic carbon sources that *S. aureus* has available in the nasal cavity where the NO levels are high. In this work, we have determined the resistance to nitrosative stress of *S. aureus* grown on galactose and observed that, similar to glucose, spermine-NONOate (250 μM) decreased the growth rate by ∼2-times, and that higher concentrations of spermine-NONOate (500 μM) caused no further decrease (Supplementary Figure S1). These results show that the resistance of *S. aureus* to high concentrations of NO is similar for cells grown on galactose or on glucose.

**FIGURE 2:**
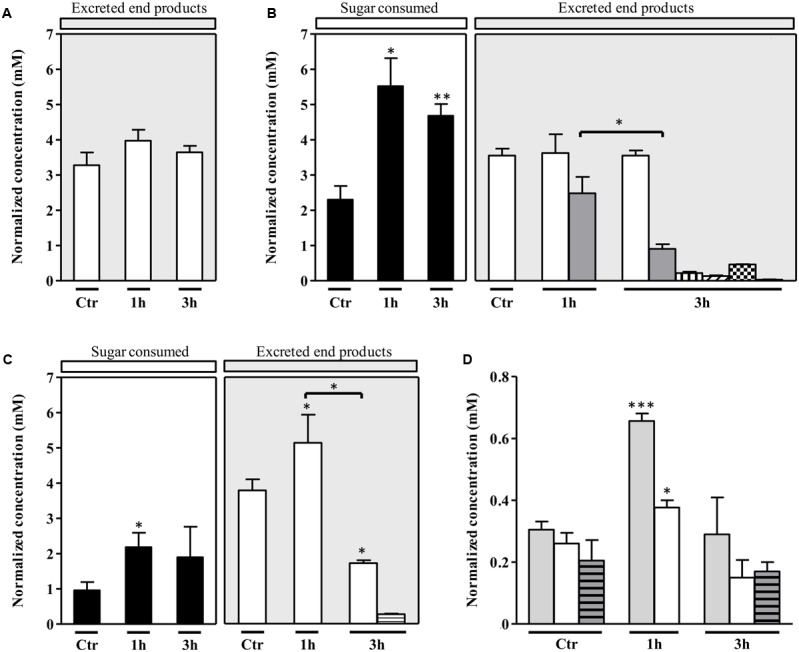
Extracellular metabolite profile of *S. aureus* exposed to NO. Substrate consumed and major end products accumulated by *S. aureus* grown in CDM and **(A)** with no glycolytic carbon source, **(B)** 20 mM glucose, **(C)** 20 mM galactose, in the absence of NO (control, Ctr) and 1 and 3 h after the addition of NO. Color code of bars of graphs A, B, and C: black, sugar consumed; white, acetate; gray, lactate; horizontally striped, formate; vertically striped, pyruvate; right transversally striped, α-acetolactate; black squares, acetoin and left transversally striped, 2,3-butanediol. **(D)** Citrate consumption by *S. aureus* grown with no glycolytic carbon source (light gray bars), on glucose (white bars) and on galactose (gray horizontally striped bars), in the absence of NO (control, Ctr) and treated for 1 and 3 h with NO. The concentrations represented in Y-axis are normalized to OD_600_. Error bars represent mean ± SEM (*n* = 3). Asterisks represent statistically significant data relative to the control; ^∗^*P* < 0.05, ^∗∗^*P* < 0.01, and ^∗∗∗^*P* < 0.0001.

To elucidate how *S. aureus* sustains the nitrosative stress, the metabolic state of *S. aureus* grown on galactose and exposed to NO was studied by ^1^H-NMR quantification of the glycolytic carbon source consumption, the citrate uptake from the growth medium to feed the TCA cycle, and the excreted end products, namely those formed downstream of the pyruvate node (**Figure 1**). For comparison purposes, cells grown on glucose were also analyzed. Thus, supernatants were collected from cells cultured without glycolytic substrate, with either glucose or galactose, treated with 250 μM spermine NONOate, and analyzed 1 and 3 h after the addition of the NO compound (**Figures 2A–C**). Control samples of untreated cells were collected at the exponential phase as their metabolism remained essentially unchanged due to a *quasi* steady-state observed at this growth stage (data not shown).

In the absence of a glycolytic substrate, the ^1^H-NMR profile indicated that *S. aureus* excreted almost exclusively acetate, in amounts that did not vary significantly upon exposure to NO (**Figure 2A**).

In *S. aureus* cells cultured on glucose and stressed with NO, the consumption of glucose was more accentuated at the earlier times of NO treatment (after 1 h) (**Figure 2B**). Moreover, short exposure of cells to NO induced a considerable accumulation of L-lactate (**Figure 2B**), together with very small quantities of ethanol (Supplementary Table S2). Between 1 and 3 h after the NO pulse, a small increase of the lactate concentration in the extracellular medium (from 1.6 ± 0.4 mM to 2.4 ± 0.5 mM) (Supplementary Table S2), and a decrease of the normalized lactate concentration was observed (**Figure 2B**). These alterations may result from the reduction of the lactate dehydrogenase LDH activity and/or higher consumption of lactate by lactate-quinone oxidoreductase LQO (**Figure 1**) in NO treated cells, as it was also observed by Spahich et al. (2016).

The spectra of supernatants collected after 3 h of NO stress showed peaks for pyruvate, α-acetolactate (generated from the condensation of two molecules of pyruvate), acetoin and 2, 3-butanediol (**Figure 2B**), the last three being products of ALS (encoded by *alsS*), α-acetolactate decarboxylase ALDC (encoded by *budA*) and acetoin reductase AR (encoded by *butA*) activity, respectively (**Figure 1**). Interestingly, such a pattern was not observed in cells that were not subjected to NO stress (**Figure 2B**). In addition, the acetate accumulated in untreated cells, and the end products measured at 1 and 3 h after the NO addition do not account for all the glucose consumed.

The citrate consumption from the glucose-containing medium was also evaluated and the results showed that citrate utilization initially increased after the NO pulse, followed by a decrease after 3 h of stress (**Figure 2D**). This result may be interpreted considering that at the onset of the NO stress, the carbon flow in the TCA cycle is higher and the pyruvate, resulting from glucose catabolism, is not only converted to end products but is also diverted to the TCA cycle (**Figures 1**, **2**).

On galactose, unstressed *S. aureus* cells excreted mainly acetate (**Figure 2C**) and minor amounts of ethanol (Supplementary Table S2). In the presence of NO, cells doubled the galactose consumption and excreted higher amounts of acetate (**Figure 2C**). For longer times of exposure to NO, the galactose consumption did not vary significantly and a meaningful (*P*-value, 0.0130) decrease of the acetate produced occurred together with a slight accumulation of formate (**Figure 2C**). In contrast to what was observed on glucose, cells grown on galactose did not accumulate pyruvate, α-acetolactate, acetoin, and 2,3-butanediol (**Figure 2C**). Moreover, no production of lactate was detected, which is in agreement with the requirement of glucose for the activation of *lctE* (encoding LDH) (Crooke et al., 2013).

Also on galactose, cells prolonged exposed to NO showed no major alteration of the total extracellular concentration of acetate [2.7 ± 0.6 mM (1 h) and 2.7 ± 0.8 mM (3 h)] (Supplementary Table S2) and of galactose consumed (**Figure 2C**), which suggests a metabolic constraint at the level of phosphotransacetylase (PTA)/acetate kinase (ACK) and/or acetate consumption. This impairment may lead either to the conversion of pyruvate into α-acetolactate or to an increase of the TCA cycle flux (Sadykov et al., 2013) (**Figure 1**). Cells grown on galactose in the absence of stress or NO-treated for 3 h showed similar consumption of extracellular citrate (**Figure 2D**), which reinforces the hypothesis toward the formation of α-acetolactate through ALS (**Figure 1**). After 1 h of the NO pulse, cells accumulated citrate (0.11 ± 0.02, concentration normalized to OD_600_), indicating a reduction of the TCA cycle activity. Additionally, *S. aureus* cells submitted to NO for 1 h contained an extracellular acetate content higher than that expected on the basis of the galactose consumed (**Figure 2C**). This result may be due to the utilization of non-glycolytic/gluconeogenic carbon sources such as amino acids (**Figure 1**). We also observed that after 3 h of the NO pulse, the extracellular content of end products (acetate and formate) did not account for all the galactose consumed.

Overall, our results show that during prolonged exposure to NO, *S. aureus* grown on galactose exhibits an unprecedented metabolic behavior when compared with that previously reported for cells grown on glucose and long exposed to NO.

### RNA-Seq of *S. aureus* Grown on Galactose and Exposed to NO

To further elucidate the metabolic behavior of *S. aureus* on galactose during prolonged NO exposure, a RNA-Seq analysis was done using RNA extracted from cells grown on galactose and submitted to 250 μM spermine NONOate for 3 h. Following data acquisition, the RNA-Seq results were analyzed using the T-Rex pipeline web tool http://genome2d.molgenrug.nl/index.php/rnaseq-expression-analysis (de Jong et al., 2015) (Supplementary Table S3), and the data validated by quantitative real-time RT-PCR (Supplementary Table S4).

Exposure of *S. aureus* to NO altered significantly the expression of approximately 514 genes (fold-differences ≥ 2 and ≤ 0.5 with adjusted *P*-values ≤ 0.05), which represents approximately 19% of the whole genome. Of these, 213 genes were upregulated (8%) and 301 genes were downregulated (11.4%) (Supplementary Table S3 and Figure S2).

To further interpret the data, a Gene Set Enrichment Analysis (GSEA) of the modified genes was performed, using the pipeline web tool http://pepper.molgenrug.nl/index.php/gsea-pro (GSEA-Pro, MolGen, RUG). This analysis showed that among the upregulated genes involved in central carbon metabolism, the majority are related with metabolic processes and catalytic activities, such as the amino acid and pentose phosphate metabolisms (Supplementary Table S5). The more over-represented classes include genes involved in glycine, serine and threonine metabolism, intracellular trafficking, secretion and vesicular transport and iron transport, storage and homeostasis (Supplementary Table S5).

Amongst the most induced genes belonging to the amino acid transport and metabolism categories were those involved in the synthesis of the gluconeogenic amino acid threonine (Halsey et al., 2017) (*thrA*, *asd*, *dhoM*, *thrB*, *thrC*) that were strongly upregulated (∼10-fold). The genes for the synthesis of other gluconeogenic amino acids, such as glycine (*gLY1*), glutamate (*gltB*, *gltD*) and the BCAAs valine and leucine (*ilvN*) (Supplementary Table S3) were also upregulated, although to a lesser extent. In contrast, NO caused the downregulation of genes involved in respiration, namely those encoding the ATP synthesis, hydrolysis coupled proton transporters, the respiratory electron transport chain and cytochromes (Supplementary Table S3). Other pathways were also altered: (i) the TCA cycle, as judged by the upregulation of the citrate synthase *gltA* (2.2-fold) gene; and (ii) the PPP, by the induction of the genes glucose 6-phosphate 1-dehydrogenase *zwf* (2.1-fold), gluconate kinase *gntK* (2.2-fold) and transaldolase *taI* (2.9-fold). Overall, these results suggest that cells of *S. aureus* using galactose and exposed to NO rely on the synthesis of gluconeogenic amino acids and gluconeogenic processes to support growth on the slow-metabolizing galactose.

D-glutamate, generated from L-glutamate by the activity of glutamate racemase (*murI*), and glycine are cell wall constituents, and BCAAs are precursors of membrane branched-chain fatty acids (BCFA). We observed that genes coding for cell wall, membrane and envelope biogenesis related proteins were significantly induced (Supplementary Table S5). Of note was the induction of genes involved in the synthesis of capsule *cap5* (2.1-fold), in adherence and nasopharyngeal colonization, such as *sasF* (3.9-fold), *sdrC* (12.3-fold), and *sdrD* (2.5-fold) (Supplementary Table S3) (Edwards et al., 2012). The modified transcription was also observed for the genes involved in the fatty acid metabolism, namely the fatty acid degradation genes *fadXEDBA* (23-fold), the fatty acid biosynthesis genes *fabHF* (4-fold) and *fabDG* (2.5-fold) (Supplementary Table S3). Altogether, the data suggest that cells of *S. aureus* using galactose and exposed to NO change their membrane composition to adapt to the new conditions.

The expression of several iron-regulated genes was induced, including that of *ftn* (14.3-fold) and *feoA* (4.1-fold) that code for a non-heme ferritin family protein and the ferrous iron transport protein A, respectively, and that of the siderophore biosynthesis gene *sbnA* (2.2-fold) (Supplementary Table S3). On the contrary, the methionine biosynthesis genes *metCFE* exhibited down-regulation of approximately 2.5-fold (Supplementary Table S3). Interestingly, the iron-regulated genes and methionine biosynthesis genes that were differentially expressed in *S. aureus*, when grown on galactose and under prolonged NO stress, exhibited trends similar to those observed in the transcriptome of *S. aureus* done on samples collected from the anterior nares (Chaves-Moreno et al., 2016).

Several transcriptional regulators had their expression modified, namely TetR, MerR, ArgR, GntR, and DeoR (Supplementary Table S3). The *cidR* gene was also upregulated by NO, *cidR* encodes a LysR-type family regulator that controls the *cidABC* and *alsS/budA* operons (Supplementary Table S3). In accordance, the upregulation of *alsS/budA* was observed and confirmed by quantitative real-time RT-PCR (Supplementary Tables S3, S4). Interestingly, our previous work also showed that *S. aureus* cells grown on glucose and exposed to GSNO had high induction of *alsS* (∼16-fold) (Nobre and Saraiva, 2013), indicating that ALS confers resistance to NO independently of the sugar source.

Genes involved in the pyruvate metabolism were affected such as the lactate dehydrogenase *lctE* gene that had its transcription decreased, a pattern that is similar to that observed in cells grown on glucose in the presence of GSNO (Nobre and Saraiva, 2013).

Also downregulated were the genes coding for the PFL (∼3-fold) and *eutD* encoding phosphotransacetylase (PTA) (∼2-fold), which is the first enzyme of the acetate pathway (Supplementary Table S3 and **Figure 1**) (Halsey et al., 2017).

Altogether, the modifications induced by prolonged NO exposure on the genes encoding proteins acting downstream of the pyruvate node were consistent with the metabolic profile determined by the ^1^H-NMR experiments (**Figure 2C**).

The growth of *S. aureus* on galactose caused high induction of the *cap5A*, *sdrC*, *thrA*, and *fadE* genes that are linked to cell wall components, amino acid and fatty acid metabolism. This behavior contrasts with what was observed for cells grown on glucose (Supplementary Table S4), therefore, suggesting a specific galactose-linked response. The *alsS/budA* operon was upregulated both on galactose- and glucose-grown *S. aureus* cells subjected to prolonged NO stress (Supplementary Table S4), indicating that *alsS* is important independently of the glycolytic carbon source utilized. Moreover, this response is related to a prolonged exposure to NO, as galactose-grown cells exposed to 1 h of NO stress caused the downregulation of the *alsS* and *budA* genes (0.4 ± 0.0 and 0.6 ± 0.2, respectively), while the expression of these genes did not vary significantly in glucose-grown cells (data not shown).

Taken together, the RNA-Seq and qRT-PCR data indicate that when *S. aureus* uses galactose and has prolonged exposure to NO stress, *alsS* is highly upregulated as well as the genes for the biosynthesis of gluconeogenic amino acids, such as threonine. Moreover, the gluconeogenic processes and the biosynthesis of gluconeogenic amino acids are activated.

### Amino Acid Metabolism of *S. aureus* under NO Stress

**FIGURE 3:**
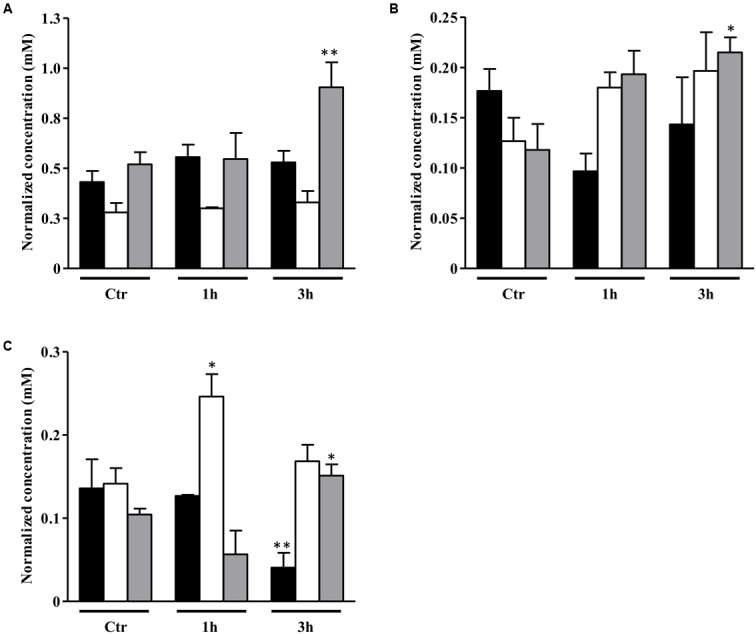
Uptake of amino acids by *S. aureus* cells exposed to NO. Uptake of alanine **(A)**, isoleucine **(B),** and valine **(C)** by *S. aureus* grown in CDM with no glycolytic carbon source (black bar), on glucose (white bar) or on galactose (gray bar), in the absence (Ctr), and treated with NO, for 1 and 3 h. Error bars represent mean ± SEM (*n* = 3). Asterisks represent statistically significant data relative to the control; ^∗^*P* < 0.05 and ^∗∗^*P* < 0.01.

As mentioned above, the genes involved in the class of amino acid transport and metabolism were highly induced in NO treated *S. aureus* galactose-grown cells (Supplementary Table S3). Therefore, we performed the quantification by ^1^H-NMR of the extracellular amino acids, and the results showed that under these conditions cells had a considerable consumption of alanine, and a slightly higher consumption of BCAAs, such as isoleucine and valine (**Figure 3**), but suffered depletion of glycine, glutamate and threonine (Supplementary Figure S3). It was also seen higher accumulation of isovaleric acid and 2-ketobutyric acid (Supplementary Figure S4), which are products of leucine and valine degradation, respectively, most possibly due to an increased requirement of acetyl-CoA.

**FIGURE 4:**
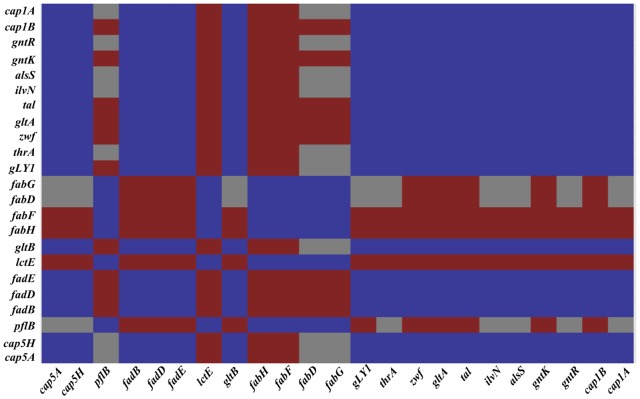
A correlation matrix of selected genes. Correlation of selected genes of classes differently expressed on *S. aureus* cells grown on galactose and under NO stress as determined by the T-Rex pipeline (de Jong et al., 2015). Genes with significant correlation are colored dark blue, genes showing good anti-correlation are colored red and uncorrelated genes are colored gray. SAUSA300_0152 (*cap5A*), SAUSA300_0159 (*cap5H*), SAUSA300_2597 (*cap1B*), SAUSA300_2598 (*cap1A*), SAUSA300_0220 (*pflB*), SAUSA300_0226-28 (*fadBDE*), SAUSA300_0235 (*lctE*), SAUSA300_0885-6 (*fabHF*), SAUSA300_1123-4 (*fadDG*), SAUSA300_1641 (*gltA*), SAUSA300_1454 (*zwf*), SAUSA300_1725 (*taI*), SAUSA300_2443 (*gntK*), SAUSA300_2166 (*alsS*), SAUSA300_2444 (*gntR*), SAUSA300_2008 (*ilvN*), SAUSA300_1225 (*thrA*), SAUSA300_1214 (*gLY1*), and SAUSA300_0445 (*gltB*).

**FIGURE 5:**
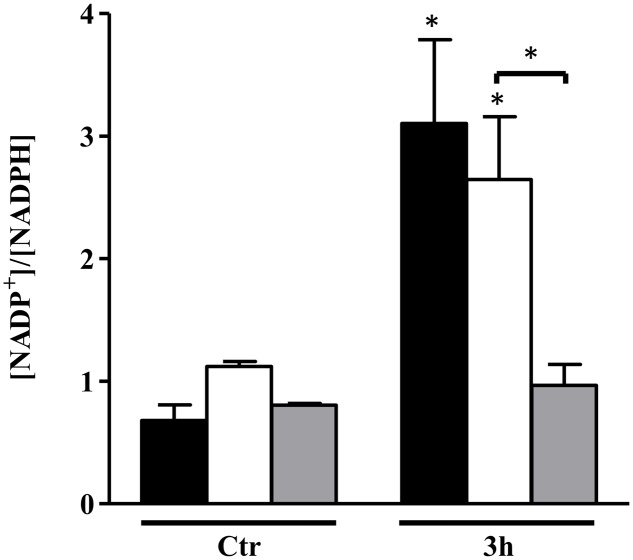
NADP^+^/NADPH ratio of *S. aureus* NO-stressed cells. *S. aureus* was grown in CDM with no glycolytic carbon source (black bars), on glucose (white bars), and on galactose (gray bars) in the absence (Ctr) and under NO stress for 3 h. Error bars represent mean ± SEM (*n* = 3). Asterisks represent statistically significant data relative to the control; ^∗^*P* < 0.05.

Amino acid biosynthesis requires the cofactor NADPH, whose major intracellular source is the PPP that is also used for the production of fatty acids and nucleic acids. A matrix analysis of the RNA-Seq data indicated a high correlation among the PPP genes (*zwf* SAUSA300_1454, *taI* SAUSA300_1725, *gntK* SAUSA300_2443) and amino acid biosynthesis genes (*gntR* SAUSA300_2444, *ilvN* SAUSA300_2008, *thrA* SAUSA300_1225, *gLY1* SAUSA300_1214, *gltB* SAUSA300_0445) (**Figure 4**). Since the reactions of the PPP are dependent on NADP^+^, the NADP^+^/NADPH ratio was determined. Interestingly, following 3 h of NO pulse, cells grown on galactose exhibited a significantly lower NADP^+^/NADPH ratio when compared with cells using glucose (**Figure 5**), indicating that the utilization of galactose increases the metabolic activity of the PPP. A similar behavior was reported for *S. aureus* exposed to oxidative stress, as these cells had higher activity of the PPP and elevated NADPH concentrations (Deng et al., 2014).

### ALS in *S. aureus* Protects against NO Stress

**FIGURE 6:**
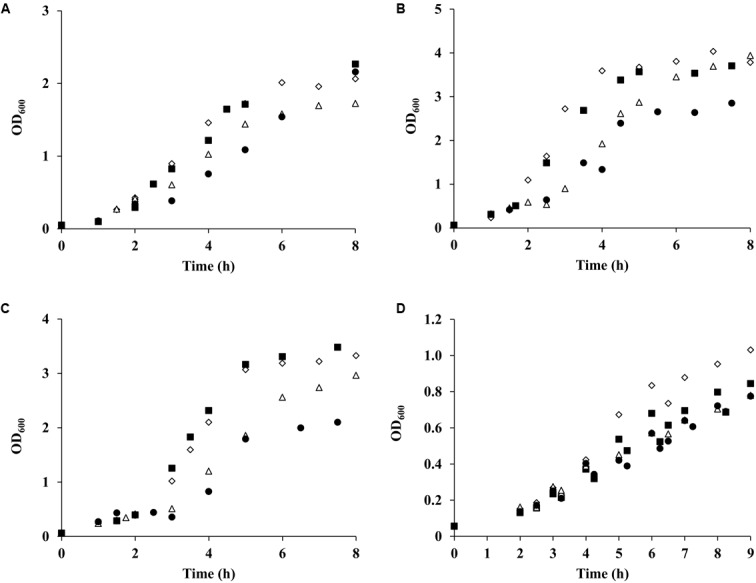
Growth of *S. aureus* wild type and *alsS* mutant exposed to NO. *S. aureus* wild type (♢,Δ) and *alsS* mutant (■,●) were cultured in CDM **(A)**, supplemented with 20 mM glucose **(B)** or with 20 mM galactose **(C),** and in nasal-like synthetic medium containing 10 mM glucose **(D)**. Cells were grown under aerobic conditions in the presence (Δ,●) or absence (♢,■) of NO, which was added at OD_600_ of 0.4. Points represent the average of at least four independent assays.

The metabolic profile of *S. aureus* suggests that prolonged exposure to NO of cells grown on galactose leads to an increase of the ALS activity. Consistent with these results, we observed higher expression of the *alsS* gene and of the genes involved in BCAAs biosynthesis whose precursor is alpha-acetolactate, the product of the ALS activity. Thus, we speculated that ALS was most possible one of the enzymes used to replenish the need for amino acids of cells grown on galactose and under extensive NO stress. To test this hypothesis, a *S. aureus alsS* mutant was studied regarding the growth behavior and metabolic response of stressed galactose-grown cells. For comparison purposes, cells cultured on glucose were also analyzed. The *alsS* mutant grown on either sugar and treated with NO showed a decrease of the final biomass relative to the parental strain (1.5-fold), which was not observed in the absence of a glycolytic substrate (**Figures 6A–C**). However, in all cases, the growth rates were not significantly affected (**Figures 6A–C**), thus suggesting that when using glucose or galactose the strain lacking *alsS* enters earlier into the post-exponential phase (**Figures 6B,C**).

**FIGURE 7:**
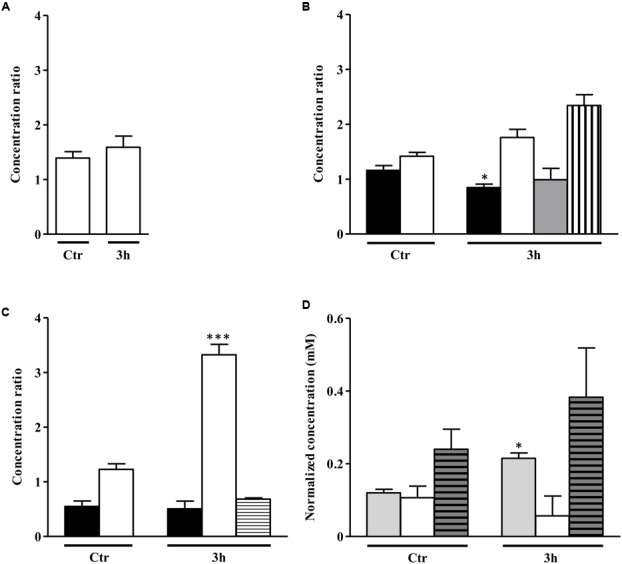
Metabolite profile of *S. aureus alsS* mutant upon exposure to NO. Metabolites evaluated in *S. aureus* grown in the absence of a glycolytic carbon source **(A)** and in media containing 20 mM glucose **(B)** or 20 mM galactose **(C)**, which remained untreated (control, Ctr) or were exposed to NO, for 3 h. Color code of bars of graphs A, B, and C: white, acetate; black, sugar consumed; gray, lactate; horizontally striped, formate; vertically striped, pyruvate. In **(D)** is depicted the citrate accumulation in cells grown with no glycolytic carbon source (light gray bar), on glucose (white bar) and on galactose (gray horizontally striped bar) untreated (control, Ctr) and exposed to NO for 3 h. For each end product, the ratio between the concentrations in the *alsS* mutant divided by that of the wild type cells is represented. Error bars represent mean ± SEM (*n* = 3). Asterisks represent statistically significant data relative to the control; ^∗^*P* < 0.05 and ^∗∗∗^*P* < 0.0001.

Concerning the end products profile, the *S. aureus alsS* mutant grown on a glycolytic carbon source-free medium had only a slight increase of the acetate content relative to that of the wild type independently of the NO treatment (**Figure 7A**).

When cultured on glucose, unexposed *alsS* mutant cells had slightly higher content of acetate than the wild type, and presented minor amounts of lactate (**Figure 7B**). The end products concentration in the *alsS* mutant cells fully accounted for all the glucose consumed. In the absence of NO, the *alsS* mutant produced approximately 5 mM/OD_600_ of acetate and lactate from the consumption of 2.5 mM/OD_600_ of glucose. After 3 h of NO stress, the *alsS* mutant contained approximately two-times more acetate and pyruvate than the wild type, while no accumulation of α-acetolactate, acetoin and 2,3-butanediol was observed, indicating that α-acetolactate is produced by the activity of ALS enzyme (**Figure 7B**). Moreover, the concentration of the end products accounted all the glucose consumed (∼6.3 mM/OD_600_ of acetate, 1 mM/OD_600_ of lactate, and 0.5 mM/OD_600_ of pyruvate were excreted when 3.9 mM/OD_600_ of glucose was consumed). This result suggested that the TCA cycle in the *alsS* mutant is not active, at least to a great extent, which is in agreement with the accumulation of citrate in the extracellular medium rather than consumption (**Figure 7D**). When compared with the wild type, the 3 h NO-treated glucose-grown *alsS* mutant cells accumulated extracellularly approximately the double amount of acidic end products (**Figure 7B**). This data suggest that under these conditions the mutant had a lower intracellular pH level (Thomas et al., 2014), which is consistent with a premature entering into the post-exponential phase (**Figure 6B**).

On galactose, inactivation of *alsS* generated cells that in the NO-free environment contained similar levels of acetate, but consumed two-times less galactose than the wild type (**Figure 7C**). Furthermore, the *alsS* mutant had an increased amount of acetate and formate end products (4.5 mM/OD_600_), than those expected from the consumption of 0.5 mM/OD_600_ of galactose, which contrasts with the total carbon recovery observed on glucose. When exposed to NO, the *alsS* mutant had a threefold higher accumulation of acetate and a slightly lower amount of formate than the parental strain (**Figure 7C**). These results indicate that a strain inactivated in *alsS* and exposed to NO does not produce α-acetolactate and its derivatives, but instead leads to a higher accumulation of acidic end products (**Figure 7C**).

When treated with NO, *S. aureus* cells grown on galactose contained no acetoin and 2,3-butanediol. Moreover, the mutation of *als*S increased the acetate content. Therefore, the derived compounds of α-acetolactate present in the wild type strain are most probably BCAAs as α-acetolactate is also a known precursor of the BCAAs synthesis (**Figure 1**) (Goupil-Feuillerat et al., 1997).

Hence, we conclude that under NO stress, the ALS activity plays an important role by preventing extracellular acetate accumulation through the production of α-acetolactate for the biosynthesis of BCAAs, which counteracts the pH stress imposed by the higher content of acetate.

### The Effect of the ALS Inactivation in *S. aureus* Grown on a Nasal-Like Synthetic Medium

Previously, Krismer et al. (2014) described a nasal synthetic medium that was considered similar to the *in vivo* environment experienced by *S. aureus* inside the human nose. Similar to our transcriptome data, the global gene expression analysis of *S. aureus* grown on the nasal-like medium showed a significant upregulation of amino acid biosynthesis genes of glutamate, valine and leucine, and iron transporters (Krismer et al., 2014). Therefore, metabolic studies on cells grown on the nasal-like synthetic medium were also done. To note that when compared with the CDM, the nasal-like synthetic medium contains lower amount of amino acids (∼2-fold lower concentration), and lacks several amino acids, namely, aspartate, methionine, *trans*-hydroxyproline, and isoleucine. Furthermore, the medium contains fumaric, maleic, pyruvic and succinic acids and urea, which are absent from the CDM medium. Also, the citric acid and glucose concentrations are lower by 50- and 2-fold, respectively, and no folic acid is present.

**FIGURE 8:**
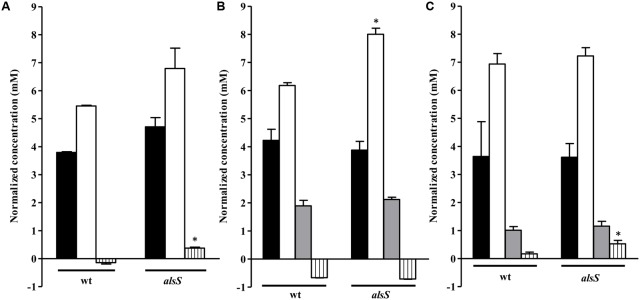
Metabolite profile of the *alsS* mutant grown on nasal synthetic medium exposed to NO. Cells of wild type (wt) and *alsS* mutant grown in nasal synthetic medium untreated **(A)** and exposed to NO for 1 h **(B)** and 3 h **(C)** were analyzed. Black bar, glucose consumed; white bar, acetate accumulated; gray bar, lactate accumulated; vertically striped bars, pyruvate consumed (below 0) or accumulated (above 0). Error bars represent mean ± SEM (*n* = 3). Asterisks represent statistically significant data relative to the wild type; ^∗^*P* < 0.05.

**FIGURE 9:**
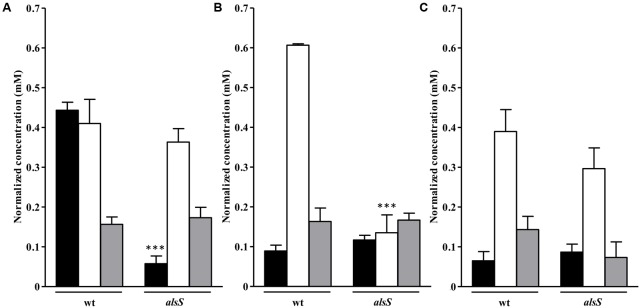
Uptake of amino acids and succinate by the *S. aureus alsS* mutant grown in nasal synthetic medium and subjected to NO. Uptake of valine (black bar), alanine (white bar), and succinate (gray bar) by wild type (wt) and *alsS* mutant strains, untreated **(A)** and exposed to NO for 1 h **(B)** and 3 h **(C)**. Error bars represent mean ± SEM (*n* = 3). Asterisks represent statistically significant data relative to the wild type; ^∗∗∗^*P* < 0.0001.

We observed that *S. aureus* wild type grown on the nasal-like synthetic medium accumulated high levels of acetate, which did not account for all the glucose exhausted from the extracellular medium, and consumed small amounts of pyruvate from the extracellular medium (**Figure 8A**). After 1 h of NO stress, the wild type cells exhibited levels of glucose utilization, similar to those of unstressed cells, and L-lactate accumulation. However, cells accumulated more acetate, had increased consumption of alanine, and no pyruvate was detected (**Figures 8B**, **9B**). Upon prolonged exposure to NO, the lactate levels decreased and pyruvate accumulated (**Figure 8C**).

Cells mutated in *alsS* showed a 1 mM/OD_600_ increase in the acetate levels and pyruvate was accumulated instead of being consumed, while in the wild type no accumulation of the ALS, ALDC and AR products was observed (**Figure 8A**). Moreover, the *alsS* mutant exhibited reduced viability and a fourfold decrease of the valine consumption (**Figures 6D**, **9A**), suggesting that the ALS activity is required for the consumption of BCAAs, and that α-acetolactate is being utilized for the BCAAs synthesis via ALS (**Figure 1**).

Cells of the *alsS* mutant treated with NO for 1 h consumed less alanine and accumulated more acetate extracellularly (**Figures 8B**, **9B**). Furthermore, for longer times of NO exposure, the amount of pyruvate doubled and the consumption of succinate decreased by approximately two-times (**Figures 8C**, **9C**).

Altogether, the NO-stressed *S. aureus alsS* mutant cells presented a different metabolic pattern on the nasal-like medium, which is most probably due to the quite different medium composition on carbon sources and amino acids relative to CDM.

### ALS Contributes to the Survival of *S. aureus* in Macrophages

Taking into consideration that the *alsS* expression and ALS activity are increased by NO and that inactivation of *alsS* rendered the strains more sensitive to NO (Supplementary Table S3 and **Figures 2**, **6B,C**), we next determined the viability of the *alsS* mutant when infecting murine macrophages J774A.1.

**FIGURE 10:**
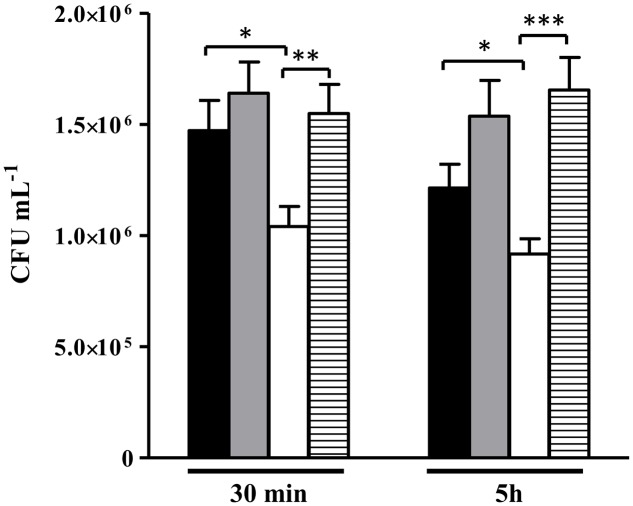
Viability of *S. aureus alsS* mutant contacting with murine macrophages. *S. aureus* wild type (black and gray bar) and *alsS* mutant cells (white and striped bar) were incubated with activated macrophages (black and white bar), and with L-NMMA treated macrophages (gray and striped bar). The number of intracellular *S. aureus* cells were determined at the indicated times. Error bars represent mean ± SEM (*n* = 3). Asterisks represent statistically significant data; ^∗^*P* < 0.05, ^∗∗^*P* < 0.01, and ^∗∗∗^*P* < 0.0001.

Data showed that the *alsS* mutant is more efficiently killed by macrophages than the wild type (**Figure 10**). Similar assays were performed with macrophages previously incubated with the mammalian inhibitor of iNOS (L-NMMA), i.e., in macrophages that do not release NO, which was ascertained by the lowered concentration of extracellular nitrite (Supplementary Figure S5). Treatment with the inhibitor reversed the loss of viability of the *alsS* mutant to levels similar to those of the wild type strain (**Figure 10**). Hence, we concluded that ALS promotes survival from phagocytosis by protecting *S. aureus* from the innate immune NO radical.

### ALS Protects *S. aureus* from Beta-Lactam Antibiotics

The matrix analysis of the RNA-Seq data indicated the occurrence of a high correlation between *alsS* and fatty acid degradation and capsular genes involved in modulation of the cell surface (**Figure 4**). Thus, we questioned whether ALS could play a role in the resistance of the MRSA to beta-lactam antibiotics, which target the cell wall biosynthesis. For this purpose, we determined for the *S. aureus* MRSA wild type and *alsS* mutant the MIC of antibiotics commonly used for the treatment of the pathogen, namely the beta-lactams oxacillin and methicillin. The results showed that inactivation of *alsS* leads to a twofold decrease in the MIC value, i.e., *S. aureus* becomes less resistant to these antibiotics (**Table 1**).

**Table 1:** Minimal inhibitory concentrations of oxacillin and methicillin against *S. aureus.*

*S. aureus*	Oxacillin	Methicillin
	
	MIC (μg mL^-1^)	
Wild type	13	14
*alsS*	6	6

## Discussion

*Staphylococcus aureus* has been reported to tolerate moderate to high levels of NO when using glucose, one of the carbon sources available in the nasal cavity. In this work, we analyzed the behavior of *S. aureus* when aerobically grown on galactose, another abundant hexose on the nasal cavity, and exposed to NO, and compared the results with cells cultured on glucose and on a nasal-like medium.

*Staphylococcus aureus* cultured on galactose exhibited growth rate and biomass yield similar to that observed on glucose, which indicates that the slow-metabolizing galactose also sustains replication of the pathogen under NO stress. However, the metabolism underpinning growth proved to be significantly different. For example, 1 h after the NO stress pulse, cells grown on galactose excreted more acetate than on glucose. Moreover, the increase of acetate excretion and accumulation of extracellular citrate, rather than consumption, indicates a decreased flux of the TCA cycle and respiration as these pathways rely on NO-sensitive enzymes, such as the iron-sulfur containing aconitase and cytochrome oxidase (McCollister et al., 2011; Richardson et al., 2011).

The higher increase of acetate production detected for galactose-grown cells exposed to NO for 1 h provides *S. aureus* higher ATP levels that are particularly relevant for the growth on slowly metabolized carbon sources as was described for other bacteria (Carvalho et al., 2011). However, prolonged NO stress caused a significant decrease of acetate accumulation and the increased consumption of threonine, glycine, glutamate, and BCAAs from the extracellular medium. Additionally, RNA-Seq data showed upregulation of genes required for the synthesis of threonine, glycine, glutamate, BCAAs, and fatty acids degradation, and acting in the first half of the TCA cycle and the PPP. Consistent with these results, cells had higher content of NADPH, a cofactor that boosts anabolic reactions. In *S. typhimurium*, the first enzyme of the PPP glucose 6-phosphate dehydrogenase (G6PDH) was shown to provide reducing equivalents in the form of NADPH and to have an important role in the defense against nitrosative stress by maintaining the cellular redox state, regenerating reduced thiols, and repairing oxidative or nitrosative damage (Lundberg et al., 1999). When using galactose, but not glucose under prolonged NO stress, *S. aureus* showed higher demand of amino acids. Interestingly, similar behavior was also observed for NO-stressed cells grown on the gluconeogenic substrate L-lactate (Spahich et al., 2016).

When *S. aureus* grows on glucose, the exposure to NO does not alter the amount of excreted acetate but causes the increase of the lactate levels, which decrease under prolonged stress. Although these results apparently differ from those previously reported for *S. aureus* showing that in cells treated for 4 h with NO there is a strong increase of lactate and acetate is absent, it should be noted that the metabolite production at earlier times was not analyzed and much higher concentrations of NO were used, conditions that completely inhibit the activities of the PDHC and PFL enzymes (Richardson et al., 2008). Moreover, the contrasting effects of NO on *S. aureus* grown on galactose *versus* glucose may be a consequence of the slower glycolysis, and such behavior is expected to also occur when *S. aureus* uses other slow-metabolizing sugars available in its colonization niche.

Transcriptomic analysis revealed that NO induces the expression of the catabolic *alsS*/*budA* and *butA* genes that encode ALS/ALDC and AR proteins. These gene products are generally regarded as involved in the synthesis of non-acidic products acetoin and 2,3-butanediol and regeneration of NAD^+^ (Goupil-Feuillerat et al., 1997; Thomas et al., 2014). Considering that butanediol fermentation is activated when *S. aureus* shifts from aerobic to anaerobic growth (Fuchs et al., 2007), the sensitivity of the *alsS* mutant strain to NO could derive from an indirect consequence of the inhibition of respiration by NO. However, our data do not support this hypothesis, as we did not observe higher expression of the genes that are usually induced when *S. aureus* grows under anaerobic conditions, such as *adhE*, *adh*, *narH*, *narI*, *narJ*, *pfl* and genes encoding glycolytic enzymes (Fuchs et al., 2007). Moreover, the higher induction of *alsS* occurred 3 h after the NO pulse, which is when the NO inhibitory effect of respiration is expected to be less prominent considering the half-life of the spermine NONOate (∼40 min).

**FIGURE 11:**
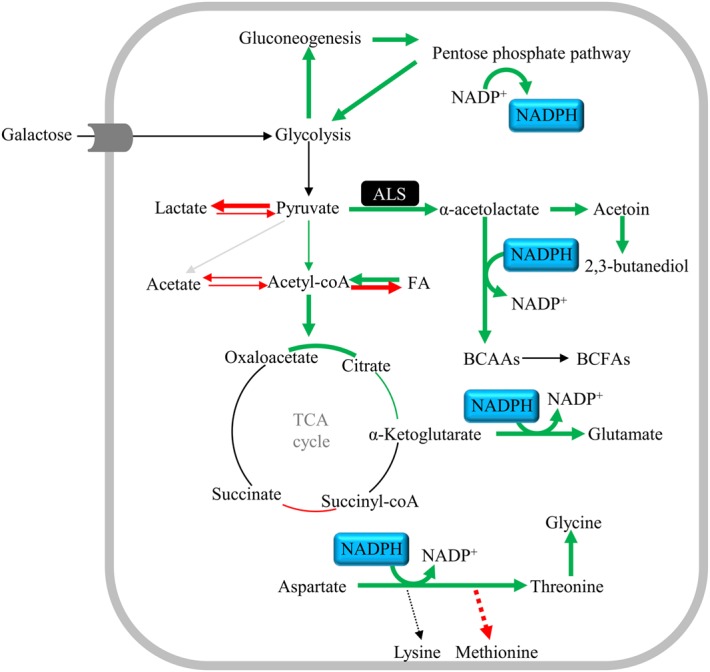
Schematic representation of the central metabolic pathways of *S. aureus* grown on galactose and prolonged exposed to NO. Upregulated and downregulated pathways (cut-off > 2) are denoted with bold green and red arrows, respectively. Narrower green and red arrows indicate significantly altered expression, considering a cut-off between 1.5 and 2. The α-acetolactate synthase (*alsS*) and the NADP^+^, NADPH cofactors are highlighted in black and blue, respectively. Proposed pathways were reconstructed based on genome information (http://www.ncbi.nlm.nih.gov/genomes/lproks.cgi), literature and database surveys (KEGG, MetaCyc). BCAAs, branched-chain amino acids; BCFAs, branched-chain fatty acids; FA, fatty acids.

A previous study reported that the *alsS/budA* operon is activated by the CidR regulator. The gene encoding CidR was also induced in our RNA-Seq study. CidR has been proposed to be activated by a decrease of the intracellular pH, mainly by accumulation of acetic acid (Thomas et al., 2014). In accordance, increased levels of extracellular acidic levels were observed upon exposure of *S. aureus* to NO. Moreover, α-acetolactate, acetoin and 2,3-butanediol, which are the products of the ALS and ALDC/AR activities, were observed on glucose-grown cells under prolonged NO stress. The accumulation of butanediol occurred after a short exposure (20 min) to the NO donor MAHMA NONOate (500 μM) for *S. aureus* COL grown on a glucose-containing medium (Hochgräfe et al., 2008). The absence of α-acetolactate, acetoin and 2,3-butanediol in NO-stressed galactose-grown cells may be due to the higher requirement for BCAAs and the increased levels of NADPH that were observed under these conditions, which shifts the product of ALS activity, α-acetolactate, to the production of BCAAs (**Figure 11**). Interestingly, it was reported for the Gram-positive *Lactococcus lactis* that under BCAAs starvation there is a relief of the allosteric activation of ALDC by BCAAs, and of the allosteric inhibition of the BCAAs synthesis pathway (Goupil-Feuillerat et al., 1997). Moreover, BCAAs serve also as precursors for membrane BCFAs and were shown to be important for pH stress tolerance (Kaiser et al., 2016). Alternatively, the requirement for BCAAs may be related with the inhibition by NO of the dihydroxyacid dehydratase enzyme (coded by *ilvD* and involved in the BCAAs biosynthesis), similarly to what was observed for the dihydroxyacid dehydratase of *Escherichia coli* (Hyduke et al., 2007). In accordance with the requirement of *S. aureus* for BCAAs when growing on galactose under prolonged exposure to NO stress, there was a small but significantly higher consumption of the BCAAs valine and isoleucine. The threefold higher accumulation of acetate in the *alsS* mutant strain suggests that ALS directs the metabolism toward the BCAAs production by fuelling pyruvate resulting from galactose breakdown into α-acetolactate production (**Figure 11**). BCAAs were shown to be important for the virulence of *S. aureus* (Kaiser et al., 2016), i.e., under conditions where NO is generated by the host cells. Furthermore, the *S. aureus* CodY transcriptional regulator, that represses several amino acid biosynthetic pathways when intracellular BCAAs and GTP are available (Majerczyk et al., 2010), has been associated with NO resistance and implicated in virulence (Majerczyk et al., 2010; Grosser et al., 2016). Interestingly, a great number of genes that were transcriptionally modified in our RNA-Seq analysis are regulated by CodY, namely threonine, *ilvBN*, and glutamate and capsule synthesis genes.

Contrary to the wild type, the *S. aureus alsS* mutant cells did not consume citrate but excreted it to the extracellular medium, which indicates impairment of the TCA cycle. Furthermore, the mutant cells accumulated higher levels of acetate, especially on galactose-containing medium, which may explain the lower survival of the mutant strain (**Figure 6C**). Consistent with our data, Thomas et al. (2014) reported that the *S. aureus alsS/budA* mutant had an accrual of extracellular acetate that results in increased cell death linked to ROS accumulation. Moreover, we observed that the valine consumed upon *alsS* inactivation lowered significantly (data not shown), suggesting that ALS may be required for the consumption of valine, an issue that requires future detailed analysis.

The *alsS* mutant had lower survival within macrophages, and inhibition of the mammalian NO synthase lifted the growth impairment. This result points to a NO-related protective role for ALS and is in line with the high expression of *alsS* in samples collected from human nares that contain significant NO levels (Chaves-Moreno et al., 2016).

In the nasal-like medium, the cellular consumption of glucose did not change significantly upon 1 h of NO exposure; however, the pyruvate (present in the medium at high concentrations of 300 μM) was completely utilized and alanine was more consumed. Contrary to what was observed in cells grown in CDM, in the nasal-like medium, pyruvate was consumed instead of glucose to produce lactate for balancing the reducing equivalents (NAD^+^) (**Figures 2B**, **8B**).

In nasal-like medium, *alsS* proved also to have an important role as its inactivation causes growth defects. Additionally, the absence of quantifiable amounts of the products of the ALS activity, namely α-acetolactate, acetoin and 2,3-butanediol, indicate a requirement for BCAAs in the nasal medium, which may be due to the absence of isoleucine and the low levels of leucine and valine present in this medium (Krismer et al., 2014).

## Conclusion

We show how *S. aureus* copes with prolonged NO stress when metabolizing a slow-sugar like galactose, that is one of the main hexoses available in the nasal cavity. The following model is therefore proposed (**Figure 11**): NO promotes the formation of acetyl-CoA, which feeds acetyl molecules into the first half of the TCA cycle, and the synthesis of amino acids (e.g., glutamate, threonine, glycine, BCAAs), which are also boosted by the NADPH produced by the upregulated genes of the PPP. Among the genes products involved in amino acid synthesis, one of the most upregulated is that encoding ALS. Under NO stress, ALS seems to have a dual role by preventing acid stress and promoting the synthesis of BCAAs (**Figure 11**). Furthermore, this study disclosed the role of ALS in the resistance of *S. aureus*, by showing that ALS contributes not only to NO resistance but also to the resistance to antibiotics commonly used against *S. aureus*, such as methicillin and oxacillin.

## Author Contributions

SC undertook aspects of the experimental work, in the interpretation of the data and writing of the manuscript. AJ, TK, and OK designed and interpreted the RNA-seq experiments. LS designed and interpreted all aspects of the experimental work.

## Conflict of Interest Statement

The authors declare that the research was conducted in the absence of any commercial or financial relationships that could be construed as a potential conflict of interest.

## Footnotes

1 http://app1.unmc.edu/fgx/methods.html

2 http://pepper.molgenrug.nl/index.php/gsea-pro

## Abbreviations

ALS: α-acetolactate synthase
BCAAs: branched-chain amino acids
EMP: Embden-Meyerhof-Parnas
L-NMMA: N^G^-monomethyl-L-arginine acetate salt
MRSA: methicillin-resistant *Staphylococcus aureus*
NMR: nuclear magnetic resonance
NO: nitric oxide
PDHC: pyruvate dehydrogenase complex
PFL: pyruvate formate-lyase
PPP: pentose phosphate pathway
RNA-Seq: RNA-Sequencing
TCA: tricarboxylic acid cycle.
