# Supplementary material for: The Staphylococcus aureus α-Acetolactate Synthase ALS Confers Resistance to Nitrosative Stress
Source: Front Microbiol. 2017 Jul 11;8:1273. doi: 10.3389/fmicb.2017.01273 (PMC5504149; doi:10.3389/fmicb.2017.01273)
Supplement: Supplementary file 1 [file Data_Sheet_1.docx]

Supplementary Material

**The *Staphylococcus aureus* α-Acetolactate Synthase ALS Confers Resistance to Nitrosative Stress**

**Sandra M. Carvalho^1^, Anne de Jong^2^, Tomas G. Kloosterman^2^, Oscar P. Kuipers^2^, and Lígia M. Saraiva^1*^**

^1^Instituto de Tecnologia Química e Biológica NOVA, Universidade Nova de Lisboa, Oeiras, Portugal, ^2^Department of Molecular Genetics, Groningen Biomolecular Sciences and Biotechnology Institute, University of Groningen, Groningen, Netherlands

**Running title:** *Staphylococcus aureus ALS protects from NO*

**^*^Correspondence:**

Lígia M. Saraiva

[lst@itqb.unl.pt](mailto:lst@itqb.unl.pt)

**Keywords:** *Staphylococcus aureus*, nitrosative stress, α-acetolactate synthase (ALS), nuclear magnetic resonance (NMR)

**SUPPLEMENTAL TABLES**

**Table S1. Oligonucleotides used in this study**

| **Primers** | **Sequence (from 5’ to 3’)** |
| --- | --- |
| 16S_FW | GCGAAGAACCTTACCAAATC |
| 16S_Rev | CCAACATCTCACGACACG |
| alsS_for | CGTGAATTAGAAAATCATTTCTTC |
| alsS_rev | CGGTTGCATATAATTAGTAATTTC |
| budA_for | GCAGTAAAAATTTCAGGCTTA |
| budA_rev | GCAAAGTGTACATGAAATCC |
| cap5A_for | GTGTCAAAGGACTTAAATGAT |
| cap5A_rev | AGATAAAATTGATACGTTATCCAC |
| sdrC_for | GACGCTGACAACATGAC |
| sdrC_rev | GCGGTATTTACCATTTTCATC |
| aspartate kinase_fw | GCCATCATATCAAGTGGTG |
| aspartate kinase_rev | GATGCGATCTTTATAAAGTGG |
| fadE_fw | CGTGCTAGTAATTTTAATCCTG |
| fadE_rev | TGCTTTACCAATACTCATTGG |
| G6PDH_fw | CTGCGTCATTTCCAATCTGAAG |
| G6PDH_rev | CATACGTTTACCAGTACGAATATAG |
| FBP_fw | GCTATTGCGATGATTCAATTC |
| FBP_rev | CTTCTTCAGGTAGTAATTCTGC |

**Table S2. End products accumulated and glycolytic carbon sources consumed (mM) by *S. aureus* wild type treated with NO**

|  | No glycolytic carbon source | | | Glucose | | | Galactose | | |
| --- | --- | --- | --- | --- | --- | --- | --- | --- | --- |
|  | Ctr | 1h | 3h | Ctr | 1h | 3h | Ctr | 1h | 3h |
| Glc consumed | - | - | - | 3.6±0.9 | 3.4±0.8 | 12.1±0.7 | - | - | - |
| Gal consumed | - | - | - | - | - | - | 1.0±0.2 | 1.1±0.1 | 2.6±0.6 |
| Acetate | 3.1±0.2 | 1.6±0.2 | 3.3±0.1 | 5.3±1.5 | 2.3±0.5 | 9.2±0.2 | 4.0±0.2 | 2.7±0.6 | 2.7±0.8 |
| Lactate | ND^a^ | ND | ND | ND | 1.6±0.4 | 2.4±0.5 | ND | ND | ND |
| Pyruvate | ND | ND | ND | ND | ND | 0.6±0.0 | ND | ND | ND |
| Formate | ND | ND | ND | ND | ND | ND | ND | ND | 0.4±0.1 |
| ɑ-acetolactate | ND | ND | ND | ND | ND | 0.4±0.0 | ND | ND | ND |
| Acetoin | ND | ND | ND | ND | ND | 1.2±0.0 | ND | ND | ND |
| 2,3-butanediol | ND | ND | ND | ND | ND | 0.1±0.0 | ND | ND | ND |
| Ethanol | 0.1±0.0 | ND | ND | 0.2±0.0 | 0.2±0.0 | ND | 0.1±0.0 | 0.1±0.0 | ND |

^a^ND, not detected

**Table S3.** **Genes affected by NO in *S. aureus* grown on galactose**

| **Locus_tag (old)** | **Locus_tag (new)** | **Gene name^a^** | **Adjusted *p*-value** | **Fold** | **Annotation** | **COG category^b^** |
| --- | --- | --- | --- | --- | --- | --- |
| *Upregulated genes* |  |  |  |  |  |  |
| SAUSA300_0054 | SAUSA300_0054 |  | 1.12E-04 | 91.2 | hypothetical protein |  |
| SAUSA300_0179 | SAUSA300_RS00940 | ***fdh*** | 9.86E-67 | 33.2 | formate dehydrogenase | [R] |
| SAUSA300_0228 | SAUSA300_RS01210 | *fadE* | 3.61E-107 | 26.9 | long-chain-fatty-acid--CoA ligase |  |
| SAUSA300_0229 | SAUSA300_RS01215 | ***fadX*** | 7.87E-96 | 23 | hypothetical protein |  |
| SAUSA300_0227 | SAUSA300_RS01205 | *fadD* | 6.76E-98 | 21.5 | glutaryl-CoA dehydrogenase |  |
| SAUSA300_0226 | SAUSA300_RS01200 | ***fadB*** | 2.43E-88 | 17.9 | 3-hydroxyacyl-CoA dehydrogenase |  |
| SAUSA300_0178 | SAUSA300_RS00935 |  | 4.15E-118 | 16.7 | hypothetical protein |  |
| SAUSA300_0225 | SAUSA300_RS01195 | ***fadA*** | 2.04E-92 | 14.3 | acetyl-CoA acetyltransferase |  |
| SAUSA300_1874 | SAUSA300_RS10250 | ***ftn*** | 6.48E-79 | 14.3 | non-heme ferritin | [P][F] |
| SAUSA300_0546 | SAUSA300_RS02915 | *sdrC* | 6.78E-77 | 12.3 | serine-aspartate repeat-containing protein C | [M] |
| SAUSA300_1226 | SAUSA300_RS06645 | ***dhoM*** | 2.39E-38 | 9.9 | homoserine dehydrogenase | [E][R] |
| SAUSA300_2092 | SAUSA300_RS11525 | *dps* | 3.27E-17 | 9.4 | DNA starvation/stationary phase protection protein | [F] |
| SAUSA300_1225 | SAUSA300_RS06640 | ***thrA*** | 1.24E-17 | 8.2 | aspartate kinase |  |
| SAUSA300_2588 | SAUSA300_RS14390 | *secY* | 1.39E-34 | 8 | accessory Sec system protein translocase subunit SecY2 | [P] [U] |
| SAUSA300_1228 | SAUSA300_RS06655 | *thrB* | 2.74E-32 | 7.7 | homoserine kinase | [E] |
| SAUSA300_1227 | SAUSA300_RS06650 | *thrC* | 3.44E-27 | 7.2 | threonine synthase |  |
| SAUSA300_0681 | SAUSA300_RS03655 |  | 6.26E-84 | 7.2 | hypothetical protein |  |
| SAUSA300_1259 | SAUSA300_RS06840 |  | 7.47E-47 | 7.2 | DNA repair protein MucB | [L] |
| SAUSA300_2587 | SAUSA300_RS14385 |  | 3.94E-32 | 5.9 | accessory Sec system protein Asp1 | [U] |
| SAUSA300_2586 | SAUSA300_RS14380 |  | 1.58E-36 | 5.9 | accessory Sec system protein Asp2 | [U][R] |
| SAUSA300_0878 | SAUSA300_RS04735 |  | 1.32E-08 | 5.7 | LysR family transcriptional regulator |  |
| SAUSA300_2585 | SAUSA300_RS14375 |  | 3.61E-30 | 5.7 | accessory Sec system protein Asp3 | [U] |
| SAUSA300_2231 | SAUSA300_RS12320 | *fdhD* | 3.90E-46 | 5.7 | formate dehydrogenase accessory protein FdhD |  |
| SAUSA300_1238 | SAUSA300_RS06720 |  | 1.00E-55 | 5.5 | hypothetical protein |  |
| SAUSA300_1373 | SAUSA300_RS07500 |  | 8.41E-33 | 4.8 | ferredoxin |  |
| SAUSA300_2622 | SAUSA300_RS14570 |  | 1.32E-34 | 4.6 | sulfurtransferase |  |
| SAUSA300_0414 | SAUSA300_RS02220 |  | 2.14E-04 | 4.6 | hypothetical protein |  |
| SAUSA300_2554 | SAUSA300_RS14195 |  | 1.10E-32 | 4.5 | sulfite reductase subunit alpha | [R][H] |
| SAUSA300_0955 | SAUSA300_RS05135 | *atl* | 4.95E-12 | 4.4 | bifunctional autolysin | [M][S] |
| SAUSA300_2475 | SAUSA300_RS13735 |  | 1.03E-29 | 4.4 | acyl-CoA thioester hydrolase | [I] |
| SAUSA300_2589 | SAUSA300_RS14395 |  | 4.73E-22 | 4.3 | serine-rich adhesin for platelets | [P] |
| SAUSA300_2488 | SAUSA300_RS13820 | *feoA* | 1.55E-02 | 4.1 | ferrous iron transporter A |  |
| SAUSA300_2479 | SAUSA300_RS13755 | *cidA* | 4.41E-07 | 4.1 | holin-like protein CidA |  |
| SAUSA300_2253 | SAUSA300_RS12440 | *ssaA* | 6.65E-14 | 4.1 | CHAP domain-containing protein |  |
| SAUSA300_2581 | SAUSA300_RS14355 | ***sasF*** | 5.64E-27 | 3.9 | surface anchored protein |  |
| SAUSA300_0544 | SAUSA300_RS02905 |  | 5.59E-33 | 3.9 | haloacid dehalogenase |  |
| SAUSA300_0413 | SAUSA300_RS02215 |  | 5.06E-07 | 3.9 | hypothetical protein |  |
| SAUSA300_0956 | SAUSA300_RS05140 |  | 4.74E-20 | 3.9 | GNAT family N-acetyltransferase |  |
| SAUSA300_1875 | SAUSA300_RS10260 |  | 8.96E-18 | 3.9 | DNA polymerase III subunit epsilon | [R][L] |
| SAUSA300_2165 | SAUSA300_RS11935 | *budA* | 9.37E-15 | 3.8 | alpha-acetolactate decarboxylase | [Q] |
| SAUSA300_0415 | SAUSA300_RS02225 | *lpl3* | 5.21E-06 | 3.8 | hypothetical protein |  |
| SAUSA300_1068 | SAUSA300_RS05795 |  | 2.42E-04 | 3.8 | hypothetical protein | [R] |
| SAUSA300_2166 | SAUSA300_RS11940 | *alsS* | 6.77E-20 | 3.8 | acetolactate synthase |  |
| SAUSA300_2553 | SAUSA300_RS14190 |  | 4.25E-29 | 3.7 | precorrin-2 dehydrogenase |  |
| SAUSA300_2597 | SAUSA300_RS14440 | *cap1B* | 1.10E-07 | 3.7 | capsular polysaccharide biosynthesis protein Cap1B |  |
| SAUSA300_0416 | SAUSA300_RS02230 |  | 3.04E-06 | 3.6 | hypothetical protein |  |
| SAUSA300_1237 | SAUSA300_RS06710 | *lexA* | 2.57E-16 | 3.6 | LexA repressor | [K][O] |
| SAUSA300_1182 | SAUSA300_RS06390 |  | 1.16E-31 | 3.6 | 2-oxoglutarate ferredoxin oxidoreductase subunit alpha |  |
| SAUSA300_0715 | SAUSA300_RS03840 | *nrdI* | 9.43E-14 | 3.5 | protein NrdI |  |
| SAUSA300_0697 | SAUSA300_RS03740 |  | 4.72E-14 | 3.4 | 7-cyano-7-deazaguanine synthase |  |
| SAUSA300_1677 | SAUSA300_RS09160 |  | 3.50E-19 | 3.4 | iron-regulated surface determinant protein H | [P] |
| SAUSA300_0048 | SAUSA300_RS00255 |  | 1.87E-06 | 3.3 | hypothetical protein |  |
| SAUSA300_0958 | SAUSA300_RS05150 |  | 9.47E-28 | 3.3 | hypothetical protein |  |
| SAUSA300_1374 | SAUSA300_RS07505 |  | 8.06E-28 | 3.2 | hypothetical protein | [H] |
| SAUSA300_1326 | SAUSA300_RS07230 |  | 4.42E-12 | 3.2 | ribonuclease H | [R] |
| SAUSA300_0903 | SAUSA300_RS04860 |  | 1.07E-23 | 3.1 | hypothetical protein | [O] |
| SAUSA300_1344 | SAUSA300_RS07330 |  | 6.04E-18 | 3.1 | chromosome replication protein DnaD | [L] |
| SAUSA300_0694 | SAUSA300_RS03725 |  | 7.37E-15 | 3.1 | membrane protein |  |
| SAUSA300_2402 | SAUSA300_RS13300 |  | 4.61E-21 | 3.1 | prevent-host-death protein |  |
| SAUSA300_0401 | SAUSA300_RS02150 |  | 7.33E-11 | 3.1 | hypothetical protein | [R] |
| SAUSA300_1342 | SAUSA300_RS07320 |  | 4.02E-19 | 3.1 | hypothetical protein |  |
| SAUSA300_1183 | SAUSA300_RS06395 |  | 5.75E-30 | 3.1 | 2-oxoacid ferredoxin oxidoreductase subunit beta |  |
| SAUSA300_1513 | SAUSA300_RS08260 |  | 8.74E-14 | 3.1 | superoxide dismutase |  |
| SAUSA300_0545 | SAUSA300_RS02910 |  | 3.01E-27 | 3 | FMN-dependent NADPH-azoreductase | [C] |
| SAUSA300_0445 | SAUSA300_RS02380 | *gltB* | 2.62E-08 | 3 | glutamate synthase | [E] |
| SAUSA300_1580 | SAUSA300_RS08610 |  | 3.57E-12 | 3 | hypothetical protein |  |
| SAUSA300_2474 | SAUSA300_RS13730 |  | 8.54E-20 | 3 | thiol reductase thioredoxin | [O] |
| SAUSA300_0417 | SAUSA300_RS02235 |  | 2.11E-06 | 3 | hypothetical protein |  |
| SAUSA300_1376 | SAUSA300_RS07515 |  | 1.04E-07 | 3 | hypothetical protein |  |
| SAUSA300_1343 | SAUSA300_RS07325 | *nth* | 6.66E-20 | 3 | endonuclease III | [L][K][R] |
| SAUSA300_2300 | SAUSA300_RS12705 |  | 1.07E-17 | 3 | TetR family transcriptional regulator | [K] |
| SAUSA300_2413 | SAUSA300_RS13360 |  | 4.72E-03 | 3 | hypothetical protein |  |
| SAUSA300_2401 | SAUSA300_RS13295 |  | 4.86E-22 | 2.9 | addiction module protein |  |
| SAUSA300_1725 | SAUSA300_RS09425 | ***tal*** | 2.50E-17 | 2.9 | transaldolase | [G] |
| SAUSA300_0957 | SAUSA300_RS05145 |  | 1.58E-21 | 2.9 | hypothetical protein |  |
| SAUSA300_0784 | SAUSA300_RS04235 |  | 1.18E-14 | 2.9 | hypothetical protein | [E][P] |
| SAUSA300_0732 | SAUSA300_RS03940 |  | 1.51E-19 | 2.9 | hypothetical protein |  |
| SAUSA300_0823 | SAUSA300_RS04440 |  | 6.56E-03 | 2.9 | membrane protein |  |
| SAUSA300_2257 | SAUSA300_RS12470 |  | 9.04E-21 | 2.9 | hypothetical protein |  |
| SAUSA300_2498 | SAUSA300_RS13875 | *crtN* | 3.54E-13 | 2.9 | dehydrosqualene desaturase | [E] |
| SAUSA300_0395 | SAUSA300_RS02110 |  | 1.33E-11 | 2.9 | hypothetical protein | [R] |
| SAUSA300_2614 | SAUSA300_RS14530 |  | 9.55E-13 | 2.9 | polysaccharide deacetylase | [G] |
| SAUSA300_2596 | SAUSA300_RS14435 | *cap1C* | 5.26E-07 | 2.8 | capsular polysaccharide biosynthesis protein Cap8C |  |
| SAUSA300_1208 | SAUSA300_RS06535 |  | 5.44E-09 | 2.8 | hypothetical protein |  |
| SAUSA300_1214 | SAUSA300_RS06585 | ***gLY1*** | 2.48E-03 | 2.8 | threonine aldolase | [J] |
| SAUSA300_0985 | SAUSA300_RS05300 |  | 3.45E-10 | 2.8 | NrdH-redoxin | [O] |
| SAUSA300_1877 | SAUSA300_RS10270 |  | 1.07E-23 | 2.8 | hypothetical protein |  |
| SAUSA300_1178 | SAUSA300_RS06370 | *recA* | 1.01E-11 | 2.8 | DNA recombination/repair protein RecA | [L][K] |
| SAUSA300_2414 | SAUSA300_RS13365 |  | 5.60E-03 | 2.8 | diaminopimelate epimerase |  |
| SAUSA300_2570 | SAUSA300_RS14295 | *arcA* | 9.76E-06 | 2.8 | arginine deiminase | [E] |
| SAUSA300_2509 | SAUSA300_RS13930 |  | 1.33E-13 | 2.8 | TetR family transcriptional regulator | [K] |
| SAUSA300_1294 | SAUSA300_RS07030 |  | 6.22E-08 | 2.8 | protein msa |  |
| SAUSA300_2544 | SAUSA300_RS14125 |  | 1.53E-09 | 2.8 | hypothetical protein |  |
| SAUSA300_0029 | SAUSA300_RS00150 |  | 8.74E-14 | 2.7 | nucleoid-structuring protein H-NS |  |
| SAUSA300_1256 | SAUSA300_RS06825 | *msrA* | 2.72E-15 | 2.7 | peptide-methionine (S)-S-oxide reductase | [O] |
| SAUSA300_0418 | SAUSA300_RS02240 |  | 5.94E-07 | 2.7 | hypothetical protein |  |
| SAUSA300_0552 | SAUSA300_RS02945 |  | 5.36E-24 | 2.7 | bacillithiol biosynthesis deacetylase BshB2 |  |
| SAUSA300_2160 | SAUSA300_RS11905 |  | 7.84E-09 | 2.7 | MerR family transcriptional regulator | [K] |
| SAUSA300_2603 | SAUSA300_RS14475 | *lip* | 2.28E-10 | 2.6 | lipase | [R][P] |
| SAUSA300_2279 | SAUSA300_RS12595 |  | 4.96E-07 | 2.6 | LysR family transcriptional regulator |  |
| SAUSA300_1276 | SAUSA300_RS06925 | *opp-2B* | 2.34E-03 | 2.6 | peptide ABC transporter permease | [P] |
| SAUSA300_0704 | SAUSA300_RS03785 |  | 6.24E-19 | 2.6 | heme ABC transporter ATP-binding protein | [K][R] |
| SAUSA300_1180 | SAUSA300_RS06380 |  | 7.05E-16 | 2.6 | hypothetical protein |  |
| SAUSA300_1985 | SAUSA300_RS10915 | *sdrH* | 5.55E-13 | 2.6 | hypothetical protein |  |
| SAUSA300_2590 | SAUSA300_RS14400 |  | 1.22E-12 | 2.6 | flavin reductase | [R] |
| SAUSA300_0438 | SAUSA300_RS02345 |  | 2.41E-06 | 2.5 | N-acetylmuramoyl-L-alanine amidase | [S] |
| SAUSA300_0304 | SAUSA300_RS01620 |  | 2.06E-13 | 2.5 | membrane protein |  |
| SAUSA300_2348 | SAUSA300_RS12960 |  | 1.08E-06 | 2.5 | N-acetyltransferase |  |
| SAUSA300_0696 | SAUSA300_RS03735 |  | 3.44E-10 | 2.5 | 6-pyruvoyl tetrahydrobiopterin synthase-like protein | [H] |
| SAUSA300_0705 | SAUSA300_RS03790 | *recQ* | 3.51E-16 | 2.5 | ATP-dependent DNA helicase RecQ | [L][O][R] |
| SAUSA300_0695 | SAUSA300_RS03730 |  | 2.29E-08 | 2.5 | 7-carboxy-7-deazaguanine synthase | [R] |
| SAUSA300_0303 | SAUSA300_RS01615 |  | 4.19E-07 | 2.5 | hypothetical protein |  |
| SAUSA300_1987 | SAUSA300_RS10925 |  | 4.09E-11 | 2.5 | hydrolase in agr operon |  |
| SAUSA300_0547 | SAUSA300_RS02920 | *sdrD* | 2.51E-11 | 2.5 | serine-aspartate repeat-containing protein D | [P] [M] |
| SAUSA300_0936 | SAUSA300_RS05030 |  | 1.40E-03 | 2.5 | ABC transporter ATP-binding protein | [R] |
| SAUSA300_0644 | SAUSA300_RS03455 |  | 2.46E-12 | 2.5 | 3-beta hydroxysteroid dehydrogenase | [R] |
| SAUSA300_0741 | SAUSA300_RS04000 | *uvrB* | 4.10E-15 | 2.5 | excinuclease ABC subunit B | [L][O][K] |
| SAUSA300_0393 | SAUSA300_RS02100 |  | 9.99E-03 | 2.5 | membrane protein |  |
| SAUSA300_0390 | SAUSA300_RS02080 |  | 2.08E-05 | 2.5 | hypothetical protein |  |
| SAUSA300_2571 | SAUSA300_RS14305 | *argR* | 5.32E-03 | 2.4 | ArgR family transcriptional regulator | [K] |
| SAUSA300_2386 | SAUSA300_RS13205 |  | 5.73E-13 | 2.4 | protein flp | [V] |
| SAUSA300_1447 | SAUSA300_RS07900 | *xerD* | 3.50E-19 | 2.4 | tyrosine recombinase XerD | [L][K] |
| SAUSA300_0410 | SAUSA300_RS02200 |  | 9.16E-03 | 2.4 | lipoprotein |  |
| SAUSA300_2444 | SAUSA300_RS13555 | *gntR* | 9.50E-11 | 2.4 | GntR family transcriptional regulator |  |
| SAUSA300_1049 | SAUSA300_RS05650 | *murI* | 5.77E-16 | 2.4 | glutamate racemase | [M][E] |
| SAUSA300_2467 | SAUSA300_RS13680 | *srtA* | 3.04E-15 | 2.4 | sortase A |  |
| SAUSA300_0839 | SAUSA300_RS04535 |  | 2.25E-06 | 2.4 | hypothetical protein |  |
| SAUSA300_0024 | SAUSA300_RS00125 |  | 6.65E-14 | 2.4 | metallo-hydrolase | [Q] |
| SAUSA300_1350 | SAUSA300_RS07360 |  | 4.04E-11 | 2.4 | hypothetical protein |  |
| SAUSA300_2419 | SAUSA300_RS13390 |  | 8.92E-08 | 2.4 | hypothetical protein |  |
| SAUSA300_0419 | SAUSA300_RS02245 |  | 4.74E-10 | 2.4 | hypothetical protein |  |
| SAUSA300_2591 | SAUSA300_RS14405 |  | 6.61E-05 | 2.4 | hypothetical protein |  |
| SAUSA300_0643 | SAUSA300_RS03450 |  | 4.51E-12 | 2.4 | acetyltransferase |  |
| SAUSA300_1903 | SAUSA300_RS10420 |  | 8.32E-05 | 2.4 | hypothetical protein |  |
| SAUSA300_0159 | SAUSA300_RS00840 | *cap5H* | 3.58E-02 | 2.3 | O-acetyltransferase |  |
| SAUSA300_0904 | SAUSA300_RS04865 |  | 8.96E-18 | 2.3 | hypothetical protein | [C] |
| SAUSA300_1232 | SAUSA300_RS06680 |  | 1.03E-11 | 2.3 | catalase | [P][C] |
| SAUSA300_1738 | SAUSA300_RS09505 |  | 1.82E-06 | 2.3 | hypothetical protein |  |
| SAUSA300_1452 | SAUSA300_RS07930 | *proC* | 5.70E-18 | 2.3 | pyrroline-5-carboxylate reductase | [E] |
| SAUSA300_2308 | SAUSA300_RS12755 |  | 1.04E-05 | 2.3 | DNA-binding response regulator | [K] |
| SAUSA300_1229 | SAUSA300_RS06660 |  | 3.70E-12 | 2.3 | haloacid dehalogenase |  |
| SAUSA300_0617 | SAUSA300_RS03310 |  | 1.09E-11 | 2.3 | sodium:proton antiporter | [R] |
| SAUSA300_1390 | SAUSA300_RS07585 |  | 2.93E-02 | 2.3 | hypothetical protein |  |
| SAUSA300_1194 | SAUSA300_RS06450 |  | 2.00E-07 | 2.3 | lysophospholipase | [R] |
| SAUSA300_1559 | SAUSA300_RS08505 |  | 2.81E-02 | 2.3 | enterotoxin | [R] |
| SAUSA300_2412 | SAUSA300_RS13355 |  | 2.91E-02 | 2.3 | hypothetical protein |  |
| SAUSA300_0716 | SAUSA300_RS03845 |  | 3.50E-06 | 2.3 | ribonucleotide-diphosphate reductase | [L] |
| SAUSA300_2499 | SAUSA300_RS13880 | *crtM* | 2.99E-07 | 2.3 | dehydrosqualene synthase | [J] |
| SAUSA300_0739 | SAUSA300_RS03985 |  | 1.99E-04 | 2.3 | peptidase M23 | [S] |
| SAUSA300_0551 | SAUSA300_RS02940 |  | 5.08E-17 | 2.3 | GTP cyclohydrolase FolE2 | [H] |
| SAUSA300_0298 | SAUSA300_RS01590 |  | 4.53E-04 | 2.3 | hypothetical protein |  |
| SAUSA300_0651 | SAUSA300_RS03490 |  | 4.53E-04 | 2.3 | peptidase M23 | [S] |
| SAUSA300_2613 | SAUSA300_RS14525 | *hisZ* | 4.89E-03 | 2.3 | ATP phosphoribosyltransferase regulatory subunit | [O][E] |
| SAUSA300_0446 | SAUSA300_RS02385 | *gltD* | 9.57E-07 | 2.3 | glutamate synthase subunit beta |  |
| SAUSA300_0378 | SAUSA300_RS02015 |  | 3.97E-08 | 2.3 | hypothetical protein |  |
| SAUSA300_0436 | SAUSA300_RS02335 |  | 3.84E-02 | 2.3 | ABC transporter permease | [P] |
| SAUSA300_0945 | SAUSA300_RS05080 |  | 4.95E-12 | 2.3 | isochorismate synthase |  |
| SAUSA300_1375 | SAUSA300_RS07510 |  | 1.41E-04 | 2.2 | hypothetical protein |  |
| SAUSA300_1702 | SAUSA300_RS09295 |  | 2.19E-06 | 2.2 | peptidase | [P] |
| SAUSA300_2276 | SAUSA300_RS12580 |  | 5.76E-04 | 2.2 | N-acetyl-diaminopimelate deacetylase |  |
| SAUSA300_2008 | SAUSA300_RS11045 | *ilvN* | 3.29E-02 | 2.2 | acetolactate synthase | [T] |
| SAUSA300_2542 | SAUSA300_RS14115 |  | 1.50E-14 | 2.2 | acetyl-CoA synthetase |  |
| SAUSA300_1641 | SAUSA300_RS08955 | *gltA* | 2.10E-14 | 2.2 | citrate synthase | [O][C] |
| SAUSA300_0553 | SAUSA300_RS02950 |  | 2.08E-14 | 2.2 | hypothetical protein |  |
| SAUSA300_0118 | SAUSA300_RS00610 |  | 6.88E-16 | 2.2 | siderophore biosynthesis protein SbnA |  |
| SAUSA300_2282 | SAUSA300_RS12610 |  | 3.40E-11 | 2.2 | lysostaphin resistance protein A |  |
| SAUSA300_2443 | SAUSA300_RS13550 | *gntK* | 2.31E-04 | 2.2 | gluconokinase | [G] |
| SAUSA300_0080 | SAUSA300_RS00410 |  | 1.52E-06 | 2.2 | hypothetical protein |  |
| SAUSA300_2316 | SAUSA300_RS12795 |  | 2.80E-06 | 2.2 | acetyltransferase |  |
| SAUSA300_2284 | SAUSA300_RS12620 |  | 9.59E-07 | 2.2 | molybdenum cofactor biosysynthesis protein | [S] |
| SAUSA300_0049 | SAUSA300_RS00260 |  | 3.72E-06 | 2.2 | hypothetical protein |  |
| SAUSA300_2480 | SAUSA300_RS13760 | ***cidR*** | 1.62E-08 | 2.2 | LysR family transcriptional regulator |  |
| SAUSA300_1088 | SAUSA300_RS05895 |  | 7.07E-10 | 2.2 | glyoxalase |  |
| SAUSA300_2164 | SAUSA300_RS11930 |  | 5.21E-06 | 2.2 | toxin | [R] |
| SAUSA300_0612 | SAUSA300_RS03285 |  | 1.72E-02 | 2.2 | cation:proton antiporter |  |
| SAUSA300_0127 | SAUSA300_RS00660 |  | 2.54E-03 | 2.2 | hypothetical protein |  |
| SAUSA300_1723 | SAUSA300_RS09415 |  | 6.73E-09 | 2.1 | membrane protein |  |
| SAUSA300_1612 | SAUSA300_RS08785 | *tag* | 6.68E-10 | 2.1 | DNA-3-methyladenine glycosylase | [L] |
| SAUSA300_1454 | SAUSA300_RS07940 | *zwf* | 2.60E-12 | 2.1 | glucose-6-phosphate dehydrogenase | [R][G] |
| SAUSA300_2465 | SAUSA300_RS13670 |  | 6.96E-03 | 2.1 | peptide ABC transporter ATP-binding protein | [R] |
| SAUSA300_1081 | SAUSA300_RS05860 |  | 2.56E-08 | 2.1 | Laccase SACOL1200 |  |
| SAUSA300_0152 | SAUSA300_RS00805 | *cap5A* | 3.40E-02 | 2.1 | capsular polysaccharide type 5 biosynthesis protein cap5A | [P] [M] |
| SAUSA300_2005 | SAUSA300_RS11025 |  | 4.09E-07 | 2.1 | tRNA threonylcarbamoyladenosine biosynthesis protein TsaE | [J] |
| SAUSA300_0842 | SAUSA300_RS04550 |  | 3.98E-12 | 2.1 | hypothetical protein |  |
| SAUSA300_0262 | SAUSA300_RS01405 | *rbsK* | 1.16E-08 | 2.1 | ribokinase | [G] |
| SAUSA300_1745 | SAUSA300_RS09535 |  | 2.82E-05 | 2.1 | hypothetical protein |  |
| SAUSA300_1327 | SAUSA300_RS07235 |  | 4.51E-06 | 2.1 | hyperosmolarity resistance protein Ebh | [R][P] |
| SAUSA300_1287 | SAUSA300_RS06995 | *asd* | 1.45E-04 | 2.1 | aspartate-semialdehyde dehydrogenase | [E][R][O] |
| SAUSA300_1609 | SAUSA300_RS08765 |  | 2.04E-02 | 2.1 | peptidase A24 |  |
| SAUSA300_0787 | SAUSA300_RS04250 | *aroD* | 3.21E-04 | 2.1 | 3-dehydroquinase |  |
| SAUSA300_1668 | SAUSA300_RS09105 |  | 2.65E-08 | 2.1 | peroxiredoxin |  |
| SAUSA300_2128 | SAUSA300_RS11720 |  | 4.25E-09 | 2.1 | multidrug MFS transporter |  |
| SAUSA300_0642 | SAUSA300_RS03445 |  | 3.28E-10 | 2.1 | hypothetical protein |  |
| SAUSA300_0740 | SAUSA300_RS03990 |  | 1.10E-06 | 2.1 | hydrolase |  |
| SAUSA300_1721 | SAUSA300_RS09400 |  | 1.46E-08 | 2.1 | hypothetical protein |  |
| SAUSA300_1842 | SAUSA300_RS10060 |  | 2.80E-05 | 2.1 | transcriptional repressor |  |
| SAUSA300_0918 | SAUSA300_RS04935 |  | 8.74E-11 | 2.1 | diacylglycerol beta-glucosyltransferase | [M] |
| SAUSA300_1736 | SAUSA300_RS09490 |  | 3.46E-11 | 2.1 | membrane protein insertion efficiency factor YidD |  |
| SAUSA300_0047 | SAUSA300_RS00250 |  | 6.72E-05 | 2.1 | hypothetical protein | [S] |
| SAUSA300_1693 | SAUSA300_RS09250 |  | 2.04E-10 | 2 | hypothetical protein | [Q] |
| SAUSA300_2258 | SAUSA300_RS12475 |  | 3.22E-15 | 2 | formate dehydrogenase subunit alpha |  |
| SAUSA300_2094 | SAUSA300_RS11535 |  | 1.58E-08 | 2 | EVE domain-containing protein | [K][R] |
| SAUSA300_1629 | SAUSA300_RS08885 | *thrS* | 2.69E-04 | 2 | threonine--tRNA ligase | [R][O][J][U] |
| SAUSA300_0623 | SAUSA300_RS03340 | *tagA* | 1.29E-09 | 2 | N-acetylmannosaminyltransferase |  |
| SAUSA300_1372 | SAUSA300_RS07495 |  | 1.76E-07 | 2 | hypothetical protein |  |
| SAUSA300_0382 | SAUSA300_RS02035 | ***tcyP*** | 1.81E-06 | 2 | L-cystine transporter tcyP | [E] |
| SAUSA300_2211 | SAUSA300_RS12195 |  | 1.80E-05 | 2 | malonate transporter |  |
| SAUSA300_2546 | SAUSA300_RS14135 | *betB* | 4.55E-05 | 2 | betaine-aldehyde dehydrogenase |  |
| SAUSA300_0742 | SAUSA300_RS04005 | *uvrA* | 1.71E-10 | 2 | ABC-ATPase UvrA | [R][K] |
| SAUSA300_0645 | SAUSA300_RS03460 |  | 3.69E-08 | 2 | DNA-binding response regulator | [K] |
| SAUSA300_0299 | SAUSA300_RS01595 |  | 6.25E-03 | 2 | hypothetical protein |  |
| SAUSA300_2109 | SAUSA300_2109 | *fmtB* | 1.67E-04 | 2 |  |  |
| SAUSA300_1050 | SAUSA300_RS05655 |  | 9.03E-10 | 2 | non-canonical purine NTP pyrophosphatase |  |
| *Downregulated genes* |  |  |  |  |  |  |
| SAUSA300_1794 | SAUSA300_RS09820 |  | 9.09E-08 | -226.6 | hypothetical protein |  |
| SAUSA300_1920 | SAUSA300_RS10530 | *chs* | 1.90E-141 | -43.7 | chemotaxis inhibitory protein |  |
| SAUSA300_0311 | SAUSA300_RS01660 |  | 1.38E-37 | -27.4 | carbohydrate kinase |  |
| SAUSA300_2364 | SAUSA300_RS13060 | *sbi* | 2.43E-60 | -21.9 | immunoglobulin-binding protein sbi | [R][B] |
| SAUSA300_0312 | SAUSA300_RS01665 |  | 1.98E-40 | -21 | pseudouridine-5'-phosphate glycosidase | [F] |
| SAUSA300_1416 | SAUSA300_RS07725 |  | 0.003185 | -16.6 | hypothetical protein |  |
| SAUSA300_0371 | SAUSA300_RS01975 |  | 7.83E-05 | -15.2 | hypothetical protein |  |
| SAUSA300_0986 | SAUSA300_RS05305 | ***cydA*** | 2.20E-82 | -14.7 | cytochrome d ubiquinol oxidase subunit I | [P] |
| SAUSA300_0987 | SAUSA300_RS05310 | ***cydB*** | 9.37E-69 | -13.7 | cytochrome D ubiquinol oxidase subunit II | [P] |
| SAUSA300_0215 | SAUSA300_RS01130 |  | 1.55E-23 | -12.8 | membrane protein | [S] |
| SAUSA300_0998 | SAUSA300_RS05375 |  | 3.54E-44 | -12 | Cro/Cl family transcriptional regulator | [R][K] |
| SAUSA300_1056 | SAUSA300_RS05695 |  | 2.44E-62 | -11.4 | fibrinogen-binding protein |  |
| SAUSA300_1000 | SAUSA300_RS05385 | *potB* | 8.42E-28 | -11.1 | spermidine/putrescine ABC transporter permease | [P] |
| SAUSA300_1055 | SAUSA300_RS05690 | *efb* | 1.84E-50 | -11 | fibrinogen-binding protein |  |
| SAUSA300_2383 | SAUSA300_RS13180 |  | 1.70E-123 | -10.8 | alanine glycine permease | [E][P] |
| SAUSA300_0721 | SAUSA300_RS03880 |  | 9.30E-101 | -10.3 | hypothetical protein | [M] |
| SAUSA300_1001 | SAUSA300_RS05390 | *potC* | 4.72E-43 | -9.8 | spermidine/putrescine ABC transporter permease | [P] |
| SAUSA300_0999 | SAUSA300_RS05380 | *potA* | 1.92E-46 | -9.3 | spermidine/putrescine import ATP-binding protein PotA | [R][P] |
| SAUSA300_0773 | SAUSA300_RS04170 |  | 3.48E-16 | -7.7 | staphylocoagulase |  |
| SAUSA300_0313 | SAUSA300_RS01670 |  | 2.70E-18 | -7.5 | pyrimidine nucleoside transporter NupC | [P] |
| SAUSA300_1052 | SAUSA300_RS05670 |  | 2.59E-51 | -7.5 | fibrinogen-binding protein |  |
| SAUSA300_1981 | SAUSA300_RS10885 |  | 8.31E-05 | -6.7 | phage terminase small subunit | [X] |
| SAUSA300_1334 | SAUSA300_RS07270 |  | 3.74E-17 | -6.7 | sulfite reductase subunit alpha |  |
| SAUSA300_0355 | SAUSA300_RS01885 |  | 5.81E-50 | -6.7 | acetyl-CoA acyltransferase |  |
| SAUSA300_2349 | SAUSA300_RS12965 |  | 1.04E-41 | -6.7 | formate/nitrite transporter | [P] |
| SAUSA300_0693 | SAUSA300_RS03720 |  | 1.13E-31 | -6.5 | hypothetical protein |  |
| SAUSA300_1331 | SAUSA300_RS07255 | *ald* | 1.03E-20 | -6.4 | alanine dehydrogenase | [E] |
| SAUSA300_1384 | SAUSA300_RS07555 |  | 0.034982 | -6.3 | holin | [M] |
| SAUSA300_0370 | SAUSA300_RS01970 |  | 6.28E-21 | -6.1 | toxin | [R] |
| SAUSA300_0720 | SAUSA300_RS03875 |  | 3.68E-31 | -6 | iron ABC transporter ATP-binding protein | [R] |
| SAUSA300_0940 | SAUSA300_RS05050 |  | 1.74E-24 | -6 | hypothetical protein |  |
| SAUSA300_2365 | SAUSA300_RS13070 | *hlgA* | 7.11E-25 | -5.8 | gamma-hemolysin component A | [R] |
| SAUSA300_0224 | SAUSA300_RS01180 | *coa* | 2.20E-29 | -5.5 | coagulase |  |
| SAUSA300_0538 | SAUSA300_RS02875 |  | 1.91E-48 | -5.5 | UDP-glucose 4-epimerase | [R] |
| SAUSA300_1330 | SAUSA300_RS07250 | *ilvA* | 3.65E-21 | -5.5 | serine/threonine dehydratase |  |
| SAUSA300_0719 | SAUSA300_RS03870 |  | 1.36E-29 | -5.5 | iron ABC transporter permease | [P] |
| SAUSA300_0307 | SAUSA300_RS01635 |  | 5.89E-49 | -5.3 | 5'-nucleotidase |  |
| SAUSA300_0407 | SAUSA300_RS02185 |  | 5.65E-06 | -5.2 | hypothetical protein | [R] |
| SAUSA300_0692 | SAUSA300_RS03715 |  | 8.48E-48 | -5.1 | hypothetical protein |  |
| SAUSA300_0257 | SAUSA300_RS01370 |  | 3.70E-30 | -5 | antiholin-like protein LrgB |  |
| SAUSA300_1399 | SAUSA300_RS07635 |  | 0.041717 | -4.9 | hypothetical protein |  |
| SAUSA300_0683 | SAUSA300_RS03665 |  | 6.77E-30 | -4.9 | DeoR family transcriptional regulator | [R][Q] |
| SAUSA300_1002 | SAUSA300_RS05395 | *potD* | 1.29E-43 | -4.8 | spermidine/putrescine ABC transporter substrate-binding protein |  |
| SAUSA300_1545 | SAUSA300_RS08425 | *rpsT* | 2.66E-17 | -4.6 | 30S ribosomal protein S20 | [T] |
| SAUSA300_0775 | SAUSA300_RS04180 |  | 1.12E-05 | -4.6 | hypothetical protein |  |
| SAUSA300_0253 | SAUSA300_RS01350 | *scdA* | 1.10E-44 | -4.4 | iron-sulfur cluster repair protein ScdA |  |
| SAUSA300_0074 | SAUSA300_RS00375 | *opp-3B* | 9.14E-28 | -4.4 | peptide ABC transporter permease | [P] |
| SAUSA300_2526 | SAUSA300_RS14015 | *pyrD* | 1.34E-36 | -4.4 | dihydroorotate dehydrogenase 2 | [P] [O][F] |
| SAUSA300_2366 | SAUSA300_RS13075 | *hlgC* | 8.07E-19 | -4.3 | gamma-hemolysin component C | [R] |
| SAUSA300_0815 | SAUSA300_RS04395 | *ear* | 1.91E-39 | -4.3 | hypothetical protein |  |
| SAUSA300_0691 | SAUSA300_RS03710 | *saeR* | 1.55E-23 | -4.3 | DNA-binding response regulator | [K] |
| SAUSA300_2081 | SAUSA300_RS11455 | *pyrG* | 1.31E-29 | -4.2 | CTP synthetase | [F] |
| SAUSA300_1017 | SAUSA300_RS05470 |  | 1.82E-42 | -4.2 | membrane protein | [P] |
| SAUSA300_0073 | SAUSA300_RS00370 |  | 1.71E-22 | -4.2 | nickel ABC transporter substrate-binding protein |  |
| SAUSA300_0256 | SAUSA300_RS01365 |  | 2.75E-17 | -4.2 | antiholin-like protein LrgA |  |
| SAUSA300_0075 | SAUSA300_RS00380 | *opp-3C* | 1.32E-13 | -4.1 | membrane protein | [P] |
| SAUSA300_1419 | SAUSA300_RS07740 |  | 0.025088 | -4 | hypothetical protein |  |
| SAUSA300_2351 | SAUSA300_RS12980 |  | 1.10E-05 | -4 | zinc ABC transporter substrate-binding protein | [P] |
| SAUSA300_2091 | SAUSA300_RS11520 | *deoD* | 7.05E-28 | -4 | purine-nucleoside phosphorylase | [F] |
| SAUSA300_2063 | SAUSA300_RS11360 | *atpE* | 2.31E-17 | -3.9 | ATP synthase subunit C |  |
| SAUSA300_2486 | SAUSA300_RS13805 |  | 1.34E-21 | -3.9 | ATP-dependent Clp protease ATP-binding subunit |  |
| SAUSA300_0885 | SAUSA300_RS04770 | *fabH* | 3.87E-38 | -3.9 | 3-oxoacyl-ACP synthase III |  |
| SAUSA300_0372 | SAUSA300_RS01980 |  | 3.44E-18 | -3.9 | peptidase |  |
| SAUSA300_0594 | SAUSA300_RS03190 | *adh* | 2.15E-07 | -3.9 | zinc-dependent alcohol dehydrogenase |  |
| SAUSA300_2124 | SAUSA300_RS11695 | *rrsE* | 0.000548 | -3.9 | 16S ribosomal RNA |  |
| SAUSA300_0172 | SAUSA300_RS00905 |  | 1.76E-08 | -3.8 | hypothetical protein |  |
| SAUSA300_1037 | SAUSA300_RS05585 | *pheS* | 1.64E-28 | -3.8 | phenylalanine--tRNA ligase subunit alpha | [R][J][O] |
| SAUSA300_2537 | SAUSA300_RS14075 |  | 3.66E-12 | -3.8 | L-lactate dehydrogenase | [O] |
| SAUSA300_2621 | SAUSA300_RS14565 |  | 6.95E-21 | -3.8 | lactonase drp35 |  |
| SAUSA300_2291 | SAUSA300_RS12660 | *gltS* | 2.17E-25 | -3.8 | sodium:glutamate symporter | [H] |
| SAUSA300_1742 | SAUSA300_RS09520 |  | 3.81E-19 | -3.8 | hypothetical protein |  |
| SAUSA300_0076 | SAUSA300_RS00385 |  | 2.20E-14 | -3.8 | ABC transporter ATP-binding protein | [R] |
| SAUSA300_1711 | SAUSA300_RS09350 | *putA* | 4.27E-31 | -3.7 | proline dehydrogenase | [E] |
| SAUSA300_0668 | SAUSA300_RS03585 |  | 4.77E-30 | -3.7 | hypothetical protein |  |
| SAUSA300_0235 | SAUSA300_RS01250 | ***lctE*** | 8.66E-08 | -3.7 | L-lactate dehydrogenase | [O] |
| SAUSA300_2084 | SAUSA300_RS11475 | *coaA* | 1.80E-25 | -3.7 | type II pantothenate kinase |  |
| SAUSA300_0690 | SAUSA300_RS03705 | *saeS* | 6.86E-30 | -3.7 | two-component sensor histidine kinase | [T] |
| SAUSA300_0077 | SAUSA300_RS00390 |  | 4.79E-11 | -3.7 | NAD+ synthetase | [R] |
| SAUSA300_2328 | SAUSA300_RS12865 |  | 2.23E-39 | -3.7 | hypothetical protein |  |
| SAUSA300_1883 | SAUSA300_RS10305 | *putP* | 1.54E-34 | -3.7 | sodium:proline symporter | [R][P] |
| SAUSA300_2626 | SAUSA300_RS14590 |  | 6.79E-15 | -3.7 | DNA damage-inducible protein DinB |  |
| SAUSA300_0072 | SAUSA300_RS00365 |  | 6.83E-15 | -3.6 | methyltransferase | [E] |
| SAUSA300_1917 | SAUSA300_1917 | *map* | 4.55E-19 | -3.6 |  |  |
| SAUSA300_1038 | SAUSA300_RS05590 | *pheT* | 1.69E-37 | -3.6 | phenylalanine--tRNA ligase subunit beta | [O][T] |
| SAUSA300_0108 | SAUSA300_RS00560 |  | 2.49E-16 | -3.5 | oleate hydratase | [R] |
| SAUSA300_1841 | SAUSA300_RS10055 | *rrsC* | 0.000707 | -3.5 | 16S ribosomal RNA |  |
| SAUSA300_1053 | SAUSA300_RS05680 |  | 1.44E-19 | -3.5 | FPRL1 inhibitory protein |  |
| SAUSA300_2059 | SAUSA300_RS11340 | *atpG* | 1.05E-14 | -3.5 | ATP synthase subunit gamma | [C] |
| SAUSA300_2061 | SAUSA300_RS11350 | *atpH* | 1.56E-27 | -3.5 | ATP synthase subunit delta | [P] |
| SAUSA300_1016 | SAUSA300_RS05465 | *cyoE* | 2.94E-29 | -3.4 | protoheme IX farnesyltransferase |  |
| SAUSA300_2441 | SAUSA300_RS13530 | *fnbA* | 2.90E-18 | -3.4 | fibronectin-binding protein A | [P] [M] |
| SAUSA300_2484 | SAUSA300_RS13795 |  | 6.41E-18 | -3.4 | hydroxymethylglutaryl-CoA synthase |  |
| SAUSA300_1743 | SAUSA300_RS09525 |  | 9.40E-05 | -3.3 | hypothetical protein |  |
| SAUSA300_2057 | SAUSA300_RS11330 | *atpC* | 8.60E-09 | -3.3 | ATP synthase epsilon chain |  |
| SAUSA300_0574 | SAUSA300_RS03070 |  | 4.23E-19 | -3.3 | phosphomevalonate kinase | [O] |
| SAUSA300_2367 | SAUSA300_RS13080 | *hlgB* | 2.37E-16 | -3.3 | gamma-hemolysin component B | [R] |
| SAUSA300_0221 | SAUSA300_RS01160 | *pflA* | 1.83E-06 | -3.3 | pyruvate formate-lyase-activating enzyme | [S][R] |
| SAUSA300_0590 | SAUSA300_RS03170 |  | 8.43E-17 | -3.3 | NAD(P)H-dependent oxidoreductase | [C] |
| SAUSA300_0337 | SAUSA300_RS01790 | *glpT* | 4.37E-17 | -3.3 | glycerol-3-phosphate transporter | [G][P] |
| SAUSA300_2060 | SAUSA300_RS11345 | *atpA* | 2.13E-11 | -3.2 | ATP synthase subunit alpha |  |
| SAUSA300_2058 | SAUSA300_RS11335 | *atpD* | 2.94E-10 | -3.2 | ATP synthase subunit beta |  |
| SAUSA300_0718 | SAUSA300_RS03865 |  | 7.21E-18 | -3.2 | iron ABC transporter permease | [P] |
| SAUSA300_1741 | SAUSA300_1741 |  | 0.000482 | -3.2 |  |  |
| SAUSA300_2064 | SAUSA300_RS11365 | *atpB* | 1.03E-12 | -3.2 | ATP synthase subunit A |  |
| SAUSA300_2640 | SAUSA300_RS14660 |  | 9.85E-06 | -3.2 | transcriptional regulator | [K] |
| SAUSA300_1015 | SAUSA300_RS05460 | *ctaA* | 2.29E-25 | -3.2 | heme A synthase | [P] |
| SAUSA300_2491 | SAUSA300_RS13840 |  | 3.52E-09 | -3.1 | 1-pyrroline-5-carboxylate dehydrogenase |  |
| SAUSA300_0234 | SAUSA300_RS01240 | ***hmp*** | 2.29E-25 | -3.1 | nitric oxide dioxygenase | [C][P] |
| SAUSA300_1948 | SAUSA300_RS10685 |  | 5.97E-07 | -3.1 | hypothetical protein |  |
| SAUSA300_0315 | SAUSA300_RS01680 | *nanA* | 1.54E-10 | -3.1 | N-acetylneuraminate lyase | [O][J] |
| SAUSA300_1026 | SAUSA300_RS05520 |  | 1.05E-26 | -3 | DNA-binding protein |  |
| SAUSA300_2062 | SAUSA300_RS11355 | *atpF* | 1.15E-08 | -3 | ATP synthase subunit B |  |
| SAUSA300_0573 | SAUSA300_RS03065 | *mvaD* | 2.43E-16 | -3 | diphosphomevalonate decarboxylase |  |
| SAUSA300_0149 | SAUSA300_0149 |  | 0.009853 | -3 |  |  |
| SAUSA300_0223 | SAUSA300_RS01175 |  | 3.78E-15 | -3 | hypothetical protein |  |
| SAUSA300_0130 | SAUSA300_RS00680 |  | 0.014294 | -2.9 | NAD-dependent dehydratase | [R] |
| SAUSA300_2313 | SAUSA300_RS12780 |  | 4.19E-13 | -2.9 | L-lactate permease | [C] |
| SAUSA300_1009 | SAUSA300_RS05430 | *typA* | 1.96E-22 | -2.9 | GTP-binding protein | [D][T] |
| SAUSA300_1829 | SAUSA300_RS09995 |  | 7.49E-05 | -2.9 | tRNA-Pro |  |
| SAUSA300_0233 | SAUSA300_RS01235 |  | 5.25E-12 | -2.9 | membrane protein |  |
| SAUSA300_1153 | SAUSA300_RS06245 | *uppS* | 3.12E-21 | -2.9 | isoprenyl transferase |  |
| SAUSA300_0785 | SAUSA300_RS04240 |  | 8.63E-16 | -2.8 | acetyltransferase |  |
| SAUSA300_1586 | SAUSA300_RS08645 | *aspS* | 3.00E-25 | -2.8 | aspartate--tRNA ligase | [O][R][J] |
| SAUSA300_2380 | SAUSA300_RS13165 |  | 2.94E-08 | -2.8 | transcriptional regulator |  |
| SAUSA300_0194 | SAUSA300_RS01020 | ***murP*** | 7.21E-11 | -2.8 | permease | [P] |
| SAUSA300_0684 | SAUSA300_RS03670 | *fruB* | 4.52E-12 | -2.8 | 1-phosphofructokinase | [G] |
| SAUSA300_1675 | SAUSA300_RS09145 | *tyrS* | 5.04E-14 | -2.8 | tyrosine--tRNA ligase | [R][J][T][O] |
| SAUSA300_0220 | SAUSA300_RS01155 | *pflB* | 3.53E-06 | -2.8 | formate acetyltransferase | [O][S] |
| SAUSA300_0314 | SAUSA300_RS01675 |  | 8.40E-09 | -2.8 | hypothetical protein | [R][P] |
| SAUSA300_2270 | SAUSA300_RS12550 | *glvC* | 2.08E-11 | -2.7 | PTS alpha-glucoside transporter subunit IIBC | [P] |
| SAUSA300_1411 | SAUSA300_RS07700 |  | 0.011147 | -2.7 | hypothetical protein |  |
| SAUSA300_0638 | SAUSA300_RS03420 |  | 1.63E-08 | -2.7 | PTS mannose transporter subunit IIA |  |
| SAUSA300_1811 | SAUSA300_RS09910 |  | 2.12E-06 | -2.7 | tRNA-Leu |  |
| SAUSA300_0886 | SAUSA300_RS04775 | *fabF* | 1.36E-22 | -2.7 | beta-ketoacyl-[acyl-carrier-protein] synthase II |  |
| SAUSA300_0506 | SAUSA300_RS02710 | *nupC* | 4.81E-17 | -2.7 | pyrimidine nucleoside transporter NupC | [P][S] |
| SAUSA300_0364 | SAUSA300_RS01930 | *ychF* | 5.91E-18 | -2.7 | GTP-binding protein YchF | [D] |
| SAUSA300_0310 | SAUSA300_RS01655 | *pfoR* | 3.18E-16 | -2.7 | transcriptional regulator | [P] |
| SAUSA300_1566 | SAUSA300_RS08540 |  | 1.31E-08 | -2.7 | allophanate hydrolase | [E] |
| SAUSA300_2493 | SAUSA300_RS13850 |  | 2.12E-06 | -2.6 | hypothetical protein |  |
| SAUSA300_1235 | SAUSA300_RS06695 | *guaC* | 3.49E-14 | -2.6 | guanosine monophosphate reductase | [F] |
| SAUSA300_1121 | SAUSA300_RS06065 |  | 4.82E-24 | -2.6 | transcription factor FapR | [I] |
| SAUSA300_2123 | SAUSA300_RS11690 | *rrlE* | 0.00171 | -2.6 | 23S ribosomal RNA |  |
| SAUSA300_0192 | SAUSA300_RS01010 |  | 1.10E-06 | -2.6 | hypothetical protein |  |
| SAUSA300_1093 | SAUSA300_RS05925 | *pyrB* | 0.013044 | -2.6 | aspartate carbamoyltransferase | [P] |
| SAUSA300_2502 | SAUSA300_RS13895 |  | 1.84E-09 | -2.6 | glycosyl-4,4'-diaponeurosporenoate acyltransferase |  |
| SAUSA300_1666 | SAUSA300_RS09090 | *rpsD* | 1.52E-07 | -2.6 | 30S ribosomal protein S4 | [J][T][R] |
| SAUSA300_0357 | SAUSA300_RS01895 | *metE* | 1.46E-07 | -2.6 | 5-methyltetrahydropteroyltriglutamate--homocysteine methyltransferase |  |
| SAUSA300_0922 | SAUSA300_RS04955 |  | 6.63E-11 | -2.6 | membrane protein |  |
| SAUSA300_1192 | SAUSA300_RS06440 | *glpK* | 1.48E-10 | -2.6 | glycerol kinase | [C] |
| SAUSA300_1285 | SAUSA300_RS06985 |  | 4.23E-13 | -2.5 | ABC transporter ATP-binding protein | [R] |
| SAUSA300_0524 | SAUSA300_RS02805 | *rplJ* | 4.47E-05 | -2.5 | 50S ribosomal protein L10 | [R][J] |
| SAUSA300_1562 | SAUSA300_RS08520 |  | 2.05E-08 | -2.5 | hypothetical protein |  |
| SAUSA300_1124 | SAUSA300_RS06080 | *fabG* | 3.53E-13 | -2.5 | beta-ketoacyl-ACP reductase | [R] |
| SAUSA300_0019 | SAUSA300_RS00100 |  | 0.002774 | -2.5 | tRNA-Asp |  |
| SAUSA300_0498 | SAUSA300_RS02670 |  | 2.50E-06 | -2.5 | tRNA-Ala |  |
| SAUSA300_0363 | SAUSA300_RS01925 |  | 1.74E-12 | -2.5 | hypothetical protein |  |
| SAUSA300_0331 | SAUSA300_RS01760 |  | 0.000594 | -2.5 | PTS lactose transporter subunit IIB |  |
| SAUSA300_1091 | SAUSA300_RS05915 | *pyrR* | 7.81E-10 | -2.5 | bifunctional protein PyrR |  |
| SAUSA300_0358 | SAUSA300_RS01900 | ***metH*** | 2.44E-05 | -2.5 | bifunctional homocysteine S-methyltransferase/5,10-methylenetetrahydrofolate reductase protein | [E] |
| SAUSA300_0366 | SAUSA300_RS01940 | *rpsF* | 6.06E-06 | -2.5 | 30S ribosomal protein S6 | [J] |
| SAUSA300_2235 | SAUSA300_RS12340 |  | 2.01E-16 | -2.5 | ferrichrome ABC transporter substrate-binding protein | [M] |
| SAUSA300_2202 | SAUSA300_RS12140 | *rplW* | 3.01E-06 | -2.5 | 50S ribosomal protein L23 | [R][J] |
| SAUSA300_1963 | SAUSA300_RS10765 |  | 1.15E-09 | -2.5 | hypothetical protein |  |
| SAUSA300_2331 | SAUSA300_RS12880 |  | 2.81E-20 | -2.5 | transcriptional regulator | [K] |
| SAUSA300_0210 | SAUSA300_RS01105 |  | 2.77E-06 | -2.5 | maltose ABC transporter permease | [P] |
| SAUSA300_1508 | SAUSA300_RS08235 |  | 8.86E-10 | -2.5 | hypothetical protein |  |
| SAUSA300_0572 | SAUSA300_RS03060 | *mvk* | 2.05E-13 | -2.5 | mevalonate kinase | [O][I] |
| SAUSA300_0770 | SAUSA300_RS04155 |  | 0.024241 | -2.5 | membrane protein |  |
| SAUSA300_0208 | SAUSA300_RS01095 |  | 1.95E-05 | -2.5 | sugar ABC transporter ATP-binding protein | [R] |
| SAUSA300_0326 | SAUSA300_RS01735 |  | 1.38E-14 | -2.5 | hypothetical protein | [J] |
| SAUSA300_2195 | SAUSA300_RS12105 | *rpsQ* | 2.71E-08 | -2.5 | 30S ribosomal protein S17 | [R][J] |
| SAUSA300_0837 | SAUSA300_RS04525 | *dltC* | 1.36E-13 | -2.4 | D-alanine--poly(phosphoribitol) ligase subunit 2 |  |
| SAUSA300_0193 | SAUSA300_RS01015 | *murQ* | 1.04E-07 | -2.4 | N-acetylmuramic acid 6-phosphate etherase | [R][K][M] |
| SAUSA300_0884 | SAUSA300_RS04765 |  | 3.35E-11 | -2.4 | hypothetical protein |  |
| SAUSA300_0376 | SAUSA300_RS02005 |  | 2.73E-07 | -2.4 | membrane protein |  |
| SAUSA300_0195 | SAUSA300_RS01025 |  | 6.87E-12 | -2.4 | RpiR family transcriptional regulator | [K][R][M] |
| SAUSA300_2118 | SAUSA300_RS11665 |  | 1.76E-06 | -2.4 | tRNA-Tyr |  |
| SAUSA300_0333 | SAUSA300_RS01770 |  | 3.33E-06 | -2.4 | hypothetical protein | [M][K][P] |
| SAUSA300_0526 | SAUSA300_RS02815 |  | 1.46E-14 | -2.4 | methyltransferase | [J] |
| SAUSA300_1564 | SAUSA300_RS08530 | *accB* | 3.20E-07 | -2.4 | acetyl-CoA carboxylase biotin carboxyl carrier protein subunit |  |
| SAUSA300_0209 | SAUSA300_RS01100 |  | 0.001982 | -2.4 | maltose ABC transporter substrate-binding protein | [G] |
| SAUSA300_2082 | SAUSA300_RS11460 | *rpoE* | 5.24E-11 | -2.4 | DNA-directed RNA polymerase subunit delta |  |
| SAUSA300_1329 | SAUSA300_RS07245 |  | 2.42E-06 | -2.4 | amino acid permease | [P][E] |
| SAUSA300_1122 | SAUSA300_RS06070 | *plsX* | 1.95E-20 | -2.4 | phosphate acyltransferase |  |
| SAUSA300_1838 | SAUSA300_RS10040 | *rrlC* | 0.0014 | -2.4 | 23S ribosomal RNA |  |
| SAUSA300_2171 | SAUSA300_RS11985 | *rpsI* | 0.000284 | -2.4 | 30S ribosomal protein S9 | [J] |
| SAUSA300_0367 | SAUSA300_RS01945 | *ssb* | 0.000118 | -2.4 | single-stranded DNA-binding protein | [L] |
| SAUSA300_1756 | SAUSA300_RS09610 | *splC* | 0.024899 | -2.4 | serine protease SplC | [O] |
| SAUSA300_1123 | SAUSA300_RS06075 | *fabD* | 4.34E-20 | -2.4 | malonyl CoA-ACP transacylase | [M][J][I][Q] |
| SAUSA300_1919 | SAUSA300_RS10525 |  | 4.41E-05 | -2.4 | hypothetical protein |  |
| SAUSA300_2016 | SAUSA300_RS11090 | *rrlD* | 0.007045 | -2.4 | 23S ribosomal RNA |  |
| SAUSA300_2191 | SAUSA300_RS12085 | *rpsN* | 9.90E-08 | -2.3 | 30S ribosomal protein S14 type Z | [R][J] |
| SAUSA300_0318 | SAUSA300_RS01695 | ***nanE*** | 6.22E-08 | -2.3 | N-acetylmannosamine-6-phosphate 2-epimerase | [G] |
| SAUSA300_1028 | SAUSA300_RS05535 |  | 0.000157 | -2.3 | iron-regulated surface determinant protein B | [M][P] |
| SAUSA300_1027 | SAUSA300_RS05530 | *rpmF* | 1.70E-06 | -2.3 | 50S ribosomal protein L32 |  |
| SAUSA300_2017 | SAUSA300_RS11095 | *rrsD* | 0.010249 | -2.3 | 16S ribosomal RNA |  |
| SAUSA300_2641 | SAUSA300_RS14665 |  | 0.000592 | -2.3 | hypothetical protein |  |
| SAUSA300_2440 | SAUSA300_RS13525 | *fnbB* | 9.05E-11 | -2.3 | fibronectin-binding protein A | [P] [M] |
| SAUSA300_2495 | SAUSA300_RS13860 |  | 1.18E-09 | -2.3 | copper chaperone CopZ | [P] |
| SAUSA300_0327 | SAUSA300_RS01740 |  | 4.68E-12 | -2.3 | deacetylase SIR2 |  |
| SAUSA300_0961 | SAUSA300_RS05165 | *qoxC* | 6.74E-05 | -2.3 | quinol oxidase subunit 3 | [P] |
| SAUSA300_2543 | SAUSA300_RS14120 |  | 1.68E-08 | -2.3 | antibiotic biosynthesis monooxygenase | [R] |
| SAUSA300_0094 | SAUSA300_RS00490 |  | 0.00449 | -2.3 | hypothetical protein |  |
| SAUSA300_1563 | SAUSA300_RS08525 | *accC* | 1.97E-10 | -2.3 | acetyl-CoA carboxylase biotin carboxylase subunit |  |
| SAUSA300_2192 | SAUSA300_RS12090 | *rplE* | 8.75E-08 | -2.3 | 50S ribosomal protein L5 | [R][J] |
| SAUSA300_2194 | SAUSA300_RS12100 | *rplN* | 1.23E-05 | -2.3 | 50S ribosomal protein L14 | [J] |
| SAUSA300_2196 | SAUSA300_RS12110 | *rpmC* | 0.000241 | -2.3 | 50S ribosomal protein L29 | [R][J] |
| SAUSA300_0025 | SAUSA300_RS00130 |  | 3.92E-11 | -2.3 | 5'-nucleotidase family protein | [R] |
| SAUSA300_1758 | SAUSA300_RS09620 | *splA* | 0.002966 | -2.3 | serine protease SplA | [O] |
| SAUSA300_1744 | SAUSA300_RS09530 |  | 3.94E-05 | -2.3 | hypothetical protein |  |
| SAUSA300_2205 | SAUSA300_RS12155 | *rpsJ* | 6.16E-05 | -2.3 | 30S ribosomal protein S10 | [T][J][R] |
| SAUSA300_1561 | SAUSA300_RS08515 |  | 5.94E-10 | -2.3 | iron transporter | [P] |
| SAUSA300_1345 | SAUSA300_RS07335 | *asnC* | 1.02E-18 | -2.3 | asparagine--tRNA ligase | [O][J] |
| SAUSA300_0846 | SAUSA300_RS04570 |  | 1.08E-05 | -2.3 | sodium:proton antiporter |  |
| SAUSA300_0525 | SAUSA300_RS02810 | *rplL* | 0.001345 | -2.2 | 50S ribosomal protein L7/L12 | [J][R] |
| SAUSA300_2049 | SAUSA300_RS11280 | *thiD* | 0.000514 | -2.2 | hydroxymethylpyrimidine/phosphomethylpyrimidine kinase | [R] |
| SAUSA300_0040 | SAUSA300_RS00215 |  | 0.014799 | -2.2 | hypothetical protein |  |
| SAUSA300_0387 | SAUSA300_RS02065 | *pbuX* | 3.16E-14 | -2.2 | xanthine permease | [P][F] |
| SAUSA300_1565 | SAUSA300_RS08535 |  | 4.75E-09 | -2.2 | allophanate hydrolase | [E] |
| SAUSA300_1777 | SAUSA300_RS09725 |  | 0.018922 | -2.2 | tRNA-Phe |  |
| SAUSA300_2453 | SAUSA300_RS13605 |  | 1.17E-15 | -2.2 | lantibiotic ABC transporter ATP-binding protein | [R] |
| SAUSA300_2116 | SAUSA300_RS11655 |  | 0.000721 | -2.2 | tRNA-Lys |  |
| SAUSA300_2190 | SAUSA300_RS12080 | *rpsH* | 1.28E-07 | -2.2 | 30S ribosomal protein S8 | [J] |
| SAUSA300_0836 | SAUSA300_RS04520 | *dltB* | 6.12E-12 | -2.2 | D-alanyl-lipoteichoic acid biosynthesis protein DltB |  |
| SAUSA300_2636 | SAUSA300_RS14640 |  | 9.47E-08 | -2.2 | integrase | [R] |
| SAUSA300_0328 | SAUSA300_RS01745 |  | 2.99E-10 | -2.2 | lipoate--protein ligase A |  |
| SAUSA300_2249 | SAUSA300_RS12415 | *ssaA* | 0.00035 | -2.2 | CHAP domain-containing protein |  |
| SAUSA300_0502 | SAUSA300_RS02690 | *rrfB* | 2.19E-07 | -2.2 | 5S ribosomal RNA |  |
| SAUSA300_0266 | SAUSA300_RS01430 |  | 2.82E-05 | -2.2 | hypothetical protein |  |
| SAUSA300_1587 | SAUSA300_RS08650 | *hisS* | 2.30E-10 | -2.2 | histidine--tRNA ligase | [J][O] |
| SAUSA300_0409 | SAUSA300_RS02195 |  | 9.73E-11 | -2.2 | hypothetical protein |  |
| SAUSA300_1837 | SAUSA300_RS10035 | *rrfC* | 9.95E-07 | -2.2 | 5S ribosomal RNA |  |
| SAUSA300_1595 | SAUSA300_RS08690 | *tgt* | 1.53E-15 | -2.2 | queuine tRNA-ribosyltransferase | [J][O] |
| SAUSA300_2197 | SAUSA300_RS12115 | *rplP* | 0.000211 | -2.2 | 50S ribosomal protein L16 | [R][J] |
| SAUSA300_0457 | SAUSA300_RS02450 | *rrfA* | 1.48E-07 | -2.2 | 5S ribosomal RNA |  |
| SAUSA300_2189 | SAUSA300_RS12075 | *rplF* | 7.12E-06 | -2.2 | 50S ribosomal protein L6 | [R][J] |
| SAUSA300_1779 | SAUSA300_RS09735 |  | 0.000109 | -2.2 | tRNA-Met |  |
| SAUSA300_2489 | SAUSA300_RS13825 |  | 0.000244 | -2.2 | membrane protein | [P] |
| SAUSA300_0844 | SAUSA300_RS04560 |  | 2.23E-14 | -2.2 | NADH dehydrogenase |  |
| SAUSA300_1703 | SAUSA300_RS09300 |  | 1.81E-09 | -2.2 | rhodanese-like domain-containing protein |  |
| SAUSA300_2083 | SAUSA300_RS11465 |  | 2.81E-10 | -2.2 | acetyltransferase |  |
| SAUSA300_0500 | SAUSA300_RS02680 |  | 9.47E-08 | -2.2 | tRNA-Ile |  |
| SAUSA300_2463 | SAUSA300_RS13660 | *ddh* | 9.25E-10 | -2.2 | lactate dehydrogenase | [R] |
| SAUSA300_2172 | SAUSA300_RS11990 | *rplM* | 3.92E-05 | -2.2 | 50S ribosomal protein L13 | [J] |
| SAUSA300_1685 | SAUSA300_RS09210 |  | 1.14E-05 | -2.2 | hypothetical protein |  |
| SAUSA300_0566 | SAUSA300_RS03030 |  | 6.16E-11 | -2.2 | amino acid permease | [E][P] |
| SAUSA300_2592 | SAUSA300_RS14415 |  | 1.87E-05 | -2.2 | hypothetical protein |  |
| SAUSA300_1092 | SAUSA300_RS05920 | *pyrP* | 0.030781 | -2.2 | uracil permease | [F][P] |
| SAUSA300_2483 | SAUSA300_RS13790 |  | 7.79E-13 | -2.1 | hydroxymethylglutaryl-CoA reductase |  |
| SAUSA300_2048 | SAUSA300_RS11275 | *thiM* | 0.000121 | -2.1 | hydroxyethylthiazole kinase | [H] |
| SAUSA300_2370 | SAUSA300_RS13095 |  | 0.032585 | -2.1 | 8-amino-7-oxononanoate synthase |  |
| SAUSA300_0838 | SAUSA300_RS04530 | *dltD* | 3.95E-13 | -2.1 | D-alanyl-lipoteichoic acid biosynthesis protein DltD |  |
| SAUSA300_0497 | SAUSA300_RS02665 | *rrfG* | 1.04E-05 | -2.1 | 5S ribosomal RNA |  |
| SAUSA300_0139 | SAUSA300_RS00730 |  | 4.38E-05 | -2.1 | tetracycline efflux MFS transporter Tet(38) |  |
| SAUSA300_1506 | SAUSA300_RS08225 |  | 3.24E-12 | -2.1 | hypothetical protein |  |
| SAUSA300_2437 | SAUSA300_RS13510 | *sarT* | 0.001991 | -2.1 | transcriptional regulator | [K][R] |
| SAUSA300_1943 | SAUSA300_RS10660 |  | 1.44E-05 | -2.1 | HNH endonuclease | [S][R] |
| SAUSA300_2198 | SAUSA300_RS12120 | *rpsC* | 0.000294 | -2.1 | 30S ribosomal protein S3 | [T][J][R][K] |
| SAUSA300_1810 | SAUSA300_RS09905 |  | 0.000452 | -2.1 | transposase |  |
| SAUSA300_1757 | SAUSA300_RS09615 | *splB* | 0.023276 | -2.1 | serine protease SplB | [O] |
| SAUSA300_1020 | SAUSA300_RS05490 |  | 1.97E-08 | -2.1 | glycerophosphoryl diester phosphodiesterase | [I] |
| SAUSA300_0031 | SAUSA300_RS00160 |  | 2.82E-05 | -2.1 | hypothetical protein | [I] |
| SAUSA300_2403 | SAUSA300_RS13310 |  | 1.55E-08 | -2.1 | hypothetical protein |  |
| SAUSA300_2265 | SAUSA300_RS12515 |  | 1.66E-12 | -2.1 | alanine glycine permease | [E][P] |
| SAUSA300_1739 | SAUSA300_RS09510 |  | 6.84E-14 | -2.1 | calcium-binding protein |  |
| SAUSA300_0316 | SAUSA300_RS01685 |  | 0.000119 | -2.1 | N-acetylmannosamine kinase |  |
| SAUSA300_0456 | SAUSA300_RS02445 | *rrlA* | 0.002655 | -2.1 | 23S ribosomal RNA |  |
| SAUSA300_2122 | SAUSA300_RS11685 | *rrfE* | 3.33E-07 | -2.1 | 5S ribosomal RNA |  |
| SAUSA300_0960 | SAUSA300_RS05160 | *qoxD* | 0.002519 | -2.1 | quinol oxidase subunit 4 |  |
| SAUSA300_2182 | SAUSA300_RS12040 | *infA* | 1.10E-05 | -2.1 | translation initiation factor IF-1 | [T][J] |
| SAUSA300_2200 | SAUSA300_RS12130 | *rpsS* | 0.00127 | -2.1 | 30S ribosomal protein S19 | [T][J] |
| SAUSA300_0183 | SAUSA300_RS00960 |  | 1.55E-09 | -2.1 | hypothetical protein |  |
| SAUSA300_2184 | SAUSA300_RS12050 | *secY* | 4.45E-05 | -2.1 | protein translocase subunit SecY | [P] [U] |
| SAUSA300_1525 | SAUSA300_RS08320 | *glyS* | 1.80E-14 | -2.1 | glycine--tRNA ligase | [J] |
| SAUSA300_0353 | SAUSA300_RS01870 |  | 6.80E-05 | -2.1 | membrane protein |  |
| SAUSA300_0362 | SAUSA300_RS01920 |  | 1.29E-05 | -2.1 | mechanosensitive ion channel protein MscS | [P] |
| SAUSA300_1507 | SAUSA300_RS08230 | *glk* | 1.55E-11 | -2 | glucokinase | [O] |
| SAUSA300_2320 | SAUSA300_RS12820 |  | 1.65E-05 | -2 | membrane protein |  |
| SAUSA300_0501 | SAUSA300_RS02685 | *rrlB* | 0.00199 | -2 | 23S ribosomal RNA |  |
| SAUSA300_0685 | SAUSA300_RS03675 | *fruA* | 1.50E-06 | -2 | PTS fructose transporter subunit IIC | [P][M] |
| SAUSA300_0136 | SAUSA300_RS00710 |  | 8.42E-06 | -2 | hypothetical protein |  |
| SAUSA300_0589 | SAUSA300_RS03165 |  | 1.61E-08 | -2 | oxidoreductase ion channel protein IolS | [R][Q] |
| SAUSA300_2287 | SAUSA300_RS12640 |  | 3.05E-09 | -2 | sodium ABC transporter permease |  |
| SAUSA300_1830 | SAUSA300_RS10000 |  | 1.32E-05 | -2 | tRNA-Arg |  |
| SAUSA300_0330 | SAUSA300_RS01755 | *ulaA* | 2.13E-05 | -2 | PTS ascorbate transporter subunit IIC |  |
| SAUSA300_2180 | SAUSA300_RS12030 | *rpsM* | 5.11E-05 | -2 | 30S ribosomal protein S13 | [T][J][R] |
| SAUSA300_0637 | SAUSA300_RS03415 |  | 2.16E-05 | -2 | dihydroxyacetone kinase subunit DhaL |  |
| SAUSA300_1234 | SAUSA300_RS06690 | *rpsN* | 0.01154 | -2 | 30S ribosomal protein S14 | [R][J] |
| SAUSA300_2298 | SAUSA300_RS12695 |  | 0.000319 | -2 | multidrug MFS transporter | [P] |
| SAUSA300_0953 | SAUSA300_RS05120 |  | 0.00459 | -2 | acyltransferase |  |
| SAUSA300_0067 | SAUSA300_RS00340 |  | 1.71E-12 | -2 | universal stress protein | [T] |
| SAUSA300_1611 | SAUSA300_RS08780 | *valS* | 1.50E-13 | -2 | valine--tRNA ligase | [O][R] |
| SAUSA300_1149 | SAUSA300_RS06220 | *rpsB* | 0.000304 | -2 | 30S ribosomal protein S2 | [R][J] |
| SAUSA300_2476 | SAUSA300_RS13740 | *ptsG* | 2.01E-06 | -2 | PTS glucoside EIICBA component | [P] [M] |
| SAUSA300_0290 | SAUSA300_RS01550 |  | 0.012206 | -2 | hypothetical protein |  |
| SAUSA300_2329 | SAUSA300_RS12870 | *gltT* | 7.25E-09 | -2 | proton/sodium-glutamate symport protein GltT | [E][H] |
| SAUSA300_0359 | SAUSA300_RS01905 | ***metC2*** | 0.00546 | -2 | cystathionine beta-lyase |  |
| SAUSA300_1947 | SAUSA300_RS10680 |  | 0.000351 | -2 | hypothetical protein |  |
| SAUSA300_0982 | SAUSA300_RS05285 |  | 3.00E-12 | -2 | hypothetical protein |  |
| SAUSA300_1505 | SAUSA300_RS08220 |  | 3.66E-12 | -2 | hydroxyacylglutathione hydrolase | [Q] |
| SAUSA300_0386 | SAUSA300_RS02060 | *xpt* | 5.26E-10 | -2 | xanthine phosphoribosyltransferase | [H] |

^a^genes in bold indicate pan-gene names; ^b^COG: Clusters of Orthologous groups; [C] Energy production and conversion; [E] Amino acid transport and metabolism; [F] Nucleotide transport and metabolism; [G] Carbohydrate transport and metabolism; [H] Coenzyme transport and metabolism; [I] Lipid transport and metabolism; [J] Translation, ribosomal structure and biogenesis; [K] Transcription; [L] Replication, recombination and repair; [M] Cell wall/membrane/envelope biogenesis; [O] Posttranslational modification, protein turnover, chaperones; [P] Inorganic ion transport and metabolism; [Q] Secondary metabolites biosynthesis, transport and catabolism; [R] General function prediction only; [S] Function unknown; [T] Signal transduction mechanisms; [U] Intracellular trafficking, secretion, and vesicular transport; [V] Defense mechanisms; [X] No prediction. In yellow are highlighted the genes referred in the manuscript.

**Table S4.** **Validation of relative gene expression of selected genes altered in the RNA-Seq data. Transcript abundance evaluated by qRT-PCR (ratio +NO/-NO) for *S. aureus* grown on CDM containing galactose, glucose and with no glycolytic carbon source, and analyzed 3h after the NO pulse**

| **Gene** | **Locus_tag** | **No glycolytic carbon source** | **Glucose** | **Galactose** |
| --- | --- | --- | --- | --- |
| *alsS* | SAUSA300_2166 | 0.5±0.0 | 1.9±0.4 | 6.4±0.6 |
| *budA* | SAUSA300_2165 | 0.5±0.0 | 2.6±1.2 | 14.6±1.1 |
| *cap5A* | SAUSA300_0152 | 0.5±0.2 | 0.5±0.0 | 8.4±0.6 |
| *sdrC* | SAUSA300_0546 | 0.3±0.0 | 0.2±0.0 | 2.2±0.0 |
| *thrA* | SAUSA300_1225 | 0.5±0.1 | 0.2±0.0 | 75.3±1.3 |
| *fadE* | SAUSA300_0228 | 0.3±0.1 | 0.2±0.0 | 9.0±0.2 |
| *zwf* | SAUSA300_1454 | 3.2±1.2 | 5.5±1.9 | 2.6±0.3 |
| *fbp* | SAUSA300_2455 | 2.4±1.1 | 2.2±1.0 | 2.5±0.9 |

**Table S5. Gene Set Enrichment Analysis (GSEA) for genes with fold ≥ 2 and ≤ 0.5 in *S. aureus* cells grown on CDM-galactose and exposed to NO stress (250 µM Spermine NONOate), using a cut-off value for the adjusted *p*-values of 0.05**

| **Database^a^** | **Class_ID^b^** | **Class Description^c^** | **Class Size^d^** | **Significance^e^** |
| --- | --- | --- | --- | --- |
| **Tophits (upregulated genes)** | |  |  |  |
| KEGG | [260](http://www.genome.jp/kegg-bin/show_pathway?saa00260) | Glycine, serine and threonine metabolism | 27 | [0.0027 (6)](http://www.genome.jp/kegg-bin/show_pathway?saa00260+SAUSA300_RS06640+SAUSA300_RS06645+SAUSA300_RS06650+SAUSA300_RS06655+SAUSA300_RS06995+SAUSA300_RS14135) |
| KEGG | [270](http://www.genome.jp/kegg-bin/show_pathway?saa00270) | Cysteine and methionine metabolism | 21 | [0.0040 (5)](http://www.genome.jp/kegg-bin/show_pathway?saa00270+SAUSA300_RS06640+SAUSA300_RS06645+SAUSA300_RS06995+SAUSA300_RS08765+SAUSA300_RS14190) |
| KEGG | [30](http://www.genome.jp/kegg-bin/show_pathway?saa00030) | Pentose phosphate pathway | 20 | [0.0171 (4)](http://www.genome.jp/kegg-bin/show_pathway?saa00030+SAUSA300_RS01405+SAUSA300_RS07940+SAUSA300_RS09425+SAUSA300_RS13550) |
| KEGG | [300](http://www.genome.jp/kegg-bin/show_pathway?saa00300) | Lysine biosynthesis | 10 | [0.0171 (3)](http://www.genome.jp/kegg-bin/show_pathway?saa00300+SAUSA300_RS06640+SAUSA300_RS06645+SAUSA300_RS06995) |
| KEGG | [790](http://www.genome.jp/kegg-bin/show_pathway?saa00790) | Folate biosynthesis | 13 | [0.0305 (3)](http://www.genome.jp/kegg-bin/show_pathway?saa00790+SAUSA300_RS02940+SAUSA300_RS03730+SAUSA300_RS03740) |
| KEGG | [380](http://www.genome.jp/kegg-bin/show_pathway?saa00380) | Tryptophan metabolism | 6 | [0.0359 (2)](http://www.genome.jp/kegg-bin/show_pathway?saa00380+SAUSA300_RS06680+SAUSA300_RS08765) |
| KEGG | [627](http://www.genome.jp/kegg-bin/show_pathway?saa00627) | Aminobenzoate degradation | 6 | [0.0359 (2)](http://www.genome.jp/kegg-bin/show_pathway?saa00627+SAUSA300_RS01200+SAUSA300_RS08765) |
| KEGG | [630](http://www.genome.jp/kegg-bin/show_pathway?saa00630) | Glyoxylate and dicarboxylate metabolism | 6 | [0.0359 (2)](http://www.genome.jp/kegg-bin/show_pathway?saa00630+SAUSA300_RS06680+SAUSA300_RS12475) |
| KEGG | [660](http://www.genome.jp/kegg-bin/show_pathway?saa00660) | C5-Branched dibasic acid metabolism | 7 | [0.0394 (2)](http://www.genome.jp/kegg-bin/show_pathway?saa00660+SAUSA300_RS11935+SAUSA300_RS11940) |
| KEGG | [360](http://www.genome.jp/kegg-bin/show_pathway?saa00360) | Phenylalanine metabolism | 7 | [0.0394 (2)](http://www.genome.jp/kegg-bin/show_pathway?saa00360+SAUSA300_RS01200+SAUSA300_RS08765) |
| COG | [E](http://ecoliwiki.net/colipedia/index.php/Clusters_of_Orthologous_Groups_(COGs)E) | Amino acid transport and metabolism | 86 | [3.0e-10 (11)](http://genome2d.molgenrug.nl/genome2d_results/GSEA_pro/193.136.177.1i6fcn62stkevgvfg07il3k7hj187/GSEA_Pro.single_list.E.txt) |
| COG | [U](http://ecoliwiki.net/colipedia/index.php/Clusters_of_Orthologous_Groups_(COGs)U) | Intracellular trafficking, secretion, and vesicular transport | 16 | [8.4e-07 ( 5)](http://genome2d.molgenrug.nl/genome2d_results/GSEA_pro/193.136.177.1i6fcn62stkevgvfg07il3k7hj187/GSEA_Pro.single_list.U.txt) |
| COG | [S](http://ecoliwiki.net/colipedia/index.php/Clusters_of_Orthologous_Groups_(COGs)S) | Function unknown | 31 | [8.8e-07 ( 6)](http://genome2d.molgenrug.nl/genome2d_results/GSEA_pro/193.136.177.1i6fcn62stkevgvfg07il3k7hj187/GSEA_Pro.single_list.S.txt) |
| COG | [L](http://ecoliwiki.net/colipedia/index.php/Clusters_of_Orthologous_Groups_(COGs)L) | Replication, recombination and repair | 85 | [5.7e-09 (10)](http://genome2d.molgenrug.nl/genome2d_results/GSEA_pro/193.136.177.1i6fcn62stkevgvfg07il3k7hj187/GSEA_Pro.single_list.L.txt) |
| COG | [G](http://ecoliwiki.net/colipedia/index.php/Clusters_of_Orthologous_Groups_(COGs)G) | Carbohydrate transport and metabolism | 27 | [1.1e-05 ( 5)](http://genome2d.molgenrug.nl/genome2d_results/GSEA_pro/193.136.177.1i6fcn62stkevgvfg07il3k7hj187/GSEA_Pro.single_list.G.txt) |
| COG | [H](http://ecoliwiki.net/colipedia/index.php/Clusters_of_Orthologous_Groups_(COGs)H) | Coenzyme transport and metabolism | 34 | [5.9e-04 ( 4)](http://genome2d.molgenrug.nl/genome2d_results/GSEA_pro/193.136.177.1i6fcn62stkevgvfg07il3k7hj187/GSEA_Pro.single_list.H.txt) |
| COG | [C](http://ecoliwiki.net/colipedia/index.php/Clusters_of_Orthologous_Groups_(COGs)C) | Energy production and conversion | 36 | [6.7e-04 ( 4)](http://genome2d.molgenrug.nl/genome2d_results/GSEA_pro/193.136.177.1i6fcn62stkevgvfg07il3k7hj187/GSEA_Pro.single_list.C.txt) |
| COG | [Q](http://ecoliwiki.net/colipedia/index.php/Clusters_of_Orthologous_Groups_(COGs)Q) | Secondary metabolites biosynthesis, transport and catabolism | 21 | [1.7e-03 ( 3)](http://genome2d.molgenrug.nl/genome2d_results/GSEA_pro/193.136.177.1i6fcn62stkevgvfg07il3k7hj187/GSEA_Pro.single_list.Q.txt) |
| GO | [GO:0009432](http://www.ebi.ac.uk/QuickGO/GTerm?id=GO:0009432) | SOS response | 3 | [8.0e-04 ( 3)](http://genome2d.molgenrug.nl/genome2d_results/GSEA_pro/193.136.177.1i6fcn62stkevgvfg07il3k7hj187/GSEA_Pro.single_list.GO:0009432.txt) |
| GO | [GO:0009088](http://www.ebi.ac.uk/QuickGO/GTerm?id=GO:0009088) | threonine biosynthetic process | 2 | [6.5e-03 ( 2)](http://genome2d.molgenrug.nl/genome2d_results/GSEA_pro/193.136.177.1i6fcn62stkevgvfg07il3k7hj187/GSEA_Pro.single_list.GO:0009088.txt) |
| GO | [GO:0006879](http://www.ebi.ac.uk/QuickGO/GTerm?id=GO:0006879) | cellular iron ion homeostasis | 2 | [6.5e-03 ( 2)](http://genome2d.molgenrug.nl/genome2d_results/GSEA_pro/193.136.177.1i6fcn62stkevgvfg07il3k7hj187/GSEA_Pro.single_list.GO:0006879.txt) |
| GO | [GO:0008199](http://www.ebi.ac.uk/QuickGO/GTerm?id=GO:0008199) | ferric iron binding | 2 | [6.5e-03 ( 2)](http://genome2d.molgenrug.nl/genome2d_results/GSEA_pro/193.136.177.1i6fcn62stkevgvfg07il3k7hj187/GSEA_Pro.single_list.GO:0008199.txt) |
| GO | [GO:0015031](http://www.ebi.ac.uk/QuickGO/GTerm?id=GO:0015031) | protein transport | 8 | [1.0e-03 ( 4)](http://genome2d.molgenrug.nl/genome2d_results/GSEA_pro/193.136.177.1i6fcn62stkevgvfg07il3k7hj187/GSEA_Pro.single_list.GO:0015031.txt) |
| GO | [GO:0009055](http://www.ebi.ac.uk/QuickGO/GTerm?id=GO:0009055) | electron carrier activity | 6 | [5.5e-03 ( 3)](http://genome2d.molgenrug.nl/genome2d_results/GSEA_pro/193.136.177.1i6fcn62stkevgvfg07il3k7hj187/GSEA_Pro.single_list.GO:0009055.txt) |
| GO | [GO:0006537](http://www.ebi.ac.uk/QuickGO/GTerm?id=GO:0006537) | glutamate biosynthetic process | 3 | [1.1e-02 ( 2)](http://genome2d.molgenrug.nl/genome2d_results/GSEA_pro/193.136.177.1i6fcn62stkevgvfg07il3k7hj187/GSEA_Pro.single_list.GO:0006537.txt) |
| GO | [GO:0009381](http://www.ebi.ac.uk/QuickGO/GTerm?id=GO:0009381) | excinuclease ABC activity | 3 | [1.1e-02 ( 2)](http://genome2d.molgenrug.nl/genome2d_results/GSEA_pro/193.136.177.1i6fcn62stkevgvfg07il3k7hj187/GSEA_Pro.single_list.GO:0009381.txt) |
| GO | [GO:0030151](http://www.ebi.ac.uk/QuickGO/GTerm?id=GO:0030151) | molybdenum ion binding | 3 | [1.1e-02 ( 2)](http://genome2d.molgenrug.nl/genome2d_results/GSEA_pro/193.136.177.1i6fcn62stkevgvfg07il3k7hj187/GSEA_Pro.single_list.GO:0030151.txt) |
| GO | [GO:0006289](http://www.ebi.ac.uk/QuickGO/GTerm?id=GO:0006289) | nucleotide-excision repair | 3 | [1.1e-02 ( 2)](http://genome2d.molgenrug.nl/genome2d_results/GSEA_pro/193.136.177.1i6fcn62stkevgvfg07il3k7hj187/GSEA_Pro.single_list.GO:0006289.txt) |
| GO | [GO:0006520](http://www.ebi.ac.uk/QuickGO/GTerm?id=GO:0006520) | cellular amino acid metabolic process | 11 | [1.6e-02 ( 3)](http://genome2d.molgenrug.nl/genome2d_results/GSEA_pro/193.136.177.1i6fcn62stkevgvfg07il3k7hj187/GSEA_Pro.single_list.GO:0006520.txt) |
| GO | [GO:0008652](http://www.ebi.ac.uk/QuickGO/GTerm?id=GO:0008652) | cellular amino acid biosynthetic process | 5 | [3.0e-02 ( 2)](http://genome2d.molgenrug.nl/genome2d_results/GSEA_pro/193.136.177.1i6fcn62stkevgvfg07il3k7hj187/GSEA_Pro.single_list.GO:0008652.txt) |
| GO | [GO:0008152](http://www.ebi.ac.uk/QuickGO/GTerm?id=GO:0008152) | metabolic process | 127 | [9.3e-04 (14)](http://genome2d.molgenrug.nl/genome2d_results/GSEA_pro/193.136.177.1i6fcn62stkevgvfg07il3k7hj187/GSEA_Pro.single_list.GO:0008152.txt) |
| GO | [GO:0009089](http://www.ebi.ac.uk/QuickGO/GTerm?id=GO:0009089) | lysine biosynthetic process via diaminopimelate | 7 | [4.6e-02 ( 2)](http://genome2d.molgenrug.nl/genome2d_results/GSEA_pro/193.136.177.1i6fcn62stkevgvfg07il3k7hj187/GSEA_Pro.single_list.GO:0009089.txt) |
| GO | [GO:0006284](http://www.ebi.ac.uk/QuickGO/GTerm?id=GO:0006284) | base-excision repair | 7 | [4.6e-02 ( 2)](http://genome2d.molgenrug.nl/genome2d_results/GSEA_pro/193.136.177.1i6fcn62stkevgvfg07il3k7hj187/GSEA_Pro.single_list.GO:0006284.txt) |
| GO | [GO:0006807](http://www.ebi.ac.uk/QuickGO/GTerm?id=GO:0006807) | nitrogen compound metabolic process | 9 | [1.1e-02 ( 3)](http://genome2d.molgenrug.nl/genome2d_results/GSEA_pro/193.136.177.1i6fcn62stkevgvfg07il3k7hj187/GSEA_Pro.single_list.GO:0006807.txt) |
| GO | [GO:0003824](http://www.ebi.ac.uk/QuickGO/GTerm?id=GO:0003824) | catalytic activity | 213 | [2.1e-05 (22)](http://genome2d.molgenrug.nl/genome2d_results/GSEA_pro/193.136.177.1i6fcn62stkevgvfg07il3k7hj187/GSEA_Pro.single_list.GO:0003824.txt) |
| GO | [GO:0006281](http://www.ebi.ac.uk/QuickGO/GTerm?id=GO:0006281) | DNA repair | 30 | [4.2e-03 ( 6)](http://genome2d.molgenrug.nl/genome2d_results/GSEA_pro/193.136.177.1i6fcn62stkevgvfg07il3k7hj187/GSEA_Pro.single_list.GO:0006281.txt) |
| GO | [GO:0009405](http://www.ebi.ac.uk/QuickGO/GTerm?id=GO:0009405) | pathogenesis | 37 | [6.8e-03 ( 6)](http://genome2d.molgenrug.nl/genome2d_results/GSEA_pro/193.136.177.1i6fcn62stkevgvfg07il3k7hj187/GSEA_Pro.single_list.GO:0009405.txt) |
| GO | [GO:0015035](http://www.ebi.ac.uk/QuickGO/GTerm?id=GO:0015035) | protein disulfide oxidoreductase activity | 6 | [4.2e-02 ( 2)](http://genome2d.molgenrug.nl/genome2d_results/GSEA_pro/193.136.177.1i6fcn62stkevgvfg07il3k7hj187/GSEA_Pro.single_list.GO:0015035.txt) |
| GO | [GO:0016491](http://www.ebi.ac.uk/QuickGO/GTerm?id=GO:0016491) | oxidoreductase activity | 103 | [5.0e-03 (11)](http://genome2d.molgenrug.nl/genome2d_results/GSEA_pro/193.136.177.1i6fcn62stkevgvfg07il3k7hj187/GSEA_Pro.single_list.GO:0016491.txt) |
| GO | [GO:0016810](http://www.ebi.ac.uk/QuickGO/GTerm?id=GO:0016810) | hydrolase activity, acting on carbon-nitrogen (but not peptide) bonds | 7 | [4.6e-02 ( 2)](http://genome2d.molgenrug.nl/genome2d_results/GSEA_pro/193.136.177.1i6fcn62stkevgvfg07il3k7hj187/GSEA_Pro.single_list.GO:0016810.txt) |
| GO | [GO:0030976](http://www.ebi.ac.uk/QuickGO/GTerm?id=GO:0030976) | thiamine pyrophosphate binding | 7 | [4.6e-02 ( 2)](http://genome2d.molgenrug.nl/genome2d_results/GSEA_pro/193.136.177.1i6fcn62stkevgvfg07il3k7hj187/GSEA_Pro.single_list.GO:0030976.txt) |
| GO | [GO:0050661](http://www.ebi.ac.uk/QuickGO/GTerm?id=GO:0050661) | NADP binding | 13 | [2.5e-02 ( 3)](http://genome2d.molgenrug.nl/genome2d_results/GSEA_pro/193.136.177.1i6fcn62stkevgvfg07il3k7hj187/GSEA_Pro.single_list.GO:0050661.txt) |
| GO | [GO:0051536](http://www.ebi.ac.uk/QuickGO/GTerm?id=GO:0051536) | iron-sulfur cluster binding | 19 | [1.1e-02 ( 4)](http://genome2d.molgenrug.nl/genome2d_results/GSEA_pro/193.136.177.1i6fcn62stkevgvfg07il3k7hj187/GSEA_Pro.single_list.GO:0051536.txt) |
| GO | [GO:0055114](http://www.ebi.ac.uk/QuickGO/GTerm?id=GO:0055114) | oxidation-reduction process | 169 | [8.3e-08 (23)](http://genome2d.molgenrug.nl/genome2d_results/GSEA_pro/193.136.177.1i6fcn62stkevgvfg07il3k7hj187/GSEA_Pro.single_list.GO:0055114.txt) |
| IPR | [IPR007595](http://www.ebi.ac.uk/interpro/entry/IPR007595) | Protein of unknown function DUF576 | 16 | [9.8e-08 ( 8)](http://genome2d.molgenrug.nl/genome2d_results/GSEA_pro/193.136.177.1i6fcn62stkevgvfg07il3k7hj187/GSEA_Pro.single_list.IPR007595.txt) |
| IPR | [IPR008331](http://www.ebi.ac.uk/interpro/entry/IPR008331) | Ferritin/DPS protein domain | 2 | [3.5e-03 ( 2)](http://genome2d.molgenrug.nl/genome2d_results/GSEA_pro/193.136.177.1i6fcn62stkevgvfg07il3k7hj187/GSEA_Pro.single_list.IPR008331.txt) |
| IPR | [IPR012347](http://www.ebi.ac.uk/interpro/entry/IPR012347) | Ferritin-related | 2 | [3.5e-03 ( 2)](http://genome2d.molgenrug.nl/genome2d_results/GSEA_pro/193.136.177.1i6fcn62stkevgvfg07il3k7hj187/GSEA_Pro.single_list.IPR012347.txt) |
| IPR | [IPR011439](http://www.ebi.ac.uk/interpro/entry/IPR011439) | Domain of unknown function DUF1542 | 2 | [3.5e-03 ( 2)](http://genome2d.molgenrug.nl/genome2d_results/GSEA_pro/193.136.177.1i6fcn62stkevgvfg07il3k7hj187/GSEA_Pro.single_list.IPR011439.txt) |
| IPR | [IPR005119](http://www.ebi.ac.uk/interpro/entry/IPR005119) | LysR, substrate-binding | 6 | [2.9e-03 ( 3)](http://genome2d.molgenrug.nl/genome2d_results/GSEA_pro/193.136.177.1i6fcn62stkevgvfg07il3k7hj187/GSEA_Pro.single_list.IPR005119.txt) |
| IPR | [IPR000847](http://www.ebi.ac.uk/interpro/entry/IPR000847) | Transcription regulator HTH, LysR | 7 | [3.5e-03 ( 3)](http://genome2d.molgenrug.nl/genome2d_results/GSEA_pro/193.136.177.1i6fcn62stkevgvfg07il3k7hj187/GSEA_Pro.single_list.IPR000847.txt) |
| IPR | [IPR013783](http://www.ebi.ac.uk/interpro/entry/IPR013783) | Immunoglobulin-like fold | 3 | [7.3e-03 ( 2)](http://genome2d.molgenrug.nl/genome2d_results/GSEA_pro/193.136.177.1i6fcn62stkevgvfg07il3k7hj187/GSEA_Pro.single_list.IPR013783.txt) |
| IPR | [IPR005877](http://www.ebi.ac.uk/interpro/entry/IPR005877) | YSIRK Gram-positive signal peptide | 16 | [5.3e-05 ( 6)](http://genome2d.molgenrug.nl/genome2d_results/GSEA_pro/193.136.177.1i6fcn62stkevgvfg07il3k7hj187/GSEA_Pro.single_list.IPR005877.txt) |
| IPR | [IPR008454](http://www.ebi.ac.uk/interpro/entry/IPR008454) | Collagen-binding surface protein Cna-like, B-type domain | 3 | [7.3e-03 ( 2)](http://genome2d.molgenrug.nl/genome2d_results/GSEA_pro/193.136.177.1i6fcn62stkevgvfg07il3k7hj187/GSEA_Pro.single_list.IPR008454.txt) |
| IPR | [IPR009078](http://www.ebi.ac.uk/interpro/entry/IPR009078) | Ferritin-like superfamily | 3 | [7.3e-03 ( 2)](http://genome2d.molgenrug.nl/genome2d_results/GSEA_pro/193.136.177.1i6fcn62stkevgvfg07il3k7hj187/GSEA_Pro.single_list.IPR009078.txt) |
| IPR | [IPR018392](http://www.ebi.ac.uk/interpro/entry/IPR018392) | LysM domain | 6 | [2.9e-03 ( 3)](http://genome2d.molgenrug.nl/genome2d_results/GSEA_pro/193.136.177.1i6fcn62stkevgvfg07il3k7hj187/GSEA_Pro.single_list.IPR018392.txt) |
| IPR | [IPR029039](http://www.ebi.ac.uk/interpro/entry/IPR029039) | Flavoprotein-like | 7 | [3.5e-03 ( 3)](http://genome2d.molgenrug.nl/genome2d_results/GSEA_pro/193.136.177.1i6fcn62stkevgvfg07il3k7hj187/GSEA_Pro.single_list.IPR029039.txt) |
| IPR | [IPR001650](http://www.ebi.ac.uk/interpro/entry/IPR001650) | Helicase, C-terminal | 10 | [3.9e-02 ( 2)](http://genome2d.molgenrug.nl/genome2d_results/GSEA_pro/193.136.177.1i6fcn62stkevgvfg07il3k7hj187/GSEA_Pro.single_list.IPR001650.txt) |
| IPR | [IPR000873](http://www.ebi.ac.uk/interpro/entry/IPR000873) | AMP-dependent synthetase/ligase | 7 | [2.1e-02 ( 2)](http://genome2d.molgenrug.nl/genome2d_results/GSEA_pro/193.136.177.1i6fcn62stkevgvfg07il3k7hj187/GSEA_Pro.single_list.IPR000873.txt) |
| IPR | [IPR000150](http://www.ebi.ac.uk/interpro/entry/IPR000150) | Cof protein | 6 | [2.1e-02 ( 2)](http://genome2d.molgenrug.nl/genome2d_results/GSEA_pro/193.136.177.1i6fcn62stkevgvfg07il3k7hj187/GSEA_Pro.single_list.IPR000150.txt) |
| IPR | [IPR000182](http://www.ebi.ac.uk/interpro/entry/IPR000182) | GNAT domain | 20 | [2.2e-02 ( 3)](http://genome2d.molgenrug.nl/genome2d_results/GSEA_pro/193.136.177.1i6fcn62stkevgvfg07il3k7hj187/GSEA_Pro.single_list.IPR000182.txt) |
| IPR | [IPR001647](http://www.ebi.ac.uk/interpro/entry/IPR001647) | DNA-binding HTH domain, TetR-type | 7 | [2.1e-02 ( 2)](http://genome2d.molgenrug.nl/genome2d_results/GSEA_pro/193.136.177.1i6fcn62stkevgvfg07il3k7hj187/GSEA_Pro.single_list.IPR001647.txt) |
| IPR | [IPR001867](http://www.ebi.ac.uk/interpro/entry/IPR001867) | Signal transduction response regulator, C-terminal | 9 | [3.2e-02 ( 2)](http://genome2d.molgenrug.nl/genome2d_results/GSEA_pro/193.136.177.1i6fcn62stkevgvfg07il3k7hj187/GSEA_Pro.single_list.IPR001867.txt) |
| IPR | [IPR001926](http://www.ebi.ac.uk/interpro/entry/IPR001926) | Tryptophan synthase beta subunit-like PLP-dependent enzyme | 7 | [2.1e-02 ( 2)](http://genome2d.molgenrug.nl/genome2d_results/GSEA_pro/193.136.177.1i6fcn62stkevgvfg07il3k7hj187/GSEA_Pro.single_list.IPR001926.txt) |
| IPR | [IPR006123](http://www.ebi.ac.uk/interpro/entry/IPR006123) | Staphylococcal/Streptococcal toxin, beta-grasp domain | 18 | [2.1e-02 ( 3)](http://genome2d.molgenrug.nl/genome2d_results/GSEA_pro/193.136.177.1i6fcn62stkevgvfg07il3k7hj187/GSEA_Pro.single_list.IPR006123.txt) |
| IPR | [IPR006379](http://www.ebi.ac.uk/interpro/entry/IPR006379) | HAD-superfamily hydrolase, subfamily IIB | 7 | [2.1e-02 ( 2)](http://genome2d.molgenrug.nl/genome2d_results/GSEA_pro/193.136.177.1i6fcn62stkevgvfg07il3k7hj187/GSEA_Pro.single_list.IPR006379.txt) |
| IPR | [IPR006728](http://www.ebi.ac.uk/interpro/entry/IPR006728) | Protein of unknown function DUF600 | 9 | [3.2e-02 ( 2)](http://genome2d.molgenrug.nl/genome2d_results/GSEA_pro/193.136.177.1i6fcn62stkevgvfg07il3k7hj187/GSEA_Pro.single_list.IPR006728.txt) |
| IPR | [IPR007921](http://www.ebi.ac.uk/interpro/entry/IPR007921) | CHAP domain | 13 | [2.9e-03 ( 4)](http://genome2d.molgenrug.nl/genome2d_results/GSEA_pro/193.136.177.1i6fcn62stkevgvfg07il3k7hj187/GSEA_Pro.single_list.IPR007921.txt) |
| IPR | [IPR008966](http://www.ebi.ac.uk/interpro/entry/IPR008966) | Adhesion domain | 7 | [2.1e-02 ( 2)](http://genome2d.molgenrug.nl/genome2d_results/GSEA_pro/193.136.177.1i6fcn62stkevgvfg07il3k7hj187/GSEA_Pro.single_list.IPR008966.txt) |
| IPR | [IPR008992](http://www.ebi.ac.uk/interpro/entry/IPR008992) | Enterotoxin | 14 | [1.5e-02 ( 3)](http://genome2d.molgenrug.nl/genome2d_results/GSEA_pro/193.136.177.1i6fcn62stkevgvfg07il3k7hj187/GSEA_Pro.single_list.IPR008992.txt) |
| IPR | [IPR011252](http://www.ebi.ac.uk/interpro/entry/IPR011252) | Fibrogen-binding domain 1 | 7 | [2.1e-02 ( 2)](http://genome2d.molgenrug.nl/genome2d_results/GSEA_pro/193.136.177.1i6fcn62stkevgvfg07il3k7hj187/GSEA_Pro.single_list.IPR011252.txt) |
| IPR | [IPR011257](http://www.ebi.ac.uk/interpro/entry/IPR011257) | DNA glycosylase | 4 | [1.3e-02 ( 2)](http://genome2d.molgenrug.nl/genome2d_results/GSEA_pro/193.136.177.1i6fcn62stkevgvfg07il3k7hj187/GSEA_Pro.single_list.IPR011257.txt) |
| IPR | [IPR011266](http://www.ebi.ac.uk/interpro/entry/IPR011266) | Fibrinogen-binding domain 2 | 7 | [2.1e-02 ( 2)](http://genome2d.molgenrug.nl/genome2d_results/GSEA_pro/193.136.177.1i6fcn62stkevgvfg07il3k7hj187/GSEA_Pro.single_list.IPR011266.txt) |
| IPR | [IPR011766](http://www.ebi.ac.uk/interpro/entry/IPR011766) | Thiamine pyrophosphate enzyme, C-terminal TPP-binding | 6 | [2.1e-02 ( 2)](http://genome2d.molgenrug.nl/genome2d_results/GSEA_pro/193.136.177.1i6fcn62stkevgvfg07il3k7hj187/GSEA_Pro.single_list.IPR011766.txt) |
| IPR | [IPR011991](http://www.ebi.ac.uk/interpro/entry/IPR011991) | Winged helix-turn-helix DNA-binding domain | 100 | [2.9e-03 (10)](http://genome2d.molgenrug.nl/genome2d_results/GSEA_pro/193.136.177.1i6fcn62stkevgvfg07il3k7hj187/GSEA_Pro.single_list.IPR011991.txt) |
| IPR | [IPR012336](http://www.ebi.ac.uk/interpro/entry/IPR012336) | Thioredoxin-like fold | 19 | [2.1e-02 ( 3)](http://genome2d.molgenrug.nl/genome2d_results/GSEA_pro/193.136.177.1i6fcn62stkevgvfg07il3k7hj187/GSEA_Pro.single_list.IPR012336.txt) |
| IPR | [IPR013307](http://www.ebi.ac.uk/interpro/entry/IPR013307) | Superantigen, staphylococcal/streptococcal toxin, bacterial | 16 | [2.1e-02 ( 3)](http://genome2d.molgenrug.nl/genome2d_results/GSEA_pro/193.136.177.1i6fcn62stkevgvfg07il3k7hj187/GSEA_Pro.single_list.IPR013307.txt) |
| IPR | [IPR015893](http://www.ebi.ac.uk/interpro/entry/IPR015893) | Tetracycline transcriptional regulator, TetR-like, C-terminal | 7 | [2.1e-02 ( 2)](http://genome2d.molgenrug.nl/genome2d_results/GSEA_pro/193.136.177.1i6fcn62stkevgvfg07il3k7hj187/GSEA_Pro.single_list.IPR015893.txt) |
| IPR | [IPR016039](http://www.ebi.ac.uk/interpro/entry/IPR016039) | Thiolase-like | 7 | [2.1e-02 ( 2)](http://genome2d.molgenrug.nl/genome2d_results/GSEA_pro/193.136.177.1i6fcn62stkevgvfg07il3k7hj187/GSEA_Pro.single_list.IPR016039.txt) |
| IPR | [IPR016040](http://www.ebi.ac.uk/interpro/entry/IPR016040) | NAD(P)-binding domain | 78 | [3.8e-03 ( 8)](http://genome2d.molgenrug.nl/genome2d_results/GSEA_pro/193.136.177.1i6fcn62stkevgvfg07il3k7hj187/GSEA_Pro.single_list.IPR016040.txt) |
| IPR | [IPR016091](http://www.ebi.ac.uk/interpro/entry/IPR016091) | Superantigen toxin, C-terminal | 20 | [6.8e-03 ( 4)](http://genome2d.molgenrug.nl/genome2d_results/GSEA_pro/193.136.177.1i6fcn62stkevgvfg07il3k7hj187/GSEA_Pro.single_list.IPR016091.txt) |
| IPR | [IPR016181](http://www.ebi.ac.uk/interpro/entry/IPR016181) | Acyl-CoA N-acyltransferase | 34 | [2.1e-02 ( 4)](http://genome2d.molgenrug.nl/genome2d_results/GSEA_pro/193.136.177.1i6fcn62stkevgvfg07il3k7hj187/GSEA_Pro.single_list.IPR016181.txt) |
| IPR | [IPR019931](http://www.ebi.ac.uk/interpro/entry/IPR019931) | LPXTG cell wall anchor domain | 16 | [7.7e-04 ( 5)](http://genome2d.molgenrug.nl/genome2d_results/GSEA_pro/193.136.177.1i6fcn62stkevgvfg07il3k7hj187/GSEA_Pro.single_list.IPR019931.txt) |
| IPR | [IPR019948](http://www.ebi.ac.uk/interpro/entry/IPR019948) | Gram-positive anchor | 13 | [1.3e-02 ( 3)](http://genome2d.molgenrug.nl/genome2d_results/GSEA_pro/193.136.177.1i6fcn62stkevgvfg07il3k7hj187/GSEA_Pro.single_list.IPR019948.txt) |
| IPR | [IPR020845](http://www.ebi.ac.uk/interpro/entry/IPR020845) | AMP-binding, conserved site | 5 | [1.8e-02 ( 2)](http://genome2d.molgenrug.nl/genome2d_results/GSEA_pro/193.136.177.1i6fcn62stkevgvfg07il3k7hj187/GSEA_Pro.single_list.IPR020845.txt) |
| IPR | [IPR025110](http://www.ebi.ac.uk/interpro/entry/IPR025110) | AMP-binding enzyme C-terminal domain | 7 | [2.1e-02 ( 2)](http://genome2d.molgenrug.nl/genome2d_results/GSEA_pro/193.136.177.1i6fcn62stkevgvfg07il3k7hj187/GSEA_Pro.single_list.IPR025110.txt) |
| IPR | [IPR029058](http://www.ebi.ac.uk/interpro/entry/IPR029058) | Alpha/Beta hydrolase fold | 27 | [4.3e-02 ( 3)](http://genome2d.molgenrug.nl/genome2d_results/GSEA_pro/193.136.177.1i6fcn62stkevgvfg07il3k7hj187/GSEA_Pro.single_list.IPR029058.txt) |
| IPR | [IPR029061](http://www.ebi.ac.uk/interpro/entry/IPR029061) | Thiamin diphosphate-binding fold | 13 | [1.3e-02 ( 3)](http://genome2d.molgenrug.nl/genome2d_results/GSEA_pro/193.136.177.1i6fcn62stkevgvfg07il3k7hj187/GSEA_Pro.single_list.IPR029061.txt) |
| Pfam | [PF04507](http://pfam.xfam.org/family/PF04507) | Protein of unknown function, DUF576 | 16 | [3.7e-11 (8)](http://genome2d.molgenrug.nl/genome2d_results/GSEA_pro/193.136.177.1i6fcn62stkevgvfg07il3k7hj187/GSEA_Pro.single_list.PF04507.txt) |
| Pfam | [PF07564](http://pfam.xfam.org/family/PF07564) | SAUSA300_RS07235 | 2 | [4.4e-04 (2)](http://genome2d.molgenrug.nl/genome2d_results/GSEA_pro/193.136.177.1i6fcn62stkevgvfg07il3k7hj187/GSEA_Pro.single_list.PF07564.txt) |
| Pfam | [PF00210](http://pfam.xfam.org/family/PF00210) | Ferritin-like domain | 2 | [4.4e-04 (2)](http://genome2d.molgenrug.nl/genome2d_results/GSEA_pro/193.136.177.1i6fcn62stkevgvfg07il3k7hj187/GSEA_Pro.single_list.PF00210.txt) |
| Pfam | [PF04650](http://pfam.xfam.org/family/PF04650) | SAUSA300_RS00585 | 15 | [8.9e-08 (6)](http://genome2d.molgenrug.nl/genome2d_results/GSEA_pro/193.136.177.1i6fcn62stkevgvfg07il3k7hj187/GSEA_Pro.single_list.PF04650.txt) |
| Pfam | [PF00126](http://pfam.xfam.org/family/PF00126) | Bacterial regulatory helix-turn-helix protein, lysR family | 7 | [2.2e-04 (3)](http://genome2d.molgenrug.nl/genome2d_results/GSEA_pro/193.136.177.1i6fcn62stkevgvfg07il3k7hj187/GSEA_Pro.single_list.PF00126.txt) |
| Pfam | [PF01476](http://pfam.xfam.org/family/PF01476) | LysM domain | 6 | [1.5e-04 (3)](http://genome2d.molgenrug.nl/genome2d_results/GSEA_pro/193.136.177.1i6fcn62stkevgvfg07il3k7hj187/GSEA_Pro.single_list.PF01476.txt) |
| Pfam | [PF03466](http://pfam.xfam.org/family/PF03466) | LysR substrate binding domain | 6 | [1.5e-04 (3)](http://genome2d.molgenrug.nl/genome2d_results/GSEA_pro/193.136.177.1i6fcn62stkevgvfg07il3k7hj187/GSEA_Pro.single_list.PF03466.txt) |
| Pfam | [PF05257](http://pfam.xfam.org/family/PF05257) | CHAP domain | 13 | [8.9e-05 (4)](http://genome2d.molgenrug.nl/genome2d_results/GSEA_pro/193.136.177.1i6fcn62stkevgvfg07il3k7hj187/GSEA_Pro.single_list.PF05257.txt) |
| Pfam | [PF05738](http://pfam.xfam.org/family/PF05738) | Cna protein B-type domain | 3 | [1.1e-03 (2)](http://genome2d.molgenrug.nl/genome2d_results/GSEA_pro/193.136.177.1i6fcn62stkevgvfg07il3k7hj187/GSEA_Pro.single_list.PF05738.txt) |
| Pfam | [PF00271](http://pfam.xfam.org/family/PF00271) | Helicase conserved C-terminal domain | 10 | [6.3e-03 (2)](http://genome2d.molgenrug.nl/genome2d_results/GSEA_pro/193.136.177.1i6fcn62stkevgvfg07il3k7hj187/GSEA_Pro.single_list.PF00271.txt) |
| Pfam | [PF00291](http://pfam.xfam.org/family/PF00291) | Pyridoxal-phosphate dependent enzyme | 7 | [4.0e-03 (2)](http://genome2d.molgenrug.nl/genome2d_results/GSEA_pro/193.136.177.1i6fcn62stkevgvfg07il3k7hj187/GSEA_Pro.single_list.PF00291.txt) |
| Pfam | [PF00440](http://pfam.xfam.org/family/PF00440) | Bacterial regulatory proteins, tetR family | 7 | [4.0e-03 (2)](http://genome2d.molgenrug.nl/genome2d_results/GSEA_pro/193.136.177.1i6fcn62stkevgvfg07il3k7hj187/GSEA_Pro.single_list.PF00440.txt) |
| Pfam | [PF00486](http://pfam.xfam.org/family/PF00486) | Transcriptional regulatory protein, C terminal | 9 | [5.5e-03 (2)](http://genome2d.molgenrug.nl/genome2d_results/GSEA_pro/193.136.177.1i6fcn62stkevgvfg07il3k7hj187/GSEA_Pro.single_list.PF00486.txt) |
| Pfam | [PF00501](http://pfam.xfam.org/family/PF00501) | AMP-binding enzyme | 7 | [4.0e-03 (2)](http://genome2d.molgenrug.nl/genome2d_results/GSEA_pro/193.136.177.1i6fcn62stkevgvfg07il3k7hj187/GSEA_Pro.single_list.PF00501.txt) |
| Pfam | [PF00746](http://pfam.xfam.org/family/PF00746) | SAUSA300_RS00585 | 13 | [1.1e-03 (3)](http://genome2d.molgenrug.nl/genome2d_results/GSEA_pro/193.136.177.1i6fcn62stkevgvfg07il3k7hj187/GSEA_Pro.single_list.PF00746.txt) |
| Pfam | [PF00753](http://pfam.xfam.org/family/PF00753) | Metallo-beta-lactamase superfamily | 8 | [4.8e-03 (2)](http://genome2d.molgenrug.nl/genome2d_results/GSEA_pro/193.136.177.1i6fcn62stkevgvfg07il3k7hj187/GSEA_Pro.single_list.PF00753.txt) |
| Pfam | [PF02775](http://pfam.xfam.org/family/PF02775) | Thiamine pyrophosphate enzyme, C-terminal TPP binding domain | 6 | [4.0e-03 (2)](http://genome2d.molgenrug.nl/genome2d_results/GSEA_pro/193.136.177.1i6fcn62stkevgvfg07il3k7hj187/GSEA_Pro.single_list.PF02775.txt) |
| Pfam | [PF02876](http://pfam.xfam.org/family/PF02876) | Staphylococcal/Streptococcal toxin, beta-grasp domain | 18 | [2.5e-03 (3)](http://genome2d.molgenrug.nl/genome2d_results/GSEA_pro/193.136.177.1i6fcn62stkevgvfg07il3k7hj187/GSEA_Pro.single_list.PF02876.txt) |
| Pfam | [PF04634](http://pfam.xfam.org/family/PF04634) | Protein of unknown function, DUF600 | 9 | [5.5e-03 (2)](http://genome2d.molgenrug.nl/genome2d_results/GSEA_pro/193.136.177.1i6fcn62stkevgvfg07il3k7hj187/GSEA_Pro.single_list.PF04634.txt) |
| Pfam | [PF08282](http://pfam.xfam.org/family/PF08282) | haloacid dehalogenase-like hydrolase | 6 | [4.0e-03 (2)](http://genome2d.molgenrug.nl/genome2d_results/GSEA_pro/193.136.177.1i6fcn62stkevgvfg07il3k7hj187/GSEA_Pro.single_list.PF08282.txt) |
| Pfam | [PF10425](http://pfam.xfam.org/family/PF10425) | C-terminus of bacterial fibrinogen-binding adhesin | 7 | [4.0e-03 (2)](http://genome2d.molgenrug.nl/genome2d_results/GSEA_pro/193.136.177.1i6fcn62stkevgvfg07il3k7hj187/GSEA_Pro.single_list.PF10425.txt) |
| Pfam | [PF13193](http://pfam.xfam.org/family/PF13193) | AMP-binding enzyme C-terminal domain | 7 | [4.0e-03 (2)](http://genome2d.molgenrug.nl/genome2d_results/GSEA_pro/193.136.177.1i6fcn62stkevgvfg07il3k7hj187/GSEA_Pro.single_list.PF13193.txt) |
| Pfam | [PF13673](http://pfam.xfam.org/family/PF13673) | Acetyltransferase (GNAT) domain | 8 | [4.8e-03 (2)](http://genome2d.molgenrug.nl/genome2d_results/GSEA_pro/193.136.177.1i6fcn62stkevgvfg07il3k7hj187/GSEA_Pro.single_list.PF13673.txt) |
| SMART | [SM00257](http://smart.embl-heidelberg.de/smart/do_annotation.pl?BLAST=DUMMY&DOMAIN=SM00257) | Lysin motif | 6 | [0.00028 (3)](http://genome2d.molgenrug.nl/genome2d_results/GSEA_pro/193.136.177.1i6fcn62stkevgvfg07il3k7hj187/GSEA_Pro.single_list.SM00257.txt) |
| SMART | [SM00490](http://smart.embl-heidelberg.de/smart/do_annotation.pl?BLAST=DUMMY&DOMAIN=SM00490) | helicase superfamily c-terminal domain | 10 | [0.01405 (2)](http://genome2d.molgenrug.nl/genome2d_results/GSEA_pro/193.136.177.1i6fcn62stkevgvfg07il3k7hj187/GSEA_Pro.single_list.SM00490.txt) |
| SMART | [SM00849](http://smart.embl-heidelberg.de/smart/do_annotation.pl?BLAST=DUMMY&DOMAIN=SM00849) | Metallo-beta-lactamase superfamily | 9 | [0.01405 (2)](http://genome2d.molgenrug.nl/genome2d_results/GSEA_pro/193.136.177.1i6fcn62stkevgvfg07il3k7hj187/GSEA_Pro.single_list.SM00849.txt) |
| SMART | [SM00862](http://smart.embl-heidelberg.de/smart/do_annotation.pl?BLAST=DUMMY&DOMAIN=SM00862) | Transcriptional regulatory protein, C terminal | 9 | [0.01405 (2)](http://genome2d.molgenrug.nl/genome2d_results/GSEA_pro/193.136.177.1i6fcn62stkevgvfg07il3k7hj187/GSEA_Pro.single_list.SM00862.txt) |
| SUPERFAMILY | [SSF117074](http://supfam.cs.bris.ac.uk/SUPERFAMILY/cgi-bin/scop.cgi?ipid=SSF117074) | Hypothetical protein PA1324 | 3 | [0.00348 ( 2)](http://genome2d.molgenrug.nl/genome2d_results/GSEA_pro/193.136.177.1i6fcn62stkevgvfg07il3k7hj187/GSEA_Pro.single_list.SSF117074.txt) |
| SUPERFAMILY | [SSF47240](http://supfam.cs.bris.ac.uk/SUPERFAMILY/cgi-bin/scop.cgi?ipid=SSF47240) | Ferritin-like | 3 | [0.00348 ( 2)](http://genome2d.molgenrug.nl/genome2d_results/GSEA_pro/193.136.177.1i6fcn62stkevgvfg07il3k7hj187/GSEA_Pro.single_list.SSF47240.txt) |
| SUPERFAMILY | [SSF48150](http://supfam.cs.bris.ac.uk/SUPERFAMILY/cgi-bin/scop.cgi?ipid=SSF48150) | DNA-glycosylase | 4 | [0.00492 ( 2)](http://genome2d.molgenrug.nl/genome2d_results/GSEA_pro/193.136.177.1i6fcn62stkevgvfg07il3k7hj187/GSEA_Pro.single_list.SSF48150.txt) |
| SUPERFAMILY | [SSF52218](http://supfam.cs.bris.ac.uk/SUPERFAMILY/cgi-bin/scop.cgi?ipid=SSF52218) | Flavoproteins | 7 | [0.00144 ( 3)](http://genome2d.molgenrug.nl/genome2d_results/GSEA_pro/193.136.177.1i6fcn62stkevgvfg07il3k7hj187/GSEA_Pro.single_list.SSF52218.txt) |
| SUPERFAMILY | [SSF54106](http://supfam.cs.bris.ac.uk/SUPERFAMILY/cgi-bin/scop.cgi?ipid=SSF54106) | LysM domain | 6 | [0.00134 ( 3)](http://genome2d.molgenrug.nl/genome2d_results/GSEA_pro/193.136.177.1i6fcn62stkevgvfg07il3k7hj187/GSEA_Pro.single_list.SSF54106.txt) |
| SUPERFAMILY | [SSF54862](http://supfam.cs.bris.ac.uk/SUPERFAMILY/cgi-bin/scop.cgi?ipid=SSF54862) | 4Fe-4S ferredoxins | 4 | [0.00492 ( 2)](http://genome2d.molgenrug.nl/genome2d_results/GSEA_pro/193.136.177.1i6fcn62stkevgvfg07il3k7hj187/GSEA_Pro.single_list.SSF54862.txt) |
| SUPERFAMILY | [SSF160424](http://supfam.cs.bris.ac.uk/SUPERFAMILY/cgi-bin/scop.cgi?ipid=SSF160424) | BH3703-like | 9 | [0.01854 ( 2)](http://genome2d.molgenrug.nl/genome2d_results/GSEA_pro/193.136.177.1i6fcn62stkevgvfg07il3k7hj187/GSEA_Pro.single_list.SSF160424.txt) |
| SUPERFAMILY | [SSF46785](http://supfam.cs.bris.ac.uk/SUPERFAMILY/cgi-bin/scop.cgi?ipid=SSF46785) | "Winged helix" DNA-binding domain | 67 | [0.00059 ( 8)](http://genome2d.molgenrug.nl/genome2d_results/GSEA_pro/193.136.177.1i6fcn62stkevgvfg07il3k7hj187/GSEA_Pro.single_list.SSF46785.txt) |
| SUPERFAMILY | [SSF49401](http://supfam.cs.bris.ac.uk/SUPERFAMILY/cgi-bin/scop.cgi?ipid=SSF49401) | Bacterial adhesins | 7 | [0.01163 ( 2)](http://genome2d.molgenrug.nl/genome2d_results/GSEA_pro/193.136.177.1i6fcn62stkevgvfg07il3k7hj187/GSEA_Pro.single_list.SSF49401.txt) |
| SUPERFAMILY | [SSF50203](http://supfam.cs.bris.ac.uk/SUPERFAMILY/cgi-bin/scop.cgi?ipid=SSF50203) | Bacterial enterotoxins | 14 | [0.00492 ( 3)](http://genome2d.molgenrug.nl/genome2d_results/GSEA_pro/193.136.177.1i6fcn62stkevgvfg07il3k7hj187/GSEA_Pro.single_list.SSF50203.txt) |
| SUPERFAMILY | [SSF51735](http://supfam.cs.bris.ac.uk/SUPERFAMILY/cgi-bin/scop.cgi?ipid=SSF51735) | NAD(P)-binding Rossmann-fold domains | 72 | [0.00059 ( 8)](http://genome2d.molgenrug.nl/genome2d_results/GSEA_pro/193.136.177.1i6fcn62stkevgvfg07il3k7hj187/GSEA_Pro.single_list.SSF51735.txt) |
| SUPERFAMILY | [SSF52518](http://supfam.cs.bris.ac.uk/SUPERFAMILY/cgi-bin/scop.cgi?ipid=SSF52518) | Thiamin diphosphate-binding fold (THDP-binding) | 13 | [0.00477 ( 3)](http://genome2d.molgenrug.nl/genome2d_results/GSEA_pro/193.136.177.1i6fcn62stkevgvfg07il3k7hj187/GSEA_Pro.single_list.SSF52518.txt) |
| SUPERFAMILY | [SSF52833](http://supfam.cs.bris.ac.uk/SUPERFAMILY/cgi-bin/scop.cgi?ipid=SSF52833) | Thioredoxin-like | 19 | [0.01028 ( 3)](http://genome2d.molgenrug.nl/genome2d_results/GSEA_pro/193.136.177.1i6fcn62stkevgvfg07il3k7hj187/GSEA_Pro.single_list.SSF52833.txt) |
| SUPERFAMILY | [SSF53474](http://supfam.cs.bris.ac.uk/SUPERFAMILY/cgi-bin/scop.cgi?ipid=SSF53474) | alpha/beta-Hydrolases | 27 | [0.02037 ( 3)](http://genome2d.molgenrug.nl/genome2d_results/GSEA_pro/193.136.177.1i6fcn62stkevgvfg07il3k7hj187/GSEA_Pro.single_list.SSF53474.txt) |
| SUPERFAMILY | [SSF53686](http://supfam.cs.bris.ac.uk/SUPERFAMILY/cgi-bin/scop.cgi?ipid=SSF53686) | Tryptophan synthase beta subunit-like PLP-dependent enzymes | 7 | [0.01163 ( 2)](http://genome2d.molgenrug.nl/genome2d_results/GSEA_pro/193.136.177.1i6fcn62stkevgvfg07il3k7hj187/GSEA_Pro.single_list.SSF53686.txt) |
| SUPERFAMILY | [SSF53850](http://supfam.cs.bris.ac.uk/SUPERFAMILY/cgi-bin/scop.cgi?ipid=SSF53850) | Periplasmic binding protein-like II | 28 | [0.02154 ( 3)](http://genome2d.molgenrug.nl/genome2d_results/GSEA_pro/193.136.177.1i6fcn62stkevgvfg07il3k7hj187/GSEA_Pro.single_list.SSF53850.txt) |
| SUPERFAMILY | [SSF53901](http://supfam.cs.bris.ac.uk/SUPERFAMILY/cgi-bin/scop.cgi?ipid=SSF53901) | Thiolase-like | 7 | [0.01163 ( 2)](http://genome2d.molgenrug.nl/genome2d_results/GSEA_pro/193.136.177.1i6fcn62stkevgvfg07il3k7hj187/GSEA_Pro.single_list.SSF53901.txt) |
| SUPERFAMILY | [SSF54001](http://supfam.cs.bris.ac.uk/SUPERFAMILY/cgi-bin/scop.cgi?ipid=SSF54001) | Cysteine proteinases | 17 | [0.00144 ( 4)](http://genome2d.molgenrug.nl/genome2d_results/GSEA_pro/193.136.177.1i6fcn62stkevgvfg07il3k7hj187/GSEA_Pro.single_list.SSF54001.txt) |
| SUPERFAMILY | [SSF54334](http://supfam.cs.bris.ac.uk/SUPERFAMILY/cgi-bin/scop.cgi?ipid=SSF54334) | Superantigen toxins, C-terminal domain | 20 | [0.00232 ( 4)](http://genome2d.molgenrug.nl/genome2d_results/GSEA_pro/193.136.177.1i6fcn62stkevgvfg07il3k7hj187/GSEA_Pro.single_list.SSF54334.txt) |
| SUPERFAMILY | [SSF55347](http://supfam.cs.bris.ac.uk/SUPERFAMILY/cgi-bin/scop.cgi?ipid=SSF55347) | Glyceraldehyde-3-phosphate dehydrogenase-like, C-terminal domain | 9 | [0.00232 ( 3)](http://genome2d.molgenrug.nl/genome2d_results/GSEA_pro/193.136.177.1i6fcn62stkevgvfg07il3k7hj187/GSEA_Pro.single_list.SSF55347.txt) |
| SUPERFAMILY | [SSF55729](http://supfam.cs.bris.ac.uk/SUPERFAMILY/cgi-bin/scop.cgi?ipid=SSF55729) | Acyl-CoA N-acyltransferases (Nat) | 34 | [0.00744 ( 4)](http://genome2d.molgenrug.nl/genome2d_results/GSEA_pro/193.136.177.1i6fcn62stkevgvfg07il3k7hj187/GSEA_Pro.single_list.SSF55729.txt) |
| SUPERFAMILY | [SSF56801](http://supfam.cs.bris.ac.uk/SUPERFAMILY/cgi-bin/scop.cgi?ipid=SSF56801) | Acetyl-CoA synthetase-like | 7 | [0.01163 ( 2)](http://genome2d.molgenrug.nl/genome2d_results/GSEA_pro/193.136.177.1i6fcn62stkevgvfg07il3k7hj187/GSEA_Pro.single_list.SSF56801.txt) |
| **TopHits (downregulated genes)** |  |  |  |  |
| KEGG | [520](http://www.genome.jp/kegg-bin/show_pathway?saa00520) | Amino sugar and nucleotide sugar metabolism | 38 | [3.9e-05 (9)](http://www.genome.jp/kegg-bin/show_pathway?saa00520+SAUSA300_RS01015+SAUSA300_RS01680+SAUSA300_RS01695+SAUSA300_RS01770+SAUSA300_RS03060+SAUSA300_RS03070+SAUSA300_RS03675+SAUSA300_RS08230+SAUSA300_RS13740) |
| KEGG | [900](http://www.genome.jp/kegg-bin/show_pathway?saa00900) | Terpenoid backbone biosynthesis | 25 | [3.2e-05 (8)](http://www.genome.jp/kegg-bin/show_pathway?saa00900+SAUSA300_RS01770+SAUSA300_RS03060+SAUSA300_RS03065+SAUSA300_RS03070+SAUSA300_RS03675+SAUSA300_RS13740+SAUSA300_RS13790+SAUSA300_RS13795) |
| KEGG | [970](http://www.genome.jp/kegg-bin/show_pathway?saa00970) | Aminoacyl-tRNA biosynthesis | 29 | [3.9e-05 (8)](http://www.genome.jp/kegg-bin/show_pathway?saa00970+SAUSA300_RS01930+SAUSA300_RS05585+SAUSA300_RS05590+SAUSA300_RS07335+SAUSA300_RS08320+SAUSA300_RS08650+SAUSA300_RS08780+SAUSA300_RS09145) |
| KEGG | [52](http://www.genome.jp/kegg-bin/show_pathway?saa00052) | Galactose metabolism | 19 | [2.4e-02 (4)](http://www.genome.jp/kegg-bin/show_pathway?saa00052+SAUSA300_RS03060+SAUSA300_RS03070+SAUSA300_RS03670+SAUSA300_RS08230) |
| KEGG | [61](http://www.genome.jp/kegg-bin/show_pathway?saa00061) | Fatty acid biosynthesis | 7 | [1.0e-02 (3)](http://www.genome.jp/kegg-bin/show_pathway?saa00061+SAUSA300_RS04770+SAUSA300_RS06075+SAUSA300_RS06080) |
| KEGG | [195](http://www.genome.jp/kegg-bin/show_pathway?saa00195) | Photosynthesis | 5 | [4.0e-02 (2)](http://www.genome.jp/kegg-bin/show_pathway?saa00195+SAUSA300_RS11335+SAUSA300_RS11345) |
| KEGG | [240](http://www.genome.jp/kegg-bin/show_pathway?saa00240) | Pyrimidine metabolism | 46 | [2.8e-02 (6)](http://www.genome.jp/kegg-bin/show_pathway?saa00240+SAUSA300_RS01665+SAUSA300_RS05915+SAUSA300_RS05925+SAUSA300_RS11455+SAUSA300_RS11520+SAUSA300_RS14015) |
| KEGG | [640](http://www.genome.jp/kegg-bin/show_pathway?saa00640) | Propanoate metabolism | 13 | [4.0e-02 (3)](http://www.genome.jp/kegg-bin/show_pathway?saa00640+SAUSA300_RS01155+SAUSA300_RS01250+SAUSA300_RS14075) |
| COG | [P](http://ecoliwiki.net/colipedia/index.php/Clusters_of_Orthologous_Groups_(COGs)P) | Inorganic ion transport and metabolism | 230 | [0.0e+00 (44)](http://genome2d.molgenrug.nl/genome2d_results/GSEA_pro/193.136.177.1i6fcn62stkevgvfg07il3k7hj1826/GSEA_Pro.single_list.P.txt) |
| COG | [J](http://ecoliwiki.net/colipedia/index.php/Clusters_of_Orthologous_Groups_(COGs)J) | Translation, ribosomal structure and biogenesis | 171 | [0.0e+00 (34)](http://genome2d.molgenrug.nl/genome2d_results/GSEA_pro/193.136.177.1i6fcn62stkevgvfg07il3k7hj1826/GSEA_Pro.single_list.J.txt) |
| COG | [E](http://ecoliwiki.net/colipedia/index.php/Clusters_of_Orthologous_Groups_(COGs)E) | Amino acid transport and metabolism | 86 | [4.2e-10 (11)](http://genome2d.molgenrug.nl/genome2d_results/GSEA_pro/193.136.177.1i6fcn62stkevgvfg07il3k7hj1826/GSEA_Pro.single_list.E.txt) |
| COG | [F](http://ecoliwiki.net/colipedia/index.php/Clusters_of_Orthologous_Groups_(COGs)F) | Nucleotide transport and metabolism | 47 | [3.2e-08 ( 8)](http://genome2d.molgenrug.nl/genome2d_results/GSEA_pro/193.136.177.1i6fcn62stkevgvfg07il3k7hj1826/GSEA_Pro.single_list.F.txt) |
| COG | [O](http://ecoliwiki.net/colipedia/index.php/Clusters_of_Orthologous_Groups_(COGs)O) | Posttranslational modification, protein turnover, chaperones | 151 | [0.0e+00 (20)](http://genome2d.molgenrug.nl/genome2d_results/GSEA_pro/193.136.177.1i6fcn62stkevgvfg07il3k7hj1826/GSEA_Pro.single_list.O.txt) |
| COG | [M](http://ecoliwiki.net/colipedia/index.php/Clusters_of_Orthologous_Groups_(COGs)M) | Cell wall/membrane/envelope biogenesis | 97 | [5.6e-11 (12)](http://genome2d.molgenrug.nl/genome2d_results/GSEA_pro/193.136.177.1i6fcn62stkevgvfg07il3k7hj1826/GSEA_Pro.single_list.M.txt) |
| COG | [T](http://ecoliwiki.net/colipedia/index.php/Clusters_of_Orthologous_Groups_(COGs)T) | Signal transduction mechanisms | 87 | [1.7e-11 (12)](http://genome2d.molgenrug.nl/genome2d_results/GSEA_pro/193.136.177.1i6fcn62stkevgvfg07il3k7hj1826/GSEA_Pro.single_list.T.txt) |
| COG | [R](http://ecoliwiki.net/colipedia/index.php/Clusters_of_Orthologous_Groups_(COGs)R) | General function prediction only | 412 | [0.0e+00 (54)](http://genome2d.molgenrug.nl/genome2d_results/GSEA_pro/193.136.177.1i6fcn62stkevgvfg07il3k7hj1826/GSEA_Pro.single_list.R.txt) |
| COG | [Q](http://ecoliwiki.net/colipedia/index.php/Clusters_of_Orthologous_Groups_(COGs)Q) | Secondary metabolites biosynthesis, transport and catabolism | 21 | [1.9e-03 ( 3)](http://genome2d.molgenrug.nl/genome2d_results/GSEA_pro/193.136.177.1i6fcn62stkevgvfg07il3k7hj1826/GSEA_Pro.single_list.Q.txt) |
| COG | [C](http://ecoliwiki.net/colipedia/index.php/Clusters_of_Orthologous_Groups_(COGs)C) | Energy production and conversion | 36 | [6.2e-05 ( 5)](http://genome2d.molgenrug.nl/genome2d_results/GSEA_pro/193.136.177.1i6fcn62stkevgvfg07il3k7hj1826/GSEA_Pro.single_list.C.txt) |
| COG | [G](http://ecoliwiki.net/colipedia/index.php/Clusters_of_Orthologous_Groups_(COGs)G) | Carbohydrate transport and metabolism | 27 | [2.8e-04 ( 4)](http://genome2d.molgenrug.nl/genome2d_results/GSEA_pro/193.136.177.1i6fcn62stkevgvfg07il3k7hj1826/GSEA_Pro.single_list.G.txt) |
| COG | [H](http://ecoliwiki.net/colipedia/index.php/Clusters_of_Orthologous_Groups_(COGs)H) | Coenzyme transport and metabolism | 34 | [6.4e-04 ( 4)](http://genome2d.molgenrug.nl/genome2d_results/GSEA_pro/193.136.177.1i6fcn62stkevgvfg07il3k7hj1826/GSEA_Pro.single_list.H.txt) |
| COG | [I](http://ecoliwiki.net/colipedia/index.php/Clusters_of_Orthologous_Groups_(COGs)I) | Lipid transport and metabolism | 31 | [3.2e-05 ( 5)](http://genome2d.molgenrug.nl/genome2d_results/GSEA_pro/193.136.177.1i6fcn62stkevgvfg07il3k7hj1826/GSEA_Pro.single_list.I.txt) |
| COG | [S](http://ecoliwiki.net/colipedia/index.php/Clusters_of_Orthologous_Groups_(COGs)S) | Function unknown | 31 | [3.2e-05 ( 5)](http://genome2d.molgenrug.nl/genome2d_results/GSEA_pro/193.136.177.1i6fcn62stkevgvfg07il3k7hj1826/GSEA_Pro.single_list.S.txt) |
| GO | [GO:0015986](http://www.ebi.ac.uk/QuickGO/GTerm?id=GO:0015986) | ATP synthesis coupled proton transport | 8 | [6.5e-10 ( 8)](http://genome2d.molgenrug.nl/genome2d_results/GSEA_pro/193.136.177.1i6fcn62stkevgvfg07il3k7hj1826/GSEA_Pro.single_list.GO:0015986.txt) |
| GO | [GO:0003735](http://www.ebi.ac.uk/QuickGO/GTerm?id=GO:0003735) | structural constituent of ribosome | 55 | [5.9e-15 (23)](http://genome2d.molgenrug.nl/genome2d_results/GSEA_pro/193.136.177.1i6fcn62stkevgvfg07il3k7hj1826/GSEA_Pro.single_list.GO:0003735.txt) |
| GO | [GO:0046933](http://www.ebi.ac.uk/QuickGO/GTerm?id=GO:0046933) | proton-transporting ATP synthase activity, rotational mechanism | 5 | [3.6e-06 ( 5)](http://genome2d.molgenrug.nl/genome2d_results/GSEA_pro/193.136.177.1i6fcn62stkevgvfg07il3k7hj1826/GSEA_Pro.single_list.GO:0046933.txt) |
| GO | [GO:0005840](http://www.ebi.ac.uk/QuickGO/GTerm?id=GO:0005840) | ribosome | 52 | [9.8e-14 (21)](http://genome2d.molgenrug.nl/genome2d_results/GSEA_pro/193.136.177.1i6fcn62stkevgvfg07il3k7hj1826/GSEA_Pro.single_list.GO:0005840.txt) |
| GO | [GO:0006412](http://www.ebi.ac.uk/QuickGO/GTerm?id=GO:0006412) | translation | 57 | [7.8e-15 (23)](http://genome2d.molgenrug.nl/genome2d_results/GSEA_pro/193.136.177.1i6fcn62stkevgvfg07il3k7hj1826/GSEA_Pro.single_list.GO:0006412.txt) |
| GO | [GO:0045261](http://www.ebi.ac.uk/QuickGO/GTerm?id=GO:0045261) | proton-transporting ATP synthase complex, catalytic core F(1) | 3 | [5.1e-04 ( 3)](http://genome2d.molgenrug.nl/genome2d_results/GSEA_pro/193.136.177.1i6fcn62stkevgvfg07il3k7hj1826/GSEA_Pro.single_list.GO:0045261.txt) |
| GO | [GO:0046961](http://www.ebi.ac.uk/QuickGO/GTerm?id=GO:0046961) | proton-transporting ATPase activity, rotational mechanism | 3 | [5.1e-04 ( 3)](http://genome2d.molgenrug.nl/genome2d_results/GSEA_pro/193.136.177.1i6fcn62stkevgvfg07il3k7hj1826/GSEA_Pro.single_list.GO:0046961.txt) |
| GO | [GO:0015991](http://www.ebi.ac.uk/QuickGO/GTerm?id=GO:0015991) | ATP hydrolysis coupled proton transport | 3 | [5.1e-04 ( 3)](http://genome2d.molgenrug.nl/genome2d_results/GSEA_pro/193.136.177.1i6fcn62stkevgvfg07il3k7hj1826/GSEA_Pro.single_list.GO:0015991.txt) |
| GO | [GO:0015078](http://www.ebi.ac.uk/QuickGO/GTerm?id=GO:0015078) | hydrogen ion transmembrane transporter activity | 3 | [5.1e-04 ( 3)](http://genome2d.molgenrug.nl/genome2d_results/GSEA_pro/193.136.177.1i6fcn62stkevgvfg07il3k7hj1826/GSEA_Pro.single_list.GO:0015078.txt) |
| GO | [GO:0016820](http://www.ebi.ac.uk/QuickGO/GTerm?id=GO:0016820) | hydrolase activity, acting on acid anhydrides, catalyzing transmembrane movement of substances | 4 | [1.8e-03 ( 3)](http://genome2d.molgenrug.nl/genome2d_results/GSEA_pro/193.136.177.1i6fcn62stkevgvfg07il3k7hj1826/GSEA_Pro.single_list.GO:0016820.txt) |
| GO | [GO:0019843](http://www.ebi.ac.uk/QuickGO/GTerm?id=GO:0019843) | rRNA binding | 5 | [2.2e-04 ( 4)](http://genome2d.molgenrug.nl/genome2d_results/GSEA_pro/193.136.177.1i6fcn62stkevgvfg07il3k7hj1826/GSEA_Pro.single_list.GO:0019843.txt) |
| GO | [GO:0022904](http://www.ebi.ac.uk/QuickGO/GTerm?id=GO:0022904) | respiratory electron transport chain | 2 | [5.2e-03 ( 2)](http://genome2d.molgenrug.nl/genome2d_results/GSEA_pro/193.136.177.1i6fcn62stkevgvfg07il3k7hj1826/GSEA_Pro.single_list.GO:0022904.txt) |
| GO | [GO:0033178](http://www.ebi.ac.uk/QuickGO/GTerm?id=GO:0033178) | proton-transporting two-sector ATPase complex, catalytic domain | 2 | [5.2e-03 ( 2)](http://genome2d.molgenrug.nl/genome2d_results/GSEA_pro/193.136.177.1i6fcn62stkevgvfg07il3k7hj1826/GSEA_Pro.single_list.GO:0033178.txt) |
| GO | [GO:0045263](http://www.ebi.ac.uk/QuickGO/GTerm?id=GO:0045263) | proton-transporting ATP synthase complex, coupling factor F(o) | 2 | [5.2e-03 ( 2)](http://genome2d.molgenrug.nl/genome2d_results/GSEA_pro/193.136.177.1i6fcn62stkevgvfg07il3k7hj1826/GSEA_Pro.single_list.GO:0045263.txt) |
| GO | [GO:0046034](http://www.ebi.ac.uk/QuickGO/GTerm?id=GO:0046034) | ATP metabolic process | 2 | [5.2e-03 ( 2)](http://genome2d.molgenrug.nl/genome2d_results/GSEA_pro/193.136.177.1i6fcn62stkevgvfg07il3k7hj1826/GSEA_Pro.single_list.GO:0046034.txt) |
| GO | [GO:0016020](http://www.ebi.ac.uk/QuickGO/GTerm?id=GO:0016020) | membrane | 180 | [5.9e-14 (37)](http://genome2d.molgenrug.nl/genome2d_results/GSEA_pro/193.136.177.1i6fcn62stkevgvfg07il3k7hj1826/GSEA_Pro.single_list.GO:0016020.txt) |
| GO | [GO:0015813](http://www.ebi.ac.uk/QuickGO/GTerm?id=GO:0015813) | L-glutamate transport | 2 | [5.2e-03 ( 2)](http://genome2d.molgenrug.nl/genome2d_results/GSEA_pro/193.136.177.1i6fcn62stkevgvfg07il3k7hj1826/GSEA_Pro.single_list.GO:0015813.txt) |
| GO | [GO:0015935](http://www.ebi.ac.uk/QuickGO/GTerm?id=GO:0015935) | small ribosomal subunit | 6 | [5.1e-04 ( 4)](http://genome2d.molgenrug.nl/genome2d_results/GSEA_pro/193.136.177.1i6fcn62stkevgvfg07il3k7hj1826/GSEA_Pro.single_list.GO:0015935.txt) |
| GO | [GO:0006432](http://www.ebi.ac.uk/QuickGO/GTerm?id=GO:0006432) | phenylalanyl-tRNA aminoacylation | 2 | [5.2e-03 ( 2)](http://genome2d.molgenrug.nl/genome2d_results/GSEA_pro/193.136.177.1i6fcn62stkevgvfg07il3k7hj1826/GSEA_Pro.single_list.GO:0006432.txt) |
| GO | [GO:0001848](http://www.ebi.ac.uk/QuickGO/GTerm?id=GO:0001848) | complement binding | 2 | [5.2e-03 ( 2)](http://genome2d.molgenrug.nl/genome2d_results/GSEA_pro/193.136.177.1i6fcn62stkevgvfg07il3k7hj1826/GSEA_Pro.single_list.GO:0001848.txt) |
| GO | [GO:0004459](http://www.ebi.ac.uk/QuickGO/GTerm?id=GO:0004459) | L-lactate dehydrogenase activity | 2 | [5.2e-03 ( 2)](http://genome2d.molgenrug.nl/genome2d_results/GSEA_pro/193.136.177.1i6fcn62stkevgvfg07il3k7hj1826/GSEA_Pro.single_list.GO:0004459.txt) |
| GO | [GO:0004826](http://www.ebi.ac.uk/QuickGO/GTerm?id=GO:0004826) | phenylalanine-tRNA ligase activity | 2 | [5.2e-03 ( 2)](http://genome2d.molgenrug.nl/genome2d_results/GSEA_pro/193.136.177.1i6fcn62stkevgvfg07il3k7hj1826/GSEA_Pro.single_list.GO:0004826.txt) |
| GO | [GO:0005622](http://www.ebi.ac.uk/QuickGO/GTerm?id=GO:0005622) | intracellular | 54 | [4.1e-08 (16)](http://genome2d.molgenrug.nl/genome2d_results/GSEA_pro/193.136.177.1i6fcn62stkevgvfg07il3k7hj1826/GSEA_Pro.single_list.GO:0005622.txt) |
| GO | [GO:0005615](http://www.ebi.ac.uk/QuickGO/GTerm?id=GO:0005615) | extracellular space | 2 | [5.2e-03 ( 2)](http://genome2d.molgenrug.nl/genome2d_results/GSEA_pro/193.136.177.1i6fcn62stkevgvfg07il3k7hj1826/GSEA_Pro.single_list.GO:0005615.txt) |
| GO | [GO:0004129](http://www.ebi.ac.uk/QuickGO/GTerm?id=GO:0004129) | cytochrome-c oxidase activity | 3 | [1.3e-02 ( 2)](http://genome2d.molgenrug.nl/genome2d_results/GSEA_pro/193.136.177.1i6fcn62stkevgvfg07il3k7hj1826/GSEA_Pro.single_list.GO:0004129.txt) |
| GO | [GO:0004812](http://www.ebi.ac.uk/QuickGO/GTerm?id=GO:0004812) | aminoacyl-tRNA ligase activity | 17 | [5.1e-04 ( 6)](http://genome2d.molgenrug.nl/genome2d_results/GSEA_pro/193.136.177.1i6fcn62stkevgvfg07il3k7hj1826/GSEA_Pro.single_list.GO:0004812.txt) |
| GO | [GO:0005337](http://www.ebi.ac.uk/QuickGO/GTerm?id=GO:0005337) | nucleoside transmembrane transporter activity | 3 | [1.3e-02 ( 2)](http://genome2d.molgenrug.nl/genome2d_results/GSEA_pro/193.136.177.1i6fcn62stkevgvfg07il3k7hj1826/GSEA_Pro.single_list.GO:0005337.txt) |
| GO | [GO:0006810](http://www.ebi.ac.uk/QuickGO/GTerm?id=GO:0006810) | transport | 90 | [2.9e-06 (18)](http://genome2d.molgenrug.nl/genome2d_results/GSEA_pro/193.136.177.1i6fcn62stkevgvfg07il3k7hj1826/GSEA_Pro.single_list.GO:0006810.txt) |
| GO | [GO:0008982](http://www.ebi.ac.uk/QuickGO/GTerm?id=GO:0008982) | protein-N(PI)-phosphohistidine-sugar phosphotransferase activity | 22 | [4.4e-04 ( 7)](http://genome2d.molgenrug.nl/genome2d_results/GSEA_pro/193.136.177.1i6fcn62stkevgvfg07il3k7hj1826/GSEA_Pro.single_list.GO:0008982.txt) |
| GO | [GO:0009401](http://www.ebi.ac.uk/QuickGO/GTerm?id=GO:0009401) | phosphoenolpyruvate-dependent sugar phosphotransferase system | 28 | [5.2e-05 ( 9)](http://genome2d.molgenrug.nl/genome2d_results/GSEA_pro/193.136.177.1i6fcn62stkevgvfg07il3k7hj1826/GSEA_Pro.single_list.GO:0009401.txt) |
| GO | [GO:0016747](http://www.ebi.ac.uk/QuickGO/GTerm?id=GO:0016747) | transferase activity, transferring acyl groups other than amino-acyl groups | 9 | [2.2e-03 ( 4)](http://genome2d.molgenrug.nl/genome2d_results/GSEA_pro/193.136.177.1i6fcn62stkevgvfg07il3k7hj1826/GSEA_Pro.single_list.GO:0016747.txt) |
| GO | [GO:0016773](http://www.ebi.ac.uk/QuickGO/GTerm?id=GO:0016773) | phosphotransferase activity, alcohol group as acceptor | 11 | [5.1e-04 ( 5)](http://genome2d.molgenrug.nl/genome2d_results/GSEA_pro/193.136.177.1i6fcn62stkevgvfg07il3k7hj1826/GSEA_Pro.single_list.GO:0016773.txt) |
| GO | [GO:0019752](http://www.ebi.ac.uk/QuickGO/GTerm?id=GO:0019752) | carboxylic acid metabolic process | 3 | [1.3e-02 ( 2)](http://genome2d.molgenrug.nl/genome2d_results/GSEA_pro/193.136.177.1i6fcn62stkevgvfg07il3k7hj1826/GSEA_Pro.single_list.GO:0019752.txt) |
| GO | [GO:1901642](http://www.ebi.ac.uk/QuickGO/GTerm?id=GO:1901642) | nucleoside transmembrane transport | 3 | [1.3e-02 ( 2)](http://genome2d.molgenrug.nl/genome2d_results/GSEA_pro/193.136.177.1i6fcn62stkevgvfg07il3k7hj1826/GSEA_Pro.single_list.GO:1901642.txt) |
| GO | [GO:0000166](http://www.ebi.ac.uk/QuickGO/GTerm?id=GO:0000166) | nucleotide binding | 39 | [2.0e-03 ( 8)](http://genome2d.molgenrug.nl/genome2d_results/GSEA_pro/193.136.177.1i6fcn62stkevgvfg07il3k7hj1826/GSEA_Pro.single_list.GO:0000166.txt) |
| GO | [GO:0003333](http://www.ebi.ac.uk/QuickGO/GTerm?id=GO:0003333) | amino acid transmembrane transport | 14 | [8.6e-03 ( 4)](http://genome2d.molgenrug.nl/genome2d_results/GSEA_pro/193.136.177.1i6fcn62stkevgvfg07il3k7hj1826/GSEA_Pro.single_list.GO:0003333.txt) |
| GO | [GO:0003723](http://www.ebi.ac.uk/QuickGO/GTerm?id=GO:0003723) | RNA binding | 50 | [2.2e-03 ( 9)](http://genome2d.molgenrug.nl/genome2d_results/GSEA_pro/193.136.177.1i6fcn62stkevgvfg07il3k7hj1826/GSEA_Pro.single_list.GO:0003723.txt) |
| GO | [GO:0000049](http://www.ebi.ac.uk/QuickGO/GTerm?id=GO:0000049) | tRNA binding | 6 | [4.6e-02 ( 2)](http://genome2d.molgenrug.nl/genome2d_results/GSEA_pro/193.136.177.1i6fcn62stkevgvfg07il3k7hj1826/GSEA_Pro.single_list.GO:0000049.txt) |
| GO | [GO:0003824](http://www.ebi.ac.uk/QuickGO/GTerm?id=GO:0003824) | catalytic activity | 213 | [2.2e-03 (22)](http://genome2d.molgenrug.nl/genome2d_results/GSEA_pro/193.136.177.1i6fcn62stkevgvfg07il3k7hj1826/GSEA_Pro.single_list.GO:0003824.txt) |
| GO | [GO:0004252](http://www.ebi.ac.uk/QuickGO/GTerm?id=GO:0004252) | serine-type endopeptidase activity | 13 | [3.6e-02 ( 3)](http://genome2d.molgenrug.nl/genome2d_results/GSEA_pro/193.136.177.1i6fcn62stkevgvfg07il3k7hj1826/GSEA_Pro.single_list.GO:0004252.txt) |
| GO | [GO:0005215](http://www.ebi.ac.uk/QuickGO/GTerm?id=GO:0005215) | transporter activity | 53 | [6.9e-05 (12)](http://genome2d.molgenrug.nl/genome2d_results/GSEA_pro/193.136.177.1i6fcn62stkevgvfg07il3k7hj1826/GSEA_Pro.single_list.GO:0005215.txt) |
| GO | [GO:0005524](http://www.ebi.ac.uk/QuickGO/GTerm?id=GO:0005524) | ATP binding | 198 | [4.9e-04 (23)](http://genome2d.molgenrug.nl/genome2d_results/GSEA_pro/193.136.177.1i6fcn62stkevgvfg07il3k7hj1826/GSEA_Pro.single_list.GO:0005524.txt) |
| GO | [GO:0005618](http://www.ebi.ac.uk/QuickGO/GTerm?id=GO:0005618) | cell wall | 8 | [1.1e-02 ( 3)](http://genome2d.molgenrug.nl/genome2d_results/GSEA_pro/193.136.177.1i6fcn62stkevgvfg07il3k7hj1826/GSEA_Pro.single_list.GO:0005618.txt) |
| GO | [GO:0005737](http://www.ebi.ac.uk/QuickGO/GTerm?id=GO:0005737) | cytoplasm | 100 | [1.9e-04 (16)](http://genome2d.molgenrug.nl/genome2d_results/GSEA_pro/193.136.177.1i6fcn62stkevgvfg07il3k7hj1826/GSEA_Pro.single_list.GO:0005737.txt) |
| GO | [GO:0005975](http://www.ebi.ac.uk/QuickGO/GTerm?id=GO:0005975) | carbohydrate metabolic process | 40 | [5.1e-04 ( 9)](http://genome2d.molgenrug.nl/genome2d_results/GSEA_pro/193.136.177.1i6fcn62stkevgvfg07il3k7hj1826/GSEA_Pro.single_list.GO:0005975.txt) |
| GO | [GO:0006207](http://www.ebi.ac.uk/QuickGO/GTerm?id=GO:0006207) | 'de novo' pyrimidine nucleobase biosynthetic process | 5 | [3.4e-02 ( 2)](http://genome2d.molgenrug.nl/genome2d_results/GSEA_pro/193.136.177.1i6fcn62stkevgvfg07il3k7hj1826/GSEA_Pro.single_list.GO:0006207.txt) |
| GO | [GO:0006418](http://www.ebi.ac.uk/QuickGO/GTerm?id=GO:0006418) | tRNA aminoacylation for protein translation | 16 | [2.5e-03 ( 5)](http://genome2d.molgenrug.nl/genome2d_results/GSEA_pro/193.136.177.1i6fcn62stkevgvfg07il3k7hj1826/GSEA_Pro.single_list.GO:0006418.txt) |
| GO | [GO:0007155](http://www.ebi.ac.uk/QuickGO/GTerm?id=GO:0007155) | cell adhesion | 9 | [1.5e-02 ( 3)](http://genome2d.molgenrug.nl/genome2d_results/GSEA_pro/193.136.177.1i6fcn62stkevgvfg07il3k7hj1826/GSEA_Pro.single_list.GO:0007155.txt) |
| GO | [GO:0008236](http://www.ebi.ac.uk/QuickGO/GTerm?id=GO:0008236) | serine-type peptidase activity | 11 | [2.4e-02 ( 3)](http://genome2d.molgenrug.nl/genome2d_results/GSEA_pro/193.136.177.1i6fcn62stkevgvfg07il3k7hj1826/GSEA_Pro.single_list.GO:0008236.txt) |
| GO | [GO:0008299](http://www.ebi.ac.uk/QuickGO/GTerm?id=GO:0008299) | isoprenoid biosynthetic process | 8 | [1.1e-02 ( 3)](http://genome2d.molgenrug.nl/genome2d_results/GSEA_pro/193.136.177.1i6fcn62stkevgvfg07il3k7hj1826/GSEA_Pro.single_list.GO:0008299.txt) |
| GO | [GO:0006633](http://www.ebi.ac.uk/QuickGO/GTerm?id=GO:0006633) | fatty acid biosynthetic process | 11 | [4.4e-03 ( 4)](http://genome2d.molgenrug.nl/genome2d_results/GSEA_pro/193.136.177.1i6fcn62stkevgvfg07il3k7hj1826/GSEA_Pro.single_list.GO:0006633.txt) |
| GO | [GO:0009405](http://www.ebi.ac.uk/QuickGO/GTerm?id=GO:0009405) | pathogenesis | 37 | [1.7e-02 ( 6)](http://genome2d.molgenrug.nl/genome2d_results/GSEA_pro/193.136.177.1i6fcn62stkevgvfg07il3k7hj1826/GSEA_Pro.single_list.GO:0009405.txt) |
| GO | [GO:0009116](http://www.ebi.ac.uk/QuickGO/GTerm?id=GO:0009116) | nucleoside metabolic process | 11 | [2.4e-02 ( 3)](http://genome2d.molgenrug.nl/genome2d_results/GSEA_pro/193.136.177.1i6fcn62stkevgvfg07il3k7hj1826/GSEA_Pro.single_list.GO:0009116.txt) |
| GO | [GO:0009228](http://www.ebi.ac.uk/QuickGO/GTerm?id=GO:0009228) | thiamine biosynthetic process | 4 | [2.3e-02 ( 2)](http://genome2d.molgenrug.nl/genome2d_results/GSEA_pro/193.136.177.1i6fcn62stkevgvfg07il3k7hj1826/GSEA_Pro.single_list.GO:0009228.txt) |
| GO | [GO:0015171](http://www.ebi.ac.uk/QuickGO/GTerm?id=GO:0015171) | amino acid transmembrane transporter activity | 12 | [5.2e-03 ( 4)](http://genome2d.molgenrug.nl/genome2d_results/GSEA_pro/193.136.177.1i6fcn62stkevgvfg07il3k7hj1826/GSEA_Pro.single_list.GO:0015171.txt) |
| GO | [GO:0015992](http://www.ebi.ac.uk/QuickGO/GTerm?id=GO:0015992) | proton transport | 5 | [3.4e-02 ( 2)](http://genome2d.molgenrug.nl/genome2d_results/GSEA_pro/193.136.177.1i6fcn62stkevgvfg07il3k7hj1826/GSEA_Pro.single_list.GO:0015992.txt) |
| GO | [GO:0016021](http://www.ebi.ac.uk/QuickGO/GTerm?id=GO:0016021) | integral component of membrane | 158 | [1.9e-04 (21)](http://genome2d.molgenrug.nl/genome2d_results/GSEA_pro/193.136.177.1i6fcn62stkevgvfg07il3k7hj1826/GSEA_Pro.single_list.GO:0016021.txt) |
| GO | [GO:0016491](http://www.ebi.ac.uk/QuickGO/GTerm?id=GO:0016491) | oxidoreductase activity | 103 | [4.6e-03 (13)](http://genome2d.molgenrug.nl/genome2d_results/GSEA_pro/193.136.177.1i6fcn62stkevgvfg07il3k7hj1826/GSEA_Pro.single_list.GO:0016491.txt) |
| GO | [GO:0016616](http://www.ebi.ac.uk/QuickGO/GTerm?id=GO:0016616) | oxidoreductase activity, acting on the CH-OH group of donors, NAD or NADP as acceptor | 16 | [2.5e-03 ( 5)](http://genome2d.molgenrug.nl/genome2d_results/GSEA_pro/193.136.177.1i6fcn62stkevgvfg07il3k7hj1826/GSEA_Pro.single_list.GO:0016616.txt) |
| GO | [GO:0016627](http://www.ebi.ac.uk/QuickGO/GTerm?id=GO:0016627) | oxidoreductase activity, acting on the CH-CH group of donors | 6 | [4.6e-02 ( 2)](http://genome2d.molgenrug.nl/genome2d_results/GSEA_pro/193.136.177.1i6fcn62stkevgvfg07il3k7hj1826/GSEA_Pro.single_list.GO:0016627.txt) |
| GO | [GO:0016763](http://www.ebi.ac.uk/QuickGO/GTerm?id=GO:0016763) | transferase activity, transferring pentosyl groups | 5 | [3.4e-02 ( 2)](http://genome2d.molgenrug.nl/genome2d_results/GSEA_pro/193.136.177.1i6fcn62stkevgvfg07il3k7hj1826/GSEA_Pro.single_list.GO:0016763.txt) |
| GO | [GO:0050662](http://www.ebi.ac.uk/QuickGO/GTerm?id=GO:0050662) | coenzyme binding | 10 | [1.9e-02 ( 3)](http://genome2d.molgenrug.nl/genome2d_results/GSEA_pro/193.136.177.1i6fcn62stkevgvfg07il3k7hj1826/GSEA_Pro.single_list.GO:0050662.txt) |
| GO | [GO:0051715](http://www.ebi.ac.uk/QuickGO/GTerm?id=GO:0051715) | cytolysis in other organism | 10 | [1.9e-02 ( 3)](http://genome2d.molgenrug.nl/genome2d_results/GSEA_pro/193.136.177.1i6fcn62stkevgvfg07il3k7hj1826/GSEA_Pro.single_list.GO:0051715.txt) |
| GO | [GO:0055085](http://www.ebi.ac.uk/QuickGO/GTerm?id=GO:0055085) | transmembrane transport | 67 | [1.6e-03 (11)](http://genome2d.molgenrug.nl/genome2d_results/GSEA_pro/193.136.177.1i6fcn62stkevgvfg07il3k7hj1826/GSEA_Pro.single_list.GO:0055085.txt) |
| GO | [GO:0055114](http://www.ebi.ac.uk/QuickGO/GTerm?id=GO:0055114) | oxidation-reduction process | 169 | [2.0e-03 (19)](http://genome2d.molgenrug.nl/genome2d_results/GSEA_pro/193.136.177.1i6fcn62stkevgvfg07il3k7hj1826/GSEA_Pro.single_list.GO:0055114.txt) |
| IPR | [IPR000600](http://www.ebi.ac.uk/interpro/entry/IPR000600) | ROK | 2 | [0.0046 ( 2)](http://genome2d.molgenrug.nl/genome2d_results/GSEA_pro/193.136.177.1i6fcn62stkevgvfg07il3k7hj1826/GSEA_Pro.single_list.IPR000600.txt) |
| IPR | [IPR000793](http://www.ebi.ac.uk/interpro/entry/IPR000793) | ATPase, F1/V1/A1 complex, alpha/beta subunit, C-terminal | 2 | [0.0046 ( 2)](http://genome2d.molgenrug.nl/genome2d_results/GSEA_pro/193.136.177.1i6fcn62stkevgvfg07il3k7hj1826/GSEA_Pro.single_list.IPR000793.txt) |
| IPR | [IPR001209](http://www.ebi.ac.uk/interpro/entry/IPR001209) | Ribosomal protein S14 | 2 | [0.0046 ( 2)](http://genome2d.molgenrug.nl/genome2d_results/GSEA_pro/193.136.177.1i6fcn62stkevgvfg07il3k7hj1826/GSEA_Pro.single_list.IPR001209.txt) |
| IPR | [IPR001236](http://www.ebi.ac.uk/interpro/entry/IPR001236) | Lactate/malate dehydrogenase, N-terminal | 2 | [0.0046 ( 2)](http://genome2d.molgenrug.nl/genome2d_results/GSEA_pro/193.136.177.1i6fcn62stkevgvfg07il3k7hj1826/GSEA_Pro.single_list.IPR001236.txt) |
| IPR | [IPR001557](http://www.ebi.ac.uk/interpro/entry/IPR001557) | L-lactate/malate dehydrogenase | 2 | [0.0046 ( 2)](http://genome2d.molgenrug.nl/genome2d_results/GSEA_pro/193.136.177.1i6fcn62stkevgvfg07il3k7hj1826/GSEA_Pro.single_list.IPR001557.txt) |
| IPR | [IPR001734](http://www.ebi.ac.uk/interpro/entry/IPR001734) | Sodium/solute symporter | 2 | [0.0046 ( 2)](http://genome2d.molgenrug.nl/genome2d_results/GSEA_pro/193.136.177.1i6fcn62stkevgvfg07il3k7hj1826/GSEA_Pro.single_list.IPR001734.txt) |
| IPR | [IPR002312](http://www.ebi.ac.uk/interpro/entry/IPR002312) | Aspartyl/Asparaginyl-tRNA synthetase, class IIb | 2 | [0.0046 ( 2)](http://genome2d.molgenrug.nl/genome2d_results/GSEA_pro/193.136.177.1i6fcn62stkevgvfg07il3k7hj1826/GSEA_Pro.single_list.IPR002312.txt) |
| IPR | [IPR004100](http://www.ebi.ac.uk/interpro/entry/IPR004100) | ATPase, F1 complex alpha/beta subunit, N-terminal domain | 2 | [0.0046 ( 2)](http://genome2d.molgenrug.nl/genome2d_results/GSEA_pro/193.136.177.1i6fcn62stkevgvfg07il3k7hj1826/GSEA_Pro.single_list.IPR004100.txt) |
| IPR | [IPR004237](http://www.ebi.ac.uk/interpro/entry/IPR004237) | Fibronectin binding repeat | 2 | [0.0046 ( 2)](http://genome2d.molgenrug.nl/genome2d_results/GSEA_pro/193.136.177.1i6fcn62stkevgvfg07il3k7hj1826/GSEA_Pro.single_list.IPR004237.txt) |
| IPR | [IPR006042](http://www.ebi.ac.uk/interpro/entry/IPR006042) | Xanthine/uracil permease | 2 | [0.0046 ( 2)](http://genome2d.molgenrug.nl/genome2d_results/GSEA_pro/193.136.177.1i6fcn62stkevgvfg07il3k7hj1826/GSEA_Pro.single_list.IPR006042.txt) |
| IPR | [IPR006206](http://www.ebi.ac.uk/interpro/entry/IPR006206) | Mevalonate/galactokinase | 2 | [0.0046 ( 2)](http://genome2d.molgenrug.nl/genome2d_results/GSEA_pro/193.136.177.1i6fcn62stkevgvfg07il3k7hj1826/GSEA_Pro.single_list.IPR006206.txt) |
| IPR | [IPR013833](http://www.ebi.ac.uk/interpro/entry/IPR013833) | Cytochrome c oxidase, subunit III, 4-helical bundle | 2 | [0.0046 ( 2)](http://genome2d.molgenrug.nl/genome2d_results/GSEA_pro/193.136.177.1i6fcn62stkevgvfg07il3k7hj1826/GSEA_Pro.single_list.IPR013833.txt) |
| IPR | [IPR009812](http://www.ebi.ac.uk/interpro/entry/IPR009812) | Protein of unknown function DUF1381 | 2 | [0.0046 ( 2)](http://genome2d.molgenrug.nl/genome2d_results/GSEA_pro/193.136.177.1i6fcn62stkevgvfg07il3k7hj1826/GSEA_Pro.single_list.IPR009812.txt) |
| IPR | [IPR008995](http://www.ebi.ac.uk/interpro/entry/IPR008995) | Molybdate/tungstate binding, C-terminal | 2 | [0.0046 ( 2)](http://genome2d.molgenrug.nl/genome2d_results/GSEA_pro/193.136.177.1i6fcn62stkevgvfg07il3k7hj1826/GSEA_Pro.single_list.IPR008995.txt) |
| IPR | [IPR011304](http://www.ebi.ac.uk/interpro/entry/IPR011304) | L-lactate dehydrogenase | 2 | [0.0046 ( 2)](http://genome2d.molgenrug.nl/genome2d_results/GSEA_pro/193.136.177.1i6fcn62stkevgvfg07il3k7hj1826/GSEA_Pro.single_list.IPR011304.txt) |
| IPR | [IPR015955](http://www.ebi.ac.uk/interpro/entry/IPR015955) | Lactate dehydrogenase/glycoside hydrolase, family 4, C-terminal | 2 | [0.0046 ( 2)](http://genome2d.molgenrug.nl/genome2d_results/GSEA_pro/193.136.177.1i6fcn62stkevgvfg07il3k7hj1826/GSEA_Pro.single_list.IPR015955.txt) |
| IPR | [IPR014874](http://www.ebi.ac.uk/interpro/entry/IPR014874) | Staphylcoagulase, N-terminal | 2 | [0.0046 ( 2)](http://genome2d.molgenrug.nl/genome2d_results/GSEA_pro/193.136.177.1i6fcn62stkevgvfg07il3k7hj1826/GSEA_Pro.single_list.IPR014874.txt) |
| IPR | [IPR021033](http://www.ebi.ac.uk/interpro/entry/IPR021033) | Extracellular fibrinogen binding protein, C-terminal | 2 | [0.0046 ( 2)](http://genome2d.molgenrug.nl/genome2d_results/GSEA_pro/193.136.177.1i6fcn62stkevgvfg07il3k7hj1826/GSEA_Pro.single_list.IPR021033.txt) |
| IPR | [IPR021612](http://www.ebi.ac.uk/interpro/entry/IPR021612) | Staphylococcal complement inhibitor SCIN | 3 | [0.0046 ( 3)](http://genome2d.molgenrug.nl/genome2d_results/GSEA_pro/193.136.177.1i6fcn62stkevgvfg07il3k7hj1826/GSEA_Pro.single_list.IPR021612.txt) |
| IPR | [IPR022383](http://www.ebi.ac.uk/interpro/entry/IPR022383) | Lactate/malate dehydrogenase, C-terminal | 2 | [0.0046 ( 2)](http://genome2d.molgenrug.nl/genome2d_results/GSEA_pro/193.136.177.1i6fcn62stkevgvfg07il3k7hj1826/GSEA_Pro.single_list.IPR022383.txt) |
| IPR | [IPR023253](http://www.ebi.ac.uk/interpro/entry/IPR023253) | FPRL1/chemotaxis inhibitory protein | 2 | [0.0046 ( 2)](http://genome2d.molgenrug.nl/genome2d_results/GSEA_pro/193.136.177.1i6fcn62stkevgvfg07il3k7hj1826/GSEA_Pro.single_list.IPR023253.txt) |
| IPR | [IPR029041](http://www.ebi.ac.uk/interpro/entry/IPR029041) | FAD-linked oxidoreductase-like | 2 | [0.0046 ( 2)](http://genome2d.molgenrug.nl/genome2d_results/GSEA_pro/193.136.177.1i6fcn62stkevgvfg07il3k7hj1826/GSEA_Pro.single_list.IPR029041.txt) |
| IPR | [IPR032808](http://www.ebi.ac.uk/interpro/entry/IPR032808) | SAUSA300_RS03715 | 2 | [0.0046 ( 2)](http://genome2d.molgenrug.nl/genome2d_results/GSEA_pro/193.136.177.1i6fcn62stkevgvfg07il3k7hj1826/GSEA_Pro.single_list.IPR032808.txt) |
| IPR | [IPR018177](http://www.ebi.ac.uk/interpro/entry/IPR018177) | L-lactate dehydrogenase, active site | 2 | [0.0046 ( 2)](http://genome2d.molgenrug.nl/genome2d_results/GSEA_pro/193.136.177.1i6fcn62stkevgvfg07il3k7hj1826/GSEA_Pro.single_list.IPR018177.txt) |
| IPR | [IPR018271](http://www.ebi.ac.uk/interpro/entry/IPR018271) | Ribosomal protein S14, conserved site | 2 | [0.0046 ( 2)](http://genome2d.molgenrug.nl/genome2d_results/GSEA_pro/193.136.177.1i6fcn62stkevgvfg07il3k7hj1826/GSEA_Pro.single_list.IPR018271.txt) |
| IPR | [IPR020003](http://www.ebi.ac.uk/interpro/entry/IPR020003) | ATPase, alpha/beta subunit, nucleotide-binding domain, active site | 2 | [0.0046 ( 2)](http://genome2d.molgenrug.nl/genome2d_results/GSEA_pro/193.136.177.1i6fcn62stkevgvfg07il3k7hj1826/GSEA_Pro.single_list.IPR020003.txt) |
| IPR | [IPR006043](http://www.ebi.ac.uk/interpro/entry/IPR006043) | Xanthine/uracil/vitamin C permease | 3 | [0.0098 ( 2)](http://genome2d.molgenrug.nl/genome2d_results/GSEA_pro/193.136.177.1i6fcn62stkevgvfg07il3k7hj1826/GSEA_Pro.single_list.IPR006043.txt) |
| IPR | [IPR006204](http://www.ebi.ac.uk/interpro/entry/IPR006204) | GHMP kinase N-terminal domain | 5 | [0.0046 ( 3)](http://genome2d.molgenrug.nl/genome2d_results/GSEA_pro/193.136.177.1i6fcn62stkevgvfg07il3k7hj1826/GSEA_Pro.single_list.IPR006204.txt) |
| IPR | [IPR002668](http://www.ebi.ac.uk/interpro/entry/IPR002668) | Concentrative nucleoside transporter N-terminal domain | 3 | [0.0098 ( 2)](http://genome2d.molgenrug.nl/genome2d_results/GSEA_pro/193.136.177.1i6fcn62stkevgvfg07il3k7hj1826/GSEA_Pro.single_list.IPR002668.txt) |
| IPR | [IPR000194](http://www.ebi.ac.uk/interpro/entry/IPR000194) | ATPase, F1/V1/A1 complex, alpha/beta subunit, nucleotide-binding domain | 3 | [0.0098 ( 2)](http://genome2d.molgenrug.nl/genome2d_results/GSEA_pro/193.136.177.1i6fcn62stkevgvfg07il3k7hj1826/GSEA_Pro.single_list.IPR000194.txt) |
| IPR | [IPR001254](http://www.ebi.ac.uk/interpro/entry/IPR001254) | Peptidase S1 | 4 | [0.0046 ( 3)](http://genome2d.molgenrug.nl/genome2d_results/GSEA_pro/193.136.177.1i6fcn62stkevgvfg07il3k7hj1826/GSEA_Pro.single_list.IPR001254.txt) |
| IPR | [IPR004364](http://www.ebi.ac.uk/interpro/entry/IPR004364) | Aminoacyl-tRNA synthetase, class II (D/K/N) | 3 | [0.0098 ( 2)](http://genome2d.molgenrug.nl/genome2d_results/GSEA_pro/193.136.177.1i6fcn62stkevgvfg07il3k7hj1826/GSEA_Pro.single_list.IPR004364.txt) |
| IPR | [IPR008276](http://www.ebi.ac.uk/interpro/entry/IPR008276) | Concentrative nucleoside transporter | 3 | [0.0098 ( 2)](http://genome2d.molgenrug.nl/genome2d_results/GSEA_pro/193.136.177.1i6fcn62stkevgvfg07il3k7hj1826/GSEA_Pro.single_list.IPR008276.txt) |
| IPR | [IPR008353](http://www.ebi.ac.uk/interpro/entry/IPR008353) | Peptidase S1B, exfoliative toxin | 5 | [0.0046 ( 3)](http://genome2d.molgenrug.nl/genome2d_results/GSEA_pro/193.136.177.1i6fcn62stkevgvfg07il3k7hj1826/GSEA_Pro.single_list.IPR008353.txt) |
| IPR | [IPR011657](http://www.ebi.ac.uk/interpro/entry/IPR011657) | Concentrative nucleoside transporter C-terminal domain | 3 | [0.0098 ( 2)](http://genome2d.molgenrug.nl/genome2d_results/GSEA_pro/193.136.177.1i6fcn62stkevgvfg07il3k7hj1826/GSEA_Pro.single_list.IPR011657.txt) |
| IPR | [IPR016039](http://www.ebi.ac.uk/interpro/entry/IPR016039) | Thiolase-like | 7 | [0.0046 ( 4)](http://genome2d.molgenrug.nl/genome2d_results/GSEA_pro/193.136.177.1i6fcn62stkevgvfg07il3k7hj1826/GSEA_Pro.single_list.IPR016039.txt) |
| IPR | [IPR018150](http://www.ebi.ac.uk/interpro/entry/IPR018150) | Aminoacyl-tRNA synthetase, class II (D/K/N)-like | 3 | [0.0098 ( 2)](http://genome2d.molgenrug.nl/genome2d_results/GSEA_pro/193.136.177.1i6fcn62stkevgvfg07il3k7hj1826/GSEA_Pro.single_list.IPR018150.txt) |
| IPR | [IPR013750](http://www.ebi.ac.uk/interpro/entry/IPR013750) | GHMP kinase, C-terminal domain | 5 | [0.0046 ( 3)](http://genome2d.molgenrug.nl/genome2d_results/GSEA_pro/193.136.177.1i6fcn62stkevgvfg07il3k7hj1826/GSEA_Pro.single_list.IPR013750.txt) |
| IPR | [IPR029000](http://www.ebi.ac.uk/interpro/entry/IPR029000) | Cyclophilin-like domain | 6 | [0.0046 ( 3)](http://genome2d.molgenrug.nl/genome2d_results/GSEA_pro/193.136.177.1i6fcn62stkevgvfg07il3k7hj1826/GSEA_Pro.single_list.IPR029000.txt) |
| IPR | [IPR029056](http://www.ebi.ac.uk/interpro/entry/IPR029056) | Ribokinase-like | 9 | [0.0046 ( 4)](http://genome2d.molgenrug.nl/genome2d_results/GSEA_pro/193.136.177.1i6fcn62stkevgvfg07il3k7hj1826/GSEA_Pro.single_list.IPR029056.txt) |
| IPR | [IPR000836](http://www.ebi.ac.uk/interpro/entry/IPR000836) | Phosphoribosyltransferase domain | 7 | [0.0440 ( 2)](http://genome2d.molgenrug.nl/genome2d_results/GSEA_pro/193.136.177.1i6fcn62stkevgvfg07il3k7hj1826/GSEA_Pro.single_list.IPR000836.txt) |
| IPR | [IPR000515](http://www.ebi.ac.uk/interpro/entry/IPR000515) | MetI-like domain | 29 | [0.0119 ( 5)](http://genome2d.molgenrug.nl/genome2d_results/GSEA_pro/193.136.177.1i6fcn62stkevgvfg07il3k7hj1826/GSEA_Pro.single_list.IPR000515.txt) |
| IPR | [IPR001347](http://www.ebi.ac.uk/interpro/entry/IPR001347) | Sugar isomerase (SIS) | 7 | [0.0440 ( 2)](http://genome2d.molgenrug.nl/genome2d_results/GSEA_pro/193.136.177.1i6fcn62stkevgvfg07il3k7hj1826/GSEA_Pro.single_list.IPR001347.txt) |
| IPR | [IPR001509](http://www.ebi.ac.uk/interpro/entry/IPR001509) | NAD-dependent epimerase/dehydratase, N-terminal domain | 7 | [0.0440 ( 2)](http://genome2d.molgenrug.nl/genome2d_results/GSEA_pro/193.136.177.1i6fcn62stkevgvfg07il3k7hj1826/GSEA_Pro.single_list.IPR001509.txt) |
| IPR | [IPR001996](http://www.ebi.ac.uk/interpro/entry/IPR001996) | Phosphotransferase system, IIB component, type 1 | 9 | [0.0098 ( 3)](http://genome2d.molgenrug.nl/genome2d_results/GSEA_pro/193.136.177.1i6fcn62stkevgvfg07il3k7hj1826/GSEA_Pro.single_list.IPR001996.txt) |
| IPR | [IPR002173](http://www.ebi.ac.uk/interpro/entry/IPR002173) | Carbohydrate/puine kinase, PfkB, conserved site | 5 | [0.0252 ( 2)](http://genome2d.molgenrug.nl/genome2d_results/GSEA_pro/193.136.177.1i6fcn62stkevgvfg07il3k7hj1826/GSEA_Pro.single_list.IPR002173.txt) |
| IPR | [IPR002293](http://www.ebi.ac.uk/interpro/entry/IPR002293) | Amino acid/polyamine transporter I | 12 | [0.0046 ( 4)](http://genome2d.molgenrug.nl/genome2d_results/GSEA_pro/193.136.177.1i6fcn62stkevgvfg07il3k7hj1826/GSEA_Pro.single_list.IPR002293.txt) |
| IPR | [IPR003352](http://www.ebi.ac.uk/interpro/entry/IPR003352) | Phosphotransferase system, EIIC | 16 | [0.0046 ( 5)](http://genome2d.molgenrug.nl/genome2d_results/GSEA_pro/193.136.177.1i6fcn62stkevgvfg07il3k7hj1826/GSEA_Pro.single_list.IPR003352.txt) |
| IPR | [IPR003439](http://www.ebi.ac.uk/interpro/entry/IPR003439) | ABC transporter-like | 62 | [0.0246 ( 7)](http://genome2d.molgenrug.nl/genome2d_results/GSEA_pro/193.136.177.1i6fcn62stkevgvfg07il3k7hj1826/GSEA_Pro.single_list.IPR003439.txt) |
| IPR | [IPR003501](http://www.ebi.ac.uk/interpro/entry/IPR003501) | Phosphotransferase system, EIIB component, type 2/3 | 9 | [0.0098 ( 3)](http://genome2d.molgenrug.nl/genome2d_results/GSEA_pro/193.136.177.1i6fcn62stkevgvfg07il3k7hj1826/GSEA_Pro.single_list.IPR003501.txt) |
| IPR | [IPR003593](http://www.ebi.ac.uk/interpro/entry/IPR003593) | AAA+ ATPase domain | 79 | [0.0100 ( 9)](http://genome2d.molgenrug.nl/genome2d_results/GSEA_pro/193.136.177.1i6fcn62stkevgvfg07il3k7hj1826/GSEA_Pro.single_list.IPR003593.txt) |
| IPR | [IPR003963](http://www.ebi.ac.uk/interpro/entry/IPR003963) | Bi-component toxin, staphylococci | 10 | [0.0126 ( 3)](http://genome2d.molgenrug.nl/genome2d_results/GSEA_pro/193.136.177.1i6fcn62stkevgvfg07il3k7hj1826/GSEA_Pro.single_list.IPR003963.txt) |
| IPR | [IPR004154](http://www.ebi.ac.uk/interpro/entry/IPR004154) | Anticodon-binding | 4 | [0.0174 ( 2)](http://genome2d.molgenrug.nl/genome2d_results/GSEA_pro/193.136.177.1i6fcn62stkevgvfg07il3k7hj1826/GSEA_Pro.single_list.IPR004154.txt) |
| IPR | [IPR004365](http://www.ebi.ac.uk/interpro/entry/IPR004365) | OB-fold nucleic acid binding domain, AA-tRNA synthetase-type | 5 | [0.0252 ( 2)](http://genome2d.molgenrug.nl/genome2d_results/GSEA_pro/193.136.177.1i6fcn62stkevgvfg07il3k7hj1826/GSEA_Pro.single_list.IPR004365.txt) |
| IPR | [IPR004841](http://www.ebi.ac.uk/interpro/entry/IPR004841) | Amino acid permease/ SLC12A domain | 5 | [0.0252 ( 2)](http://genome2d.molgenrug.nl/genome2d_results/GSEA_pro/193.136.177.1i6fcn62stkevgvfg07il3k7hj1826/GSEA_Pro.single_list.IPR004841.txt) |
| IPR | [IPR005877](http://www.ebi.ac.uk/interpro/entry/IPR005877) | YSIRK Gram-positive signal peptide | 16 | [0.0409 ( 3)](http://genome2d.molgenrug.nl/genome2d_results/GSEA_pro/193.136.177.1i6fcn62stkevgvfg07il3k7hj1826/GSEA_Pro.single_list.IPR005877.txt) |
| IPR | [IPR008256](http://www.ebi.ac.uk/interpro/entry/IPR008256) | Peptidase S1B | 7 | [0.0056 ( 3)](http://genome2d.molgenrug.nl/genome2d_results/GSEA_pro/193.136.177.1i6fcn62stkevgvfg07il3k7hj1826/GSEA_Pro.single_list.IPR008256.txt) |
| IPR | [IPR009003](http://www.ebi.ac.uk/interpro/entry/IPR009003) | Trypsin-like cysteine/serine peptidase domain | 9 | [0.0098 ( 3)](http://genome2d.molgenrug.nl/genome2d_results/GSEA_pro/193.136.177.1i6fcn62stkevgvfg07il3k7hj1826/GSEA_Pro.single_list.IPR009003.txt) |
| IPR | [IPR008966](http://www.ebi.ac.uk/interpro/entry/IPR008966) | Adhesion domain | 7 | [0.0440 ( 2)](http://genome2d.molgenrug.nl/genome2d_results/GSEA_pro/193.136.177.1i6fcn62stkevgvfg07il3k7hj1826/GSEA_Pro.single_list.IPR008966.txt) |
| IPR | [IPR010978](http://www.ebi.ac.uk/interpro/entry/IPR010978) | tRNA-binding arm | 6 | [0.0363 ( 2)](http://genome2d.molgenrug.nl/genome2d_results/GSEA_pro/193.136.177.1i6fcn62stkevgvfg07il3k7hj1826/GSEA_Pro.single_list.IPR010978.txt) |
| IPR | [IPR011252](http://www.ebi.ac.uk/interpro/entry/IPR011252) | Fibrogen-binding domain 1 | 7 | [0.0440 ( 2)](http://genome2d.molgenrug.nl/genome2d_results/GSEA_pro/193.136.177.1i6fcn62stkevgvfg07il3k7hj1826/GSEA_Pro.single_list.IPR011252.txt) |
| IPR | [IPR011266](http://www.ebi.ac.uk/interpro/entry/IPR011266) | Fibrinogen-binding domain 2 | 7 | [0.0440 ( 2)](http://genome2d.molgenrug.nl/genome2d_results/GSEA_pro/193.136.177.1i6fcn62stkevgvfg07il3k7hj1826/GSEA_Pro.single_list.IPR011266.txt) |
| IPR | [IPR011611](http://www.ebi.ac.uk/interpro/entry/IPR011611) | Carbohydrate kinase PfkB | 5 | [0.0252 ( 2)](http://genome2d.molgenrug.nl/genome2d_results/GSEA_pro/193.136.177.1i6fcn62stkevgvfg07il3k7hj1826/GSEA_Pro.single_list.IPR011611.txt) |
| IPR | [IPR012340](http://www.ebi.ac.uk/interpro/entry/IPR012340) | Nucleic acid-binding, OB-fold | 38 | [0.0046 ( 7)](http://genome2d.molgenrug.nl/genome2d_results/GSEA_pro/193.136.177.1i6fcn62stkevgvfg07il3k7hj1826/GSEA_Pro.single_list.IPR012340.txt) |
| IPR | [IPR014721](http://www.ebi.ac.uk/interpro/entry/IPR014721) | Ribosomal protein S5 domain 2-type fold, subgroup | 13 | [0.0046 ( 4)](http://genome2d.molgenrug.nl/genome2d_results/GSEA_pro/193.136.177.1i6fcn62stkevgvfg07il3k7hj1826/GSEA_Pro.single_list.IPR014721.txt) |
| IPR | [IPR016040](http://www.ebi.ac.uk/interpro/entry/IPR016040) | NAD(P)-binding domain | 78 | [0.0252 ( 8)](http://genome2d.molgenrug.nl/genome2d_results/GSEA_pro/193.136.177.1i6fcn62stkevgvfg07il3k7hj1826/GSEA_Pro.single_list.IPR016040.txt) |
| IPR | [IPR016183](http://www.ebi.ac.uk/interpro/entry/IPR016183) | Leukocidin/porin | 10 | [0.0126 ( 3)](http://genome2d.molgenrug.nl/genome2d_results/GSEA_pro/193.136.177.1i6fcn62stkevgvfg07il3k7hj1826/GSEA_Pro.single_list.IPR016183.txt) |
| IPR | [IPR018113](http://www.ebi.ac.uk/interpro/entry/IPR018113) | Phosphotransferase system EIIB, cysteine phosphorylation site | 8 | [0.0085 ( 3)](http://genome2d.molgenrug.nl/genome2d_results/GSEA_pro/193.136.177.1i6fcn62stkevgvfg07il3k7hj1826/GSEA_Pro.single_list.IPR018113.txt) |
| IPR | [IPR019931](http://www.ebi.ac.uk/interpro/entry/IPR019931) | LPXTG cell wall anchor domain | 16 | [0.0046 ( 5)](http://genome2d.molgenrug.nl/genome2d_results/GSEA_pro/193.136.177.1i6fcn62stkevgvfg07il3k7hj1826/GSEA_Pro.single_list.IPR019931.txt) |
| IPR | [IPR019948](http://www.ebi.ac.uk/interpro/entry/IPR019948) | Gram-positive anchor | 13 | [0.0046 ( 4)](http://genome2d.molgenrug.nl/genome2d_results/GSEA_pro/193.136.177.1i6fcn62stkevgvfg07il3k7hj1826/GSEA_Pro.single_list.IPR019948.txt) |
| IPR | [IPR020568](http://www.ebi.ac.uk/interpro/entry/IPR020568) | Ribosomal protein S5 domain 2-type fold | 16 | [0.0087 ( 4)](http://genome2d.molgenrug.nl/genome2d_results/GSEA_pro/193.136.177.1i6fcn62stkevgvfg07il3k7hj1826/GSEA_Pro.single_list.IPR020568.txt) |
| IPR | [IPR028301](http://www.ebi.ac.uk/interpro/entry/IPR028301) | Serine proteases, V8 family, histidine active site | 7 | [0.0056 ( 3)](http://genome2d.molgenrug.nl/genome2d_results/GSEA_pro/193.136.177.1i6fcn62stkevgvfg07il3k7hj1826/GSEA_Pro.single_list.IPR028301.txt) |
| IPR | [IPR029752](http://www.ebi.ac.uk/interpro/entry/IPR029752) | D-isomer specific 2-hydroxyacid dehydrogenase, NAD-binding domain conserved site 1 | 5 | [0.0252 ( 2)](http://genome2d.molgenrug.nl/genome2d_results/GSEA_pro/193.136.177.1i6fcn62stkevgvfg07il3k7hj1826/GSEA_Pro.single_list.IPR029752.txt) |
| Pfam | [PF02874](http://pfam.xfam.org/family/PF02874) | ATP synthase alpha/beta family, beta-barrel domain | 2 | [0.00080 (2)](http://genome2d.molgenrug.nl/genome2d_results/GSEA_pro/193.136.177.1i6fcn62stkevgvfg07il3k7hj1826/GSEA_Pro.single_list.PF02874.txt) |
| Pfam | [PF02866](http://pfam.xfam.org/family/PF02866) | lactate/malate dehydrogenase, alpha/beta C-terminal domain | 2 | [0.00080 (2)](http://genome2d.molgenrug.nl/genome2d_results/GSEA_pro/193.136.177.1i6fcn62stkevgvfg07il3k7hj1826/GSEA_Pro.single_list.PF02866.txt) |
| Pfam | [PF11546](http://pfam.xfam.org/family/PF11546) | Staphylococcal complement inhibitor SCIN | 3 | [0.00031 (3)](http://genome2d.molgenrug.nl/genome2d_results/GSEA_pro/193.136.177.1i6fcn62stkevgvfg07il3k7hj1826/GSEA_Pro.single_list.PF11546.txt) |
| Pfam | [PF12199](http://pfam.xfam.org/family/PF12199) | Extracellular fibrinogen binding protein C terminal | 2 | [0.00080 (2)](http://genome2d.molgenrug.nl/genome2d_results/GSEA_pro/193.136.177.1i6fcn62stkevgvfg07il3k7hj1826/GSEA_Pro.single_list.PF12199.txt) |
| Pfam | [PF13416](http://pfam.xfam.org/family/PF13416) | Bacterial extracellular solute-binding protein | 2 | [0.00080 (2)](http://genome2d.molgenrug.nl/genome2d_results/GSEA_pro/193.136.177.1i6fcn62stkevgvfg07il3k7hj1826/GSEA_Pro.single_list.PF13416.txt) |
| Pfam | [PF07681](http://pfam.xfam.org/family/PF07681) | SAUSA300_RS03715 | 2 | [0.00080 (2)](http://genome2d.molgenrug.nl/genome2d_results/GSEA_pro/193.136.177.1i6fcn62stkevgvfg07il3k7hj1826/GSEA_Pro.single_list.PF07681.txt) |
| Pfam | [PF07129](http://pfam.xfam.org/family/PF07129) | SAUSA300_RS07725 | 2 | [0.00080 (2)](http://genome2d.molgenrug.nl/genome2d_results/GSEA_pro/193.136.177.1i6fcn62stkevgvfg07il3k7hj1826/GSEA_Pro.single_list.PF07129.txt) |
| Pfam | [PF02986](http://pfam.xfam.org/family/PF02986) | Fibronectin binding repeat | 2 | [0.00080 (2)](http://genome2d.molgenrug.nl/genome2d_results/GSEA_pro/193.136.177.1i6fcn62stkevgvfg07il3k7hj1826/GSEA_Pro.single_list.PF02986.txt) |
| Pfam | [PF08764](http://pfam.xfam.org/family/PF08764) | Staphylococcus aureus coagulase | 2 | [0.00080 (2)](http://genome2d.molgenrug.nl/genome2d_results/GSEA_pro/193.136.177.1i6fcn62stkevgvfg07il3k7hj1826/GSEA_Pro.single_list.PF08764.txt) |
| Pfam | [PF00474](http://pfam.xfam.org/family/PF00474) | Sodium:solute symporter family | 2 | [0.00080 (2)](http://genome2d.molgenrug.nl/genome2d_results/GSEA_pro/193.136.177.1i6fcn62stkevgvfg07il3k7hj1826/GSEA_Pro.single_list.PF00474.txt) |
| Pfam | [PF00480](http://pfam.xfam.org/family/PF00480) | ROK family | 2 | [0.00080 (2)](http://genome2d.molgenrug.nl/genome2d_results/GSEA_pro/193.136.177.1i6fcn62stkevgvfg07il3k7hj1826/GSEA_Pro.single_list.PF00480.txt) |
| Pfam | [PF00056](http://pfam.xfam.org/family/PF00056) | lactate/malate dehydrogenase, NAD binding domain | 2 | [0.00080 (2)](http://genome2d.molgenrug.nl/genome2d_results/GSEA_pro/193.136.177.1i6fcn62stkevgvfg07il3k7hj1826/GSEA_Pro.single_list.PF00056.txt) |
| Pfam | [PF00253](http://pfam.xfam.org/family/PF00253) | Ribosomal protein S14p/S29e | 2 | [0.00080 (2)](http://genome2d.molgenrug.nl/genome2d_results/GSEA_pro/193.136.177.1i6fcn62stkevgvfg07il3k7hj1826/GSEA_Pro.single_list.PF00253.txt) |
| Pfam | [PF00089](http://pfam.xfam.org/family/PF00089) | Trypsin | 4 | [0.00060 (3)](http://genome2d.molgenrug.nl/genome2d_results/GSEA_pro/193.136.177.1i6fcn62stkevgvfg07il3k7hj1826/GSEA_Pro.single_list.PF00089.txt) |
| Pfam | [PF00367](http://pfam.xfam.org/family/PF00367) | phosphotransferase system, EIIB | 8 | [0.00080 (3)](http://genome2d.molgenrug.nl/genome2d_results/GSEA_pro/193.136.177.1i6fcn62stkevgvfg07il3k7hj1826/GSEA_Pro.single_list.PF00367.txt) |
| Pfam | [PF00006](http://pfam.xfam.org/family/PF00006) | ATP synthase alpha/beta family, nucleotide-binding domain | 3 | [0.00184 (2)](http://genome2d.molgenrug.nl/genome2d_results/GSEA_pro/193.136.177.1i6fcn62stkevgvfg07il3k7hj1826/GSEA_Pro.single_list.PF00006.txt) |
| Pfam | [PF00152](http://pfam.xfam.org/family/PF00152) | tRNA synthetases class II (D, K and N) | 3 | [0.00184 (2)](http://genome2d.molgenrug.nl/genome2d_results/GSEA_pro/193.136.177.1i6fcn62stkevgvfg07il3k7hj1826/GSEA_Pro.single_list.PF00152.txt) |
| Pfam | [PF00288](http://pfam.xfam.org/family/PF00288) | GHMP kinases N terminal domain | 5 | [0.00060 (3)](http://genome2d.molgenrug.nl/genome2d_results/GSEA_pro/193.136.177.1i6fcn62stkevgvfg07il3k7hj1826/GSEA_Pro.single_list.PF00288.txt) |
| Pfam | [PF00860](http://pfam.xfam.org/family/PF00860) | Permease family | 3 | [0.00184 (2)](http://genome2d.molgenrug.nl/genome2d_results/GSEA_pro/193.136.177.1i6fcn62stkevgvfg07il3k7hj1826/GSEA_Pro.single_list.PF00860.txt) |
| Pfam | [PF01773](http://pfam.xfam.org/family/PF01773) | Na+ dependent nucleoside transporter N-terminus | 3 | [0.00184 (2)](http://genome2d.molgenrug.nl/genome2d_results/GSEA_pro/193.136.177.1i6fcn62stkevgvfg07il3k7hj1826/GSEA_Pro.single_list.PF01773.txt) |
| Pfam | [PF02378](http://pfam.xfam.org/family/PF02378) | Phosphotransferase system, EIIC | 12 | [0.00060 (4)](http://genome2d.molgenrug.nl/genome2d_results/GSEA_pro/193.136.177.1i6fcn62stkevgvfg07il3k7hj1826/GSEA_Pro.single_list.PF02378.txt) |
| Pfam | [PF03129](http://pfam.xfam.org/family/PF03129) | Anticodon binding domain | 4 | [0.00350 (2)](http://genome2d.molgenrug.nl/genome2d_results/GSEA_pro/193.136.177.1i6fcn62stkevgvfg07il3k7hj1826/GSEA_Pro.single_list.PF03129.txt) |
| Pfam | [PF07662](http://pfam.xfam.org/family/PF07662) | Na+ dependent nucleoside transporter C-terminus | 3 | [0.00184 (2)](http://genome2d.molgenrug.nl/genome2d_results/GSEA_pro/193.136.177.1i6fcn62stkevgvfg07il3k7hj1826/GSEA_Pro.single_list.PF07662.txt) |
| Pfam | [PF08544](http://pfam.xfam.org/family/PF08544) | GHMP kinases C terminal | 5 | [0.00060 (3)](http://genome2d.molgenrug.nl/genome2d_results/GSEA_pro/193.136.177.1i6fcn62stkevgvfg07il3k7hj1826/GSEA_Pro.single_list.PF08544.txt) |
| Pfam | [PF10425](http://pfam.xfam.org/family/PF10425) | C-terminus of bacterial fibrinogen-binding adhesin | 7 | [0.00847 (2)](http://genome2d.molgenrug.nl/genome2d_results/GSEA_pro/193.136.177.1i6fcn62stkevgvfg07il3k7hj1826/GSEA_Pro.single_list.PF10425.txt) |
| Pfam | [PF04650](http://pfam.xfam.org/family/PF04650) | SAUSA300_RS00585 | 15 | [0.00415 (3)](http://genome2d.molgenrug.nl/genome2d_results/GSEA_pro/193.136.177.1i6fcn62stkevgvfg07il3k7hj1826/GSEA_Pro.single_list.PF04650.txt) |
| Pfam | [PF07690](http://pfam.xfam.org/family/PF07690) | Major Facilitator Superfamily | 27 | [0.01549 (3)](http://genome2d.molgenrug.nl/genome2d_results/GSEA_pro/193.136.177.1i6fcn62stkevgvfg07il3k7hj1826/GSEA_Pro.single_list.PF07690.txt) |
| Pfam | [PF07968](http://pfam.xfam.org/family/PF07968) | Leukocidin/Hemolysin toxin family | 10 | [0.00155 (3)](http://genome2d.molgenrug.nl/genome2d_results/GSEA_pro/193.136.177.1i6fcn62stkevgvfg07il3k7hj1826/GSEA_Pro.single_list.PF07968.txt) |
| Pfam | [PF13520](http://pfam.xfam.org/family/PF13520) | Amino acid permease | 7 | [0.00847 (2)](http://genome2d.molgenrug.nl/genome2d_results/GSEA_pro/193.136.177.1i6fcn62stkevgvfg07il3k7hj1826/GSEA_Pro.single_list.PF13520.txt) |
| Pfam | [PF01032](http://pfam.xfam.org/family/PF01032) | FecCD transport family | 10 | [0.01549 (2)](http://genome2d.molgenrug.nl/genome2d_results/GSEA_pro/193.136.177.1i6fcn62stkevgvfg07il3k7hj1826/GSEA_Pro.single_list.PF01032.txt) |
| Pfam | [PF01336](http://pfam.xfam.org/family/PF01336) | OB-fold nucleic acid binding domain | 5 | [0.00504 (2)](http://genome2d.molgenrug.nl/genome2d_results/GSEA_pro/193.136.177.1i6fcn62stkevgvfg07il3k7hj1826/GSEA_Pro.single_list.PF01336.txt) |
| Pfam | [PF01370](http://pfam.xfam.org/family/PF01370) | NAD dependent epimerase/dehydratase family | 7 | [0.00847 (2)](http://genome2d.molgenrug.nl/genome2d_results/GSEA_pro/193.136.177.1i6fcn62stkevgvfg07il3k7hj1826/GSEA_Pro.single_list.PF01370.txt) |
| Pfam | [PF01380](http://pfam.xfam.org/family/PF01380) | SIS domain | 7 | [0.00847 (2)](http://genome2d.molgenrug.nl/genome2d_results/GSEA_pro/193.136.177.1i6fcn62stkevgvfg07il3k7hj1826/GSEA_Pro.single_list.PF01380.txt) |
| Pfam | [PF01381](http://pfam.xfam.org/family/PF01381) | Helix-turn-helix | 10 | [0.01549 (2)](http://genome2d.molgenrug.nl/genome2d_results/GSEA_pro/193.136.177.1i6fcn62stkevgvfg07il3k7hj1826/GSEA_Pro.single_list.PF01381.txt) |
| Pfam | [PF01479](http://pfam.xfam.org/family/PF01479) | S4 domain | 7 | [0.00847 (2)](http://genome2d.molgenrug.nl/genome2d_results/GSEA_pro/193.136.177.1i6fcn62stkevgvfg07il3k7hj1826/GSEA_Pro.single_list.PF01479.txt) |
| Pfam | [PF01497](http://pfam.xfam.org/family/PF01497) | Periplasmic binding protein | 8 | [0.01087 (2)](http://genome2d.molgenrug.nl/genome2d_results/GSEA_pro/193.136.177.1i6fcn62stkevgvfg07il3k7hj1826/GSEA_Pro.single_list.PF01497.txt) |
| Pfam | [PF02302](http://pfam.xfam.org/family/PF02302) | PTS system, Lactose/Cellobiose specific IIB subunit | 7 | [0.00847 (2)](http://genome2d.molgenrug.nl/genome2d_results/GSEA_pro/193.136.177.1i6fcn62stkevgvfg07il3k7hj1826/GSEA_Pro.single_list.PF02302.txt) |
| Pfam | [PF00005](http://pfam.xfam.org/family/PF00005) | ABC transporter | 62 | [0.00080 (7)](http://genome2d.molgenrug.nl/genome2d_results/GSEA_pro/193.136.177.1i6fcn62stkevgvfg07il3k7hj1826/GSEA_Pro.single_list.PF00005.txt) |
| Pfam | [PF00156](http://pfam.xfam.org/family/PF00156) | Phosphoribosyl transferase domain | 7 | [0.00847 (2)](http://genome2d.molgenrug.nl/genome2d_results/GSEA_pro/193.136.177.1i6fcn62stkevgvfg07il3k7hj1826/GSEA_Pro.single_list.PF00156.txt) |
| Pfam | [PF00294](http://pfam.xfam.org/family/PF00294) | pfkB family carbohydrate kinase | 5 | [0.00504 (2)](http://genome2d.molgenrug.nl/genome2d_results/GSEA_pro/193.136.177.1i6fcn62stkevgvfg07il3k7hj1826/GSEA_Pro.single_list.PF00294.txt) |
| Pfam | [PF00324](http://pfam.xfam.org/family/PF00324) | Amino acid permease | 5 | [0.00504 (2)](http://genome2d.molgenrug.nl/genome2d_results/GSEA_pro/193.136.177.1i6fcn62stkevgvfg07il3k7hj1826/GSEA_Pro.single_list.PF00324.txt) |
| Pfam | [PF00359](http://pfam.xfam.org/family/PF00359) | Phosphoenolpyruvate-dependent sugar phosphotransferase system, EIIA 2 | 9 | [0.01347 (2)](http://genome2d.molgenrug.nl/genome2d_results/GSEA_pro/193.136.177.1i6fcn62stkevgvfg07il3k7hj1826/GSEA_Pro.single_list.PF00359.txt) |
| Pfam | [PF00528](http://pfam.xfam.org/family/PF00528) | Binding-protein-dependent transport system inner membrane component | 29 | [0.00080 (5)](http://genome2d.molgenrug.nl/genome2d_results/GSEA_pro/193.136.177.1i6fcn62stkevgvfg07il3k7hj1826/GSEA_Pro.single_list.PF00528.txt) |
| Pfam | [PF00746](http://pfam.xfam.org/family/PF00746) | SAUSA300_RS00585 | 13 | [0.00060 (4)](http://genome2d.molgenrug.nl/genome2d_results/GSEA_pro/193.136.177.1i6fcn62stkevgvfg07il3k7hj1826/GSEA_Pro.single_list.PF00746.txt) |
| SMART | [SM00382](http://smart.embl-heidelberg.de/smart/do_annotation.pl?BLAST=DUMMY&DOMAIN=SM00382) | ATPases associated with a variety of cellular activities | 79 | [0.00000 (9)](http://genome2d.molgenrug.nl/genome2d_results/GSEA_pro/193.136.177.1i6fcn62stkevgvfg07il3k7hj1826/GSEA_Pro.single_list.SM00382.txt) |
| SMART | [SM00363](http://smart.embl-heidelberg.de/smart/do_annotation.pl?BLAST=DUMMY&DOMAIN=SM00363) | S4 RNA-binding domain | 7 | [0.00084 (2)](http://genome2d.molgenrug.nl/genome2d_results/GSEA_pro/193.136.177.1i6fcn62stkevgvfg07il3k7hj1826/GSEA_Pro.single_list.SM00363.txt) |
| SUPERFAMILY | [SSF101094](http://supfam.cs.bris.ac.uk/SUPERFAMILY/cgi-bin/scop.cgi?ipid=SSF101094) | Staphylocoagulase | 2 | [1.4e-03 ( 2)](http://genome2d.molgenrug.nl/genome2d_results/GSEA_pro/193.136.177.1i6fcn62stkevgvfg07il3k7hj1826/GSEA_Pro.single_list.SSF101094.txt) |
| SUPERFAMILY | [SSF158366](http://supfam.cs.bris.ac.uk/SUPERFAMILY/cgi-bin/scop.cgi?ipid=SSF158366) | Efb C-domain-like | 2 | [1.4e-03 ( 2)](http://genome2d.molgenrug.nl/genome2d_results/GSEA_pro/193.136.177.1i6fcn62stkevgvfg07il3k7hj1826/GSEA_Pro.single_list.SSF158366.txt) |
| SUPERFAMILY | [SSF47917](http://supfam.cs.bris.ac.uk/SUPERFAMILY/cgi-bin/scop.cgi?ipid=SSF47917) | C-terminal domain of alpha and beta subunits of F1 ATP synthase | 2 | [1.4e-03 ( 2)](http://genome2d.molgenrug.nl/genome2d_results/GSEA_pro/193.136.177.1i6fcn62stkevgvfg07il3k7hj1826/GSEA_Pro.single_list.SSF47917.txt) |
| SUPERFAMILY | [SSF50331](http://supfam.cs.bris.ac.uk/SUPERFAMILY/cgi-bin/scop.cgi?ipid=SSF50331) | MOP-like | 2 | [1.4e-03 ( 2)](http://genome2d.molgenrug.nl/genome2d_results/GSEA_pro/193.136.177.1i6fcn62stkevgvfg07il3k7hj1826/GSEA_Pro.single_list.SSF50331.txt) |
| SUPERFAMILY | [SSF50615](http://supfam.cs.bris.ac.uk/SUPERFAMILY/cgi-bin/scop.cgi?ipid=SSF50615) | N-terminal domain of alpha and beta subunits of F1 ATP synthase | 2 | [1.4e-03 ( 2)](http://genome2d.molgenrug.nl/genome2d_results/GSEA_pro/193.136.177.1i6fcn62stkevgvfg07il3k7hj1826/GSEA_Pro.single_list.SSF50615.txt) |
| SUPERFAMILY | [SSF51730](http://supfam.cs.bris.ac.uk/SUPERFAMILY/cgi-bin/scop.cgi?ipid=SSF51730) | FAD-linked oxidoreductase | 2 | [1.4e-03 ( 2)](http://genome2d.molgenrug.nl/genome2d_results/GSEA_pro/193.136.177.1i6fcn62stkevgvfg07il3k7hj1826/GSEA_Pro.single_list.SSF51730.txt) |
| SUPERFAMILY | [SSF53901](http://supfam.cs.bris.ac.uk/SUPERFAMILY/cgi-bin/scop.cgi?ipid=SSF53901) | Thiolase-like | 7 | [2.3e-04 ( 4)](http://genome2d.molgenrug.nl/genome2d_results/GSEA_pro/193.136.177.1i6fcn62stkevgvfg07il3k7hj1826/GSEA_Pro.single_list.SSF53901.txt) |
| SUPERFAMILY | [SSF55681](http://supfam.cs.bris.ac.uk/SUPERFAMILY/cgi-bin/scop.cgi?ipid=SSF55681) | Class II aaRS and biotin synthetases | 17 | [2.3e-06 ( 7)](http://genome2d.molgenrug.nl/genome2d_results/GSEA_pro/193.136.177.1i6fcn62stkevgvfg07il3k7hj1826/GSEA_Pro.single_list.SSF55681.txt) |
| SUPERFAMILY | [SSF56327](http://supfam.cs.bris.ac.uk/SUPERFAMILY/cgi-bin/scop.cgi?ipid=SSF56327) | LDH C-terminal domain-like | 2 | [1.4e-03 ( 2)](http://genome2d.molgenrug.nl/genome2d_results/GSEA_pro/193.136.177.1i6fcn62stkevgvfg07il3k7hj1826/GSEA_Pro.single_list.SSF56327.txt) |
| SUPERFAMILY | [SSF55060](http://supfam.cs.bris.ac.uk/SUPERFAMILY/cgi-bin/scop.cgi?ipid=SSF55060) | GHMP Kinase, C-terminal domain | 5 | [8.3e-04 ( 3)](http://genome2d.molgenrug.nl/genome2d_results/GSEA_pro/193.136.177.1i6fcn62stkevgvfg07il3k7hj1826/GSEA_Pro.single_list.SSF55060.txt) |
| SUPERFAMILY | [SSF50891](http://supfam.cs.bris.ac.uk/SUPERFAMILY/cgi-bin/scop.cgi?ipid=SSF50891) | Cyclophilin-like | 6 | [1.3e-03 ( 3)](http://genome2d.molgenrug.nl/genome2d_results/GSEA_pro/193.136.177.1i6fcn62stkevgvfg07il3k7hj1826/GSEA_Pro.single_list.SSF50891.txt) |
| SUPERFAMILY | [SSF53613](http://supfam.cs.bris.ac.uk/SUPERFAMILY/cgi-bin/scop.cgi?ipid=SSF53613) | Ribokinase-like | 9 | [5.2e-04 ( 4)](http://genome2d.molgenrug.nl/genome2d_results/GSEA_pro/193.136.177.1i6fcn62stkevgvfg07il3k7hj1826/GSEA_Pro.single_list.SSF53613.txt) |
| SUPERFAMILY | [SSF53067](http://supfam.cs.bris.ac.uk/SUPERFAMILY/cgi-bin/scop.cgi?ipid=SSF53067) | Actin-like ATPase domain | 11 | [7.4e-04 ( 4)](http://genome2d.molgenrug.nl/genome2d_results/GSEA_pro/193.136.177.1i6fcn62stkevgvfg07il3k7hj1826/GSEA_Pro.single_list.SSF53067.txt) |
| SUPERFAMILY | [SSF52954](http://supfam.cs.bris.ac.uk/SUPERFAMILY/cgi-bin/scop.cgi?ipid=SSF52954) | Class II aaRS ABD-related | 4 | [6.2e-03 ( 2)](http://genome2d.molgenrug.nl/genome2d_results/GSEA_pro/193.136.177.1i6fcn62stkevgvfg07il3k7hj1826/GSEA_Pro.single_list.SSF52954.txt) |
| SUPERFAMILY | [SSF103473](http://supfam.cs.bris.ac.uk/SUPERFAMILY/cgi-bin/scop.cgi?ipid=SSF103473) | MFS general substrate transporter | 35 | [1.5e-02 ( 4)](http://genome2d.molgenrug.nl/genome2d_results/GSEA_pro/193.136.177.1i6fcn62stkevgvfg07il3k7hj1826/GSEA_Pro.single_list.SSF103473.txt) |
| SUPERFAMILY | [SSF161098](http://supfam.cs.bris.ac.uk/SUPERFAMILY/cgi-bin/scop.cgi?ipid=SSF161098) | MetI-like | 29 | [1.4e-03 ( 5)](http://genome2d.molgenrug.nl/genome2d_results/GSEA_pro/193.136.177.1i6fcn62stkevgvfg07il3k7hj1826/GSEA_Pro.single_list.SSF161098.txt) |
| SUPERFAMILY | [SSF46589](http://supfam.cs.bris.ac.uk/SUPERFAMILY/cgi-bin/scop.cgi?ipid=SSF46589) | tRNA-binding arm | 6 | [1.4e-02 ( 2)](http://genome2d.molgenrug.nl/genome2d_results/GSEA_pro/193.136.177.1i6fcn62stkevgvfg07il3k7hj1826/GSEA_Pro.single_list.SSF46589.txt) |
| SUPERFAMILY | [SSF49401](http://supfam.cs.bris.ac.uk/SUPERFAMILY/cgi-bin/scop.cgi?ipid=SSF49401) | Bacterial adhesins | 7 | [1.8e-02 ( 2)](http://genome2d.molgenrug.nl/genome2d_results/GSEA_pro/193.136.177.1i6fcn62stkevgvfg07il3k7hj1826/GSEA_Pro.single_list.SSF49401.txt) |
| SUPERFAMILY | [SSF50249](http://supfam.cs.bris.ac.uk/SUPERFAMILY/cgi-bin/scop.cgi?ipid=SSF50249) | Nucleic acid-binding proteins | 33 | [7.4e-04 ( 6)](http://genome2d.molgenrug.nl/genome2d_results/GSEA_pro/193.136.177.1i6fcn62stkevgvfg07il3k7hj1826/GSEA_Pro.single_list.SSF50249.txt) |
| SUPERFAMILY | [SSF50494](http://supfam.cs.bris.ac.uk/SUPERFAMILY/cgi-bin/scop.cgi?ipid=SSF50494) | Trypsin-like serine proteases | 9 | [2.2e-03 ( 3)](http://genome2d.molgenrug.nl/genome2d_results/GSEA_pro/193.136.177.1i6fcn62stkevgvfg07il3k7hj1826/GSEA_Pro.single_list.SSF50494.txt) |
| SUPERFAMILY | [SSF51735](http://supfam.cs.bris.ac.uk/SUPERFAMILY/cgi-bin/scop.cgi?ipid=SSF51735) | NAD(P)-binding Rossmann-fold domains | 72 | [1.3e-03 ( 8)](http://genome2d.molgenrug.nl/genome2d_results/GSEA_pro/193.136.177.1i6fcn62stkevgvfg07il3k7hj1826/GSEA_Pro.single_list.SSF51735.txt) |
| SUPERFAMILY | [SSF52283](http://supfam.cs.bris.ac.uk/SUPERFAMILY/cgi-bin/scop.cgi?ipid=SSF52283) | Formate/glycerate dehydrogenase catalytic domain-like | 9 | [2.5e-02 ( 2)](http://genome2d.molgenrug.nl/genome2d_results/GSEA_pro/193.136.177.1i6fcn62stkevgvfg07il3k7hj1826/GSEA_Pro.single_list.SSF52283.txt) |
| SUPERFAMILY | [SSF52794](http://supfam.cs.bris.ac.uk/SUPERFAMILY/cgi-bin/scop.cgi?ipid=SSF52794) | PTS system IIB component-like | 9 | [2.2e-03 ( 3)](http://genome2d.molgenrug.nl/genome2d_results/GSEA_pro/193.136.177.1i6fcn62stkevgvfg07il3k7hj1826/GSEA_Pro.single_list.SSF52794.txt) |
| SUPERFAMILY | [SSF53271](http://supfam.cs.bris.ac.uk/SUPERFAMILY/cgi-bin/scop.cgi?ipid=SSF53271) | PRTase-like | 10 | [2.9e-02 ( 2)](http://genome2d.molgenrug.nl/genome2d_results/GSEA_pro/193.136.177.1i6fcn62stkevgvfg07il3k7hj1826/GSEA_Pro.single_list.SSF53271.txt) |
| SUPERFAMILY | [SSF53697](http://supfam.cs.bris.ac.uk/SUPERFAMILY/cgi-bin/scop.cgi?ipid=SSF53697) | SIS domain | 8 | [2.2e-02 ( 2)](http://genome2d.molgenrug.nl/genome2d_results/GSEA_pro/193.136.177.1i6fcn62stkevgvfg07il3k7hj1826/GSEA_Pro.single_list.SSF53697.txt) |
| SUPERFAMILY | [SSF53807](http://supfam.cs.bris.ac.uk/SUPERFAMILY/cgi-bin/scop.cgi?ipid=SSF53807) | "Helical backbone" metal receptor | 10 | [2.8e-03 ( 3)](http://genome2d.molgenrug.nl/genome2d_results/GSEA_pro/193.136.177.1i6fcn62stkevgvfg07il3k7hj1826/GSEA_Pro.single_list.SSF53807.txt) |
| SUPERFAMILY | [SSF53850](http://supfam.cs.bris.ac.uk/SUPERFAMILY/cgi-bin/scop.cgi?ipid=SSF53850) | Periplasmic binding protein-like II | 28 | [3.6e-02 ( 3)](http://genome2d.molgenrug.nl/genome2d_results/GSEA_pro/193.136.177.1i6fcn62stkevgvfg07il3k7hj1826/GSEA_Pro.single_list.SSF53850.txt) |
| SUPERFAMILY | [SSF54211](http://supfam.cs.bris.ac.uk/SUPERFAMILY/cgi-bin/scop.cgi?ipid=SSF54211) | Ribosomal protein S5 domain 2-like | 16 | [1.4e-03 ( 4)](http://genome2d.molgenrug.nl/genome2d_results/GSEA_pro/193.136.177.1i6fcn62stkevgvfg07il3k7hj1826/GSEA_Pro.single_list.SSF54211.txt) |
| SUPERFAMILY | [SSF54637](http://supfam.cs.bris.ac.uk/SUPERFAMILY/cgi-bin/scop.cgi?ipid=SSF54637) | Thioesterase/thiol ester dehydrase-isomerase | 9 | [2.5e-02 ( 2)](http://genome2d.molgenrug.nl/genome2d_results/GSEA_pro/193.136.177.1i6fcn62stkevgvfg07il3k7hj1826/GSEA_Pro.single_list.SSF54637.txt) |
| SUPERFAMILY | [SSF55174](http://supfam.cs.bris.ac.uk/SUPERFAMILY/cgi-bin/scop.cgi?ipid=SSF55174) | Alpha-L RNA-binding motif | 8 | [2.2e-02 ( 2)](http://genome2d.molgenrug.nl/genome2d_results/GSEA_pro/193.136.177.1i6fcn62stkevgvfg07il3k7hj1826/GSEA_Pro.single_list.SSF55174.txt) |
| SUPERFAMILY | [SSF55604](http://supfam.cs.bris.ac.uk/SUPERFAMILY/cgi-bin/scop.cgi?ipid=SSF55604) | Glucose permease domain IIB | 9 | [2.2e-03 ( 3)](http://genome2d.molgenrug.nl/genome2d_results/GSEA_pro/193.136.177.1i6fcn62stkevgvfg07il3k7hj1826/GSEA_Pro.single_list.SSF55604.txt) |
| SUPERFAMILY | [SSF57716](http://supfam.cs.bris.ac.uk/SUPERFAMILY/cgi-bin/scop.cgi?ipid=SSF57716) | Glucocorticoid receptor-like (DNA-binding domain) | 5 | [9.8e-03 ( 2)](http://genome2d.molgenrug.nl/genome2d_results/GSEA_pro/193.136.177.1i6fcn62stkevgvfg07il3k7hj1826/GSEA_Pro.single_list.SSF57716.txt) |
| SUPERFAMILY | [SSF56959](http://supfam.cs.bris.ac.uk/SUPERFAMILY/cgi-bin/scop.cgi?ipid=SSF56959) | Leukocidin-like | 10 | [2.8e-03 ( 3)](http://genome2d.molgenrug.nl/genome2d_results/GSEA_pro/193.136.177.1i6fcn62stkevgvfg07il3k7hj1826/GSEA_Pro.single_list.SSF56959.txt) |
| SUPERFAMILY | [SSF55804](http://supfam.cs.bris.ac.uk/SUPERFAMILY/cgi-bin/scop.cgi?ipid=SSF55804) | Phoshotransferase/anion transport protein | 9 | [2.5e-02 ( 2)](http://genome2d.molgenrug.nl/genome2d_results/GSEA_pro/193.136.177.1i6fcn62stkevgvfg07il3k7hj1826/GSEA_Pro.single_list.SSF55804.txt) |

^a^Databases of: Pfam, protein families; IPR, InterPro or Protein Sequence Analysis and Classification; GO, Gene Ontology; SUPERFAMILY, structural and functional protein annotations; COGs, Clusters of Orthologous Groups; KEGG, Kyoto Encyclopedia of Genes and Genomes; SMART (Simple Modular Architecture Research Tool); ^b^Database accession number or identification of classes (biological function or molecular process); ^c^Description of the class (biological function or molecular process); ^d^Total number of the genes composing the class; ^e^Between brackets are presented the numbers of genes of the class that are differentially expressed. Outside brackets are *p*-values. The light to dark blue colouring represents low to high significance, respectively. The intensity of the colour is based on (TopHits/ClassSize) * -log2 (adj-*p*-value).

**SUPPLEMENTAL FIGURES**


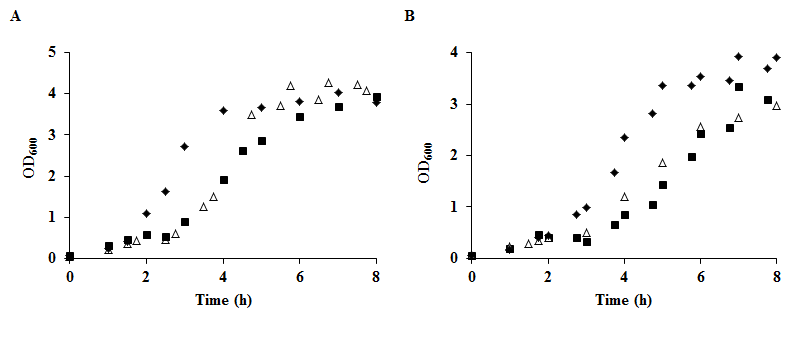


**FIGURE S1.** **Growth profiles of *S. aureus* cultures treated with NO.** Growth of *S. aureus* wild type in CDM containing 20 mM glucose **(A)** and 20 mM galactose **(B)**, at 37ºC, under aerobic conditions (150 rpm), untreated (⯁) and exposed to 250 µM (△) and 500 µM (■) spermine-NONOate added at OD_600_ 0.4. The points represent averages of at least three independent experiments.


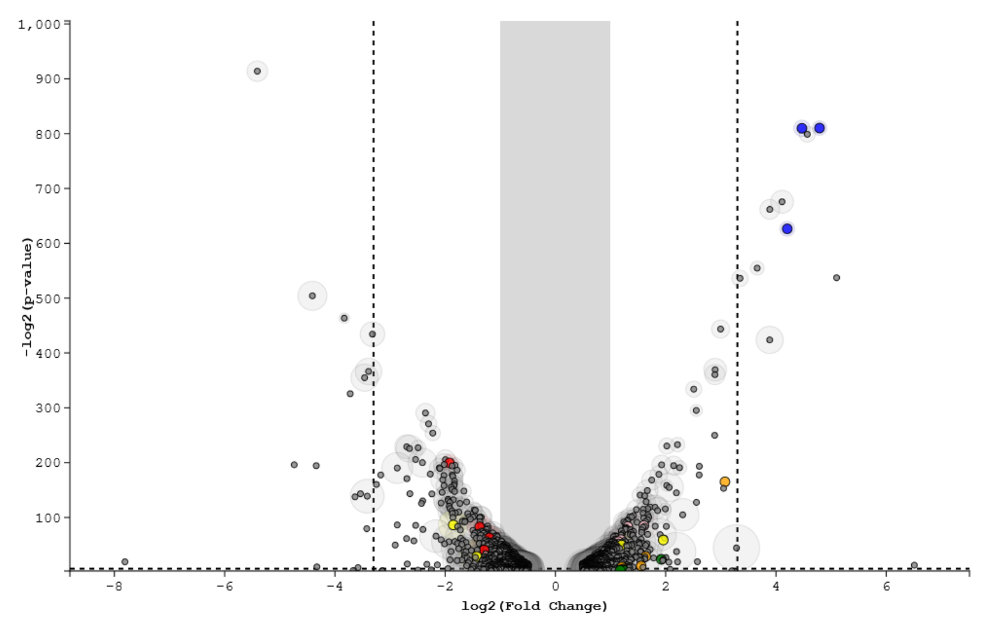


**FIGURE S2.** **Volcano plot of significant altered genes on galactose-CDM-grown *S. aureus* exposed to NO.** Points located outside the grey area indicate genes with fold change ≥ 2 or ≤ -2 with adjusted *p*-value ≤ 0.05 (Tophits); points located beyond the dotted lines indicate genes with a fold change ≥ 5 or ≤ -5 with adjusted *p*-value ≤ 0.01 (Highfold tophits). Points located in the grey area depict genes with no changes in expression.

**FIGURE S3. Threonine and glutamate consumption by *S. aureus* cells exposed to NO and grown in the absence of a glycolytic carbon source (A), on glucose (B) and on galactose (C).** Extracellular amino acid concentration in cells unexposed to NO (control, C) and exposed 1h and 3h to NO. Three independent experiments were performed and SEM is indicated for each condition.


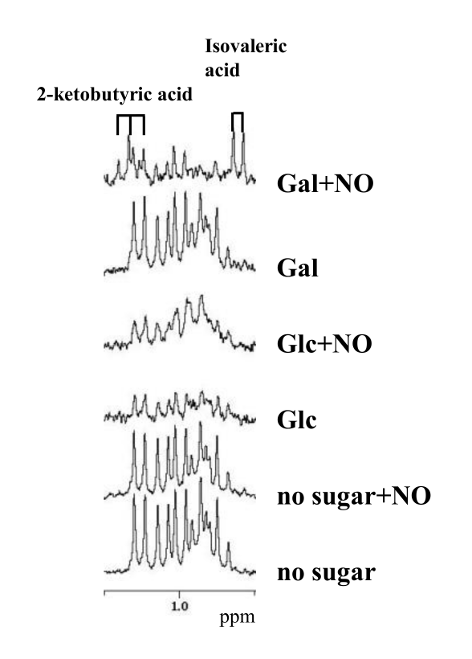


**FIGURE S4.** **^1^H-NMR spectra of supernatants of *S. aureus.*** *S. aureus* was grown on CDM in the absence of a carbon source (no sugar, no sugar+NO), supplemented with glucose (Glc, Glc+NO) or galactose (Gal, Gal+NO), which were left untreated (no sugar, Glc, Gal) or exposed to NO (no sugar+NO, Glc+NO, Gal+NO). Figure shows peaks in the 0.9-1.1 ppm region.


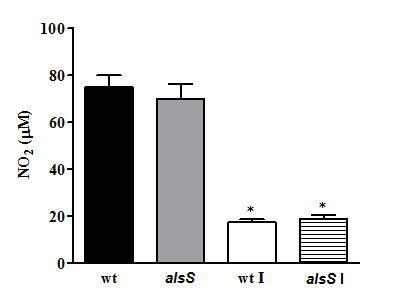


**FIGURE S5. Evaluation of nitrite content of macrophages.** Evaluation of the extracellular nitrite accumulation on supernatant of activated macrophages (black and grey bars) and treated with nitric oxide synthase inhibitor (white and striped bars). Measurements were done for *S. aureus* wild type (wt, wt I) and *alsS* mutant (*alsS*, *alsS I*), after 5 h of infection. Error bars represent the SEM of three biological samples that were assayed in triplicate. *, statistically significant (*p*-value < 0.05).
